# Treatment Patterns and Clinical Outcomes in Youth with Comorbid ADHD and PTSD: Insights from Real-World Data

**DOI:** 10.1177/10870547261416173

**Published:** 2026-02-12

**Authors:** Raman Baweja, Fabiana Lopes, Felix M Padilla, Ritika Baweja, Lisa Amaya-Jackson, Daniel A. Waschbusch, James G. Waxmonsky

**Affiliations:** 1 Penn State College of Medicine, Hershey, PA, USA; 2 Duke University School of Medicine, Dunham, NC, USA

**Keywords:** ADHD, PTSD, treatment, psychopharmacology, psychotherapy

## Abstract

**Objectives:**

Attention-deficit/hyperactivity disorder (ADHD) and post-traumatic stress disorder (PTSD) often co-occur in youth, complicating the clinical presentation. However, little is known about how PTSD influences treatment selection or outcomes in youth with ADHD. This study examined prescribing patterns and clinical outcomes among youth with ADHD, with and without comorbid PTSD.

**Methods:**

This retrospective cohort study used electronic health record data from the TriNetX Research Network, including over 714,000 youth (ages 6–18) diagnosed with ADHD (F90), of whom 30,341 (4.25%) also had comorbid PTSD (F43.1). Outcomes included treatment trends, emergency visits, hospitalizations, and subsequent antipsychotic or mood stabilizer prescriptions. Relative risks (RR), hazard ratios (HR), and 95% confidence intervals were calculated using propensity score matching and Cox proportional hazards models adjusted for sociodemographic and psychiatric variables.

**Results:**

Among youth with ADHD, those with comorbid PTSD were older, had more psychiatric comorbidities, and were more likely to receive non-stimulants (*RR* 1.54, 95% CI [1.51, 1.57]), antidepressants, antipsychotics, mood stabilizers (RRs 1.29–1.70), and psychotherapy (*RR* 1.55, 95% CI [1.51, 1.60]). Methylphenidate prescriptions were slightly lower (*RR* 0.97, 95% CI [0.95, 0.99]), while amphetamine use remained stable. Among youth with ADHD and PTSD, CNS stimulants were associated with the most favorable outcomes across all clinical measures, including hospitalizations, emergency visits, and subsequent antipsychotic and mood stabilizer use (aHRs 0.52–0.74), compared with non-stimulants and antidepressants.

**Conclusions:**

Youth with ADHD and PTSD are clinically complex and receive broader treatment interventions. Clinicians appear to de-prioritize stimulants after PTSD diagnosis, despite evidence of superior clinical outcomes. Findings underscore the need for prospective studies and evidence-based treatment guidelines for this high-risk population.

## Introduction

Attention-deficit/hyperactivity disorder (ADHD) is one of the most common neurodevelopmental disorders in youth, with a global prevalence of approximately 6% and an estimated male-to-female ratio of about 2:1 (Faraone et al., 2021). It has substantial impacts on academic, social, and emotional functioning (Baweja et al., 2015; Faraone et al., 2021). Post-traumatic stress disorder (PTSD) is also prevalent in pediatric populations, with a lifetime prevalence of 4.7%, and significantly higher rates in girls than in boys (7.3% vs. 2.2%) (McLaughlin et al., 2013). PTSD can also lead to significant impacts on children’s development, with negative consequences for socioemotional and academic functioning (Malarbi et al., 2017; Tamir et al., 2025). The co-occurrence of ADHD and PTSD is relatively common (Harrington et al., 2012). Among adults with ADHD, approximately 12% have a comorbid PTSD diagnosis, while up to 37% of youth diagnosed with PTSD also meet criteria for ADHD (Famularo et al., 1996; Kessler et al., 2006).

The bidirectional relationship between ADHD and PTSD likely reflects shared genetic liability and overlapping neurobiological mechanisms, particularly those involving executive dysfunction and arousal regulation (Martínez et al., 2016; Wendt et al., 2023). ADHD-related traits such as impulsivity and inattention may increase trauma exposure risk (Barkley, 2020; Bruce et al., 2007), while PTSD-related alterations in attention and inhibitory control can mimic or exacerbate ADHD symptoms (Adams et al., 2020; Punski-Hoogervorst et al., 2023). Furthermore, accumulating evidence indicates that the combination of ADHD and PTSD, compared to either disorder alone, is associated with higher rates of psychiatric comorbidity (Biederman et al., 2013; Gelner et al., 2023), complicating treatment planning. Young adults from diverse racial and ethnic backgrounds who had elevated ADHD symptoms in childhood reported higher levels of PTSD symptoms. This association was moderated by trauma-related arousal and depressive symptoms (Miodus et al., 2021). Notably, ADHD has been shown to increase the risk of developing PTSD threefold (Spencer et al., 2016), suggesting that the course and treatment of ADHD could influence the development of PSTD following a trauma.

Central nervous system (CNS) stimulants, such as methylphenidate and amphetamines, are the evidence-based first-line treatment for ADHD (Pliszka & AACAP Work Group on Quality Issues, 2007). However, the role of stimulants in treating PTSD symptoms remains complex. Some studies suggest that stimulant treatment for ADHD may reduce the subsequent risk of developing anxiety disorders, including PTSD (Biederman et al., 2009), and decrease the risk of unintentional injuries in youth (Ghirardi et al., 2020; L. Li et al., 2025). Conversely, evidence from studies in adults has raised concerns about a potential link between stimulant use and the onset or exacerbation of PTSD symptoms (Crum-Cianflone et al., 2015). Such findings may lead clinicians to hesitate when considering CNS stimulants for patients with co-occurring ADHD and PTSD. Overall, the evidence on the effectiveness and safety of CNS stimulants in youth with both conditions remains limited, and clear clinical guidelines for managing this comorbidity are lacking. Non-stimulant medications such as guanfacine, clonidine, and atomoxetine demonstrate promise for managing symptoms of both ADHD and PTSD but typically are less effective for reducing ADHD symptoms than CNS stimulants and require consistent daily dosing (Boodoo et al., 2022; Cortese et al., 2018; Jagtiani et al., 2024; Pliszka & AACAP Work Group on Quality Issues, 2007; Wang et al., 2022).

Treatment guidelines for children recommend psychotherapy as the first-line treatment and pharmacotherapy as the second-line option for pediatric PTSD (Cohen & The AACAP Work Group On Quality Issues, 2010; B. Keeshin et al., 2020). Although two selective serotonin reuptake inhibitors (SSRIs), paroxetine (Marshall et al., 2001) and sertraline (Brady et al., 2000), are approved by the Food and Drug Administration (FDA) for treating PTSD in adults, no psychotropic medications, including SSRIs, have received FDA approval for childhood PTSD. Nevertheless, clinicians frequently prescribe these medications off-label to manage emotional and behavioral disturbances associated with childhood trauma, sometimes without the careful consideration warranted, particularly when treating the complex trauma histories (Bowden et al., 2022).

Atypical antipsychotics and mood stabilizers are typically reserved for refractory cases of pediatric PTSD, mainly to address psychotic-like symptoms in the case of atypical antipsychotics (Bajor et al., 2022; B. R. Keeshin & Strawn, 2014). Antipsychotics and mood stabilizers have also shown beneficial effects for managing aggression, when associated with ADHD, after optimizing CNS stimulants and implementing psychosocial interventions (Baweja & Waxmonsky, 2022; Blader et al., 2009, 2021).

Given the limited evidence base and the wide range of potential treatment options, there is likely substantial variability in how youth with co-occurring ADHD and PTSD are managed. Pediatric PTSD research suggests that age and sex may act as treatment moderators (Danzi & La Greca, 2021); however, it remains unknown whether these factors similarly influence youth with comorbid ADHD and PTSD. Despite the growing recognition of the association between these disorders, no prior studies have systematically assessed treatment patterns or clinical outcomes in this comorbid population. Moreover, there is limited data on how treatment choices influence the course of illness, or on the factors that guide the selection and modification of treatment regimens over time.

This study examines how co-occurring PTSD influences treatment patterns in youth with ADHD, focusing on: (a) differences in prescribing practices, stratified by age, sex, race, and ethnicity, and treatment pathways between stimulant and non-stimulant ADHD medications; (b) the association of PTSD comorbidity in youth with ADHD on the use of other treatments, including antidepressants, antipsychotics, and mood stabilizers, as well as psychotherapy utilization and treatment sequencing; and (c) the relationship between treatment type and clinical outcomes in youth with comorbid ADHD and PTSD. By analyzing prescribing trends, treatment trajectories, and outcomes in a large, nationally representative, and diverse sample of youth with ADHD, this study aims to clarify how PTSD comorbidity shapes treatment selection and delivery across modalities, as well as subsequent outcomes. Gaining a deeper understanding of these patterns is essential for optimizing care strategies, reducing potential disparities, improving clinical outcomes, and informing evidence-based decision-making for this complex population.

## Methods

### Study Design

This retrospective cohort study used data from TriNetX, LLC (“TriNetX”), a global health research network providing access to electronic health records (EHRs) from healthcare organizations (HCOs) worldwide. The dataset was extracted in April 2025 from the TriNetX Research Network, which includes EHRs for approximately 137 million patients across 98 HCOs. Patients receiving any care in participating health systems are included in the dataset. These data represent a secondary analysis of de-identified EHR records in accordance with the HIPAA Privacy Rule. Accordingly, the Institutional Review Board determined that the study was exempt from review, and patient consent was not required.

### Study Sample

The study included 714,129 youth aged 6 to 18 years with a diagnosis of ADHD (ICD-10-CM F90) between 2010 and 2024. Among them, 30,341 (4.25%) also had a diagnosis of PTSD (ICD-10-CM F43.1) and were classified as the “ADHD with PTSD” group (PTSD cohort). The remaining 683,788 youth had ADHD without PTSD and were classified as the “ADHD without PTSD” group (ADHD cohort).

The sample was geographically diverse: 18.5% from the Northeast, 23.0% from the Midwest, 43.9% from the South, 9.9% from the West, 3.5% from outside the United States, and 1.3% from unknown locations. Across clinical settings, 64% had outpatient visits (including tele-visits and visits for any reason), 34% had emergency department visits, 10% had inpatient admissions, and 24% had psychiatric service utilization. Approximately 91% of patients remained in the dataset for >3 months after ADHD diagnosis, and 66% remained for >5 years. The cohorts had a mean follow-up duration of 2.35 ± 2.46 years. On average, 8,672 new patients per month met study criteria over the past 3 years. Further details are provided in the Supplemental, and the appendix includes diagnostic and procedural codes used. Medication and service utilization were identified using Current Procedural Terminology codes.

### Variables

#### Dependent Variables (Outcomes)

The primary dependent variables included treatment patterns and clinical outcomes associated with medication exposure following the index event. Clinical outcomes of interest, commonly relevant among individuals with PTSD, comprised the subsequent use of mood stabilizers or antipsychotics, as well as hospitalizations and emergency department visits. Mood stabilizers evaluated in this study included lithium, valproate, oxcarbazepine, carbamazepine, and lamotrigine (Baweja et al., 2025).

#### Independent Variables

For analyses of treatment patterns, the independent variable was PTSD status at the index event, categorized as ADHD with PTSD versus ADHD without PTSD. Although prazosin is commonly prescribed for PTSD-related nightmares, its use was highly imbalanced (8% in the PTSD cohort vs. 0.14% in the ADHD cohort) and was therefore excluded from analyses.

For analyses of clinical outcomes, independent variables included prescriptions for ADHD medications (CNS stimulants, non-stimulants, alpha-2 agonists, atomoxetine, viloxazine) and antidepressants. CNS stimulants included methylphenidate and amphetamines, while non-stimulants included clonidine, guanfacine, atomoxetine, and viloxazine (Baweja et al., 2024; Faraone et al., 2021).

For clinical outcome analyses, index events were defined based on the comparison of interest: (1) For treated versus untreated patients, the index event was the first prescription of an ADHD medication or an antidepressant following diagnosis; (2) For ADHD medication versus antidepressant comparisons, the index event was the first prescription of either an ADHD medication or an antidepressant; and (3) For CNS stimulant versus non-stimulant comparisons, the index event was the first prescription of either a CNS stimulant or a non-stimulant. For comparative analyses, exposure classifications were mutually exclusive throughout the study period. For example, within the PTSD cohort, individuals prescribed CNS stimulants could not receive non-stimulants during the study period, and vice versa.

#### Covariates

Covariates included demographic characteristics (age at index, sex, and race/ethnicity as documented in the EHRs) and psychiatric comorbidities, such as mood disorders, disruptive behavior disorders, autism spectrum disorder, tic disorders, psychotic disorders, anxiety disorders, obsessive–compulsive disorder, intellectual disabilities, eating disorders, sleep disorders, borderline personality disorder, and substance use disorders.

### Statistical Analyses

Statistical analyses were conducted in April 2025 using the TriNetX secure platform. Descriptive analyses were performed, and medication prescription patterns and psychotherapy utilization were examined across sex, age groups (6–11 years vs. 12–18 years), and racial/ethnic groups (Hispanic vs. non-Hispanic; White vs. Black).

Associations were assessed using odds ratios (ORs) with 95% confidence intervals (CIs) to compare demographic characteristics, comorbid psychiatric diagnoses, and service utilization between the ADHD and PTSD cohorts.

Propensity score matching was conducted using TriNetX’s built-in platform to address baseline imbalances in sociodemographic characteristics (age at index, sex, and race/ethnicity) and psychiatric comorbidities. Matching was performed using greedy nearest-neighbor matching with a 0.1 standard deviation caliper to achieve 1:1 matching without replacement, enhancing interpretability, preventing overrepresentation of individuals, and allowing balanced comparisons of prescription patterns and long-term outcomes (Austin, 2014). In this approach, each treated individual is sequentially paired with the closest control based on propensity scores, with previously matched controls excluded from further pairing. Detailed descriptions of matching procedures, outcomes, follow-up time, and sample sizes are provided in the Supplemental.

Relative risks (RRs) with 95% CIs were calculated to assess time-dependent outcomes, such as prescription patterns and psychotherapy utilization, following specific index events. To test the robustness of our findings, we conducted a sensitivity analysis by limiting the dataset to new prescriptions only, excluding medications that were initiated before and continued beyond the index event.

Since clonidine is frequently prescribed off-label for pediatric sleep problems (Jang et al., 2022) and is also used to treat both ADHD and PTSD (Marchi et al., 2024; Pliszka & AACAP Work Group on Quality Issues, 2007), we performed an additional sensitivity analysis excluding patients with documented sleep disorders to minimize confounding, as these individuals are more likely to have received clonidine for sleep-related issues.

Hazard ratios (HRs) with 95% CIs were used to evaluate time-to-event outcomes, such as inpatient hospitalizations or subsequent antipsychotic prescriptions, following index events in the PTSD cohort. Cox proportional hazards models adjusted for sociodemographic factors and psychiatric comorbidities, yielding adjusted hazard ratios (aHRs) representing associations between exposures and outcomes independent of confounding. To account for multiple comparisons, p-values from these models were adjusted using the Benjamini-Hochberg false discovery rate (FDR) method.

We performed an additional sensitivity analysis within the ADHD with PTSD cohort to examine acute clinical outcomes under more stringent medication exposure criteria. To be included, patients needed to have received at least two prescriptions for either a CNS stimulant or a non-stimulant within a 6-month period, with outcomes evaluated over the following 6 months. This approach was intended to reflect sustained treatment rather than short-term initiation that might be discontinued within the first month due to tolerability or other factors. The 6-month timeframe also accommodated potential interruptions in medication use, such as during school breaks (e.g., summer or winter holidays), and accounted for longer prescription durations (up to 90 days) commonly observed with non-stimulant medications.

Secondary analyses examined the association between ADHD treatment and subsequent risk of PTSD diagnosis using Cox proportional hazards models in the full cohort. In the primary comparison, exposure was receipt of any ADHD medication versus no ADHD medication, with first recorded PTSD diagnosis as the outcome. A secondary comparison contrasted CNS stimulants with non-stimulants, adjusting for sociodemographic variables (age, sex, and race/ethnicity).

Treatment pathways were captured within the TriNetX platform for youth in the PTSD cohort to reflect real-world prescribing patterns. Sequential treatment patterns were analyzed starting from the index event (the first documented diagnosis of both ADHD and PTSD) through six stages or lines of treatment (LOTs) as documented in the EHRs. At each stage, the proportion of patients receiving various treatment modalities, including ADHD medications, antidepressants, antipsychotics, mood stabilizers, and psychotherapy, was assessed. Differences in prescribing patterns between CNS stimulants and non-stimulants were also evaluated to understand how ADHD treatment preferences vary in the presence of comorbid PTSD.

## Results

### Baseline Demographic and Clinical Characteristics

The PTSD cohort was significantly older at the time of the index event, when both ADHD and PTSD diagnoses were documented, compared to the ADHD cohort (mean age: 10.8 ± 3.4 years vs. 8.5 ± 3.3 years; SMD = 0.697; *p* < .001) (Table 1). The PTSD cohort was more likely to include female youth, individuals of Black or American Indian/Alaska Native race, and those of non-Hispanic ethnicity (ORs ranging from 1.18 to 2.03). Conversely, the PTSD cohort was less likely to include Asian youth compared to the ADHD cohort (OR: 0.32; 95% CI [0.28, 0.36]).

**Table 1.** Sociodemographic, Clinical and Service Utilization Characteristics.

Sociodemographic	ADHD without PTSD; *n* = 683,788 (%)	ADHD with PTSD; *n* = 30,341 (%)	*OR*	95% CI
Sex				
Male	464,866 (68.0)	15,671 (51.7)	0.50	[0.49, 0.51]
Female	209,626 (30.7)	13,996 (46.1)	1.94	[1.89, 1.98]
Unknown	9,296 (1.3)	674 (2.2)	1.65	[1.52, 1.78]
Ethnicity				
Not Hispanic or Latino	464,292 (67.9)	21,645 (71.3)	1.18	[1.15, 1.21]
Hispanic or Latino	81,302 (11.9)	3,507 (11.6)	0.97	[0.93, 1.00]
Unknown ethnicity	138,194 (20.2)	5,189 (17.1)	0.81	[0.79, 0.84]
Race				
White	405,897 (59.4)	18,117 (59.7)	1.01	[0.99, 1.04]
Black or African America	111,457 (16.3)	6,059 (20.0)	1.28	[1.24, 1.32]
American Indian or Alaska Native	2,804 (0.4)	252 (0.8)	2.03	[1.79, 2.32]
Asian	18,804 (2.8)	270 (0.9)	0.32	[0.28, 0.36]
Native Hawaiian or other Pacific Islander	2,188 (0.3)	97 (0.3)	1.00	[0.81, 1.22]
Other	40,480 (5.9)	1,787 (5.9)	0.99	[0.95, 1.04]
Unknown race	102,158 (14.9)	3,759 (12.4)	0.81	[0.78, 0.83]
Psychiatric comorbidity				
Autism spectrum disorder	153,416 (22.4)	4,699 (15.5)	0.63	[0.61, 0.65]
Disruptive behavior disorders	115,276 (16.9)	12,422 (40.9)	3.42	[3.34, 3.50]
Mood disorders	111,885 (16.4)	18,572 (61.2)	8.07	[7.88, 8.26]
Depressive disorders (including recurrent)	79,226 (11.6)	14,203 (46.8)	6.72	[6.56, 6.88]
Bipolar disorder	7,618 (1.1)	2,358 (7.8)	7.48	[7.13, 7.84]
DMDD	21,004 (3.1)	5,124 (16.9)	6.41	[6.20, 6.63]
Anxiety disorder, unspecified	153,416 (22.4)	14,417 (47.5)	3.13	[3.06, 3.20]
Generalized anxiety disorder	61,862 (9.0)	7,824 (25.8)	3.49	[3.40, 3.59]
Panic disorder	7,399 (1.1)	1,870 (6.2)	6.00	[5.70, 6.33]
Obsessive compulsive disorder	16,653 (2.4)	1,819 (6.0)	2.55	[2.43, 2.69]
Psychotic disorders	4,064 (0.6)	1,495 (4.9)	8.67	[8.16, 9.21]
Intellectual disabilities	21,907 (3.2)	1,470 (4.8)	1.54	[1.46, 1.62]
Tic disorders	27,803 (4.1)	1,163 (3.8)	0.94	[0.89, 1.00]
Sleep disorders	25,902 (3.8)	2,928 (9.7)	2.71	[2.61, 2.82]
Eating disorders	14,758 (2.2)	1,884 (6.2)	3.00	[2.86, 3.15]
Substance use disorders	14,465 (2.1)	3,820 (12.6)	6.66	[6.42, 6.92]
Borderline personality disorder	4,884 (0.7)	1,199 (4.0)	5.72	[5.36, 6.10]
Service utilization				
Outpatient services	469,092 (68.6)	21,301 (70.2)	1.08	[1.05, 1.11]
Emergency department services	240,147 (35.1)	16,703 (55.1)	2.26	[2.21, 2.32]
Hospital inpatient/observation services	68,429 (10.0)	9,259 (30.5)	3.95	[3.85, 4.05]
Psychiatric services	155,139 (22.7)	16,602 (54.7)	4.12	[4.02, 4.21]

*Note*. ADHD = Attention-deficit/hyperactivity disorder; CI = Confidence interval; PTSD = Post-traumatic stress disorder; OR = Odd ratio.

Almost all psychiatric comorbidities were significantly more prevalent in the PTSD cohort compared to the ADHD cohort (ORs ranging from 1.54 to 8.67), with the highest odds observed for psychotic and mood disorders (Table 1). The exceptions were autism spectrum disorder, which was less prevalent in the PTSD cohort (OR: 0.63; 95% CI [0.61, 0.65]), and tic disorders, which did not differ.

The PTSD cohort utilized all types of services at significantly higher rates compared to the ADHD cohort (ORs ranging from 1.08 to 3.95). The PTSD cohort also had a greater frequency of service codes specifically for psychiatry (OR: 4.12; 95% CI [4.02, 4.21]) (Table 1).

### Treatment Pattern

The PTSD cohort was more likely to be prescribed non-stimulant medications compared to the ADHD cohort (*RR*: 1.54; 95% CI [1.51, 1.57]), including alpha-2 agonists and atomoxetine, with the largest absolute increase observed for alpha-2 agonists (+16.4%) (Table 2). They were also more likely to receive antidepressants, antipsychotics, mood stabilizers, and psychotherapy (RRs ranging from 1.29 to 1.70). However, the PTSD cohort had a slight decrease in methylphenidate prescriptions (*RR*: 0.97; 95% CI [0.95, 0.99]), while amphetamine prescriptions remained essentially unchanged.

**Table 2.** ADHD Treatment Patterns in Patients with PTSD Diagnosis—Overall and Stratified by Sex.

Medication class	Subgroups	ADHD without PTSD^a^; *n*^b^ (%)	ADHD with PTSD; *n*^b^ (%)	Absolute risk difference^c^ (%)	*RR* [95%CI]
ADHD medications	All	16,774 (56.9)	18,486 (62.7)	+5.8	1.10 [1.09, 1.12]
	Male	8,883 (58.4)	9,999 (65.8)	+7.4	1.13 [1.11, 1.15]
	Female	6,957 (56.2)	7,421 (59.9)	+3.7	1.07 [1.04, 1.09]
CNS stimulants	All	13,744 (46.6)	13,609 (46.2)	−0.4	0.99 [0.97, 1.01]
	Male	7,262 (47.8)	7,506 (49.4)	+1.6	1.03 [1.01, 1.06]
	Female	5,615 (45.3)	5,323 (43.9)	−1.4	0.95 [0.92, 0.98]
Methylphenidate	All	9,914 (33.6)	9,627 (32.7)	−0.9	0.97 [0.95, 0.99]
	Male	5,344 (35.2)	5,382 (35.4)	+0.2	1.01 [0.98, 1.04]
	Female	3,990 (32.2)	3,684 (29.7)	−2.5	0.92 [0.89, 0.96]
Amphetamine	All	6,706 (22.8)	6,583 (22.3)	−0.5	0.98 [0.95, 1.01]
	Male	3,577 (23.5)	3,623 (23.8)	+0.3	1.01 [0.97, 1.06]
	Female	2,784 (22.5)	2,616 (21.1)	−1.4	0.94 [0.90, 0.99]
Non-stimulants	All	8,677 (29.4)	13,343 (45.3)	+15.9	1.54 [1.51, 1.57]
	Male	4,964 (32.7)	7,630 (50.2)	+17.5	1.54 [1.50, 1.58]
	Female	3,349 (27.0)	5,045 (40.7)	+13.7	1.51 [1.45, 1.56]
Alpha-2 agonists	All	7,807 (26.5)	12,651 (42.9)	+16.4	1.62 [1.58, 1.66]
	Male	4,572 (30.1)	7,352 (48.4)	+18.3	1.61 [1.56, 1.66]
	Female	2,910 (23.5)	4,669 (37.7)	+14.2	1.60 [1.54, 1.67]
Clonidine	All	3,653 (12.4)	6,875 (23.3)	+10.9	1.88 [1.81, 1.95]
	Male	2,183 (14.4)	4,058 (26.7)	+12.3	1.86 [1.77, 1.95]
	Female	1,360 (11.0)	2,518 (20.3)	+9.3	1.85 [1.74, 1.97]
Guanfacine	All	5,342 (18.1)	8,254 (28.0)	+9.9	1.55 [1.50, 1.59]
	Male	3,128 (20.6)	4,866 (32.0)	+11.4	1.56 [1.50, 1.62]
	Female	1,945 (15.7)	2,927 (23.6)	+7.9	1.51 [1.43, 1.59]
Atomoxetine	All	1,579 (5.4)	1,996 (6.8)	+1.4	1.26 [1.19, 1.35]
	Male	818 (5.4)	1,067 (7.0)	+1.6	1.30 [1.19, 1.43]
	Female	743 (6.0)	833 (6.7)	+0.0	1.12 [1.02, 1.23]
Viloxazine	All	181 (0.6)	163 (0.6)	+0.0	0.90 [0.73, 1.11]
	Male	95 (0.6)	93 (0.6)	−0.2	0.98 [0.74, 1.30]
	Female	85 (0.7)	61 (0.5)	+10.6	0.72 [0.52, 1.00]
Antidepressants	All	10,921 (37.1)	14,067 (47.7)	+11.0	1.29 [1.26, 1.31]
	Male	4,697 (30.9)	6,365 (41.9)	+9.5	1.36 [1.32, 1.40]
	Female	5,622 (45.4)	6,803 (54.9)	+13.2	1.21 [1.18, 1.24]
Antipsychotics	All	5,559 (18.9)	9,456 (32.1)	+13.7	1.70 [1.65, 1.75]
	Male	2,917 (19.2)	4,994 (32.9)	+12.9	1.71 [1.65, 1.78]
	Female	2,332 (18.8)	3,927 (31.7)	+3.2	1.68 [1.61, 1.76]
Mood stabilizers^d^	All	1,895 (6.4)	2,835 (9.6)	+3.2	1.50 [1.42, 1.58]
	Male	947 (6.2)	1,434 (9.4)	+3.0	1.51 [1.40, 1.64]
	Female	890 (7.2)	1,269 (10.2)	+11.2	1.43 [1.31, 1.55]
Psychotherapy	All	5,929 (20.1)	9,210 (31.3)	+10.9	1.55 [1.51, 1.60]
	Male	2,793 (18.4)	4,457 (29.3)	+10.8	1.60 [1.53, 1.66]
	Female	2,698 (21.8)	4,040 (32.6)	+5.8	1.50 [1.44, 1.56]

*Note*. ADHD = Attention-deficit/hyperactivity disorder; CI = Confidence interval; RR = Relative risk; PTSD = Post-traumatic stress disorder.

a Reference group.

b Propensity score matching was used to adjust for demographic factors and psychiatric comorbidities; *n* = All 29,471; Male: 15,200; Female 12,389.

c Absolute Risk Difference (%) represents the difference in the proportion of patients with the outcome between the “ADHD without PTSD” and “ADHD with PTSD” subgroups, expressed in percentage points.

d Mood stabilizers: lithium, valproate, oxcarbazepine, carbamazepine, and lamotrigine.

In the sensitivity analysis (Table 3), restricted to new ADHD prescriptions across cohorts (excluding ongoing treatments), there was no difference in the overall rate of new ADHD medication prescriptions between the PTSD and ADHD cohorts. However, the PTSD cohort received fewer new stimulant prescriptions than the ADHD cohort (RRs ranging from 0.83 to 0.85) but more alpha-2 agonist prescriptions (RRs ranging from 1.44 to 1.70). Prescription patterns for all other psychotropic medications and psychotherapy utilization were consistent with the main findings.

**Table 3.** New ADHD Prescription Patterns in Patients with PTSD Diagnosis.

Medication class	ADHD without PTSD^a^ 27,570^b^; % (*n*/total)	ADHD with PTSD 27,570^b^; %(*n*/total)	Absolute risk difference^c^ (%)	*RR* [95%CI]
ADHD medications	52.9 (11,555/21,859)	52.6 (8,402/15,976)	+0.3	1.00 [0.98, 1.01]
CNS stimulants	43.6 (10,439/23,914)	36.9 (7,195/19,486)	−6.7	0.85 [0.83, 0.87]
Methylphenidate	30.9 (7,772/25,183)	26.1 (5,652/21,672)	−4.8	0.85 [0.82, 0.87]
Amphetamine	20.9 (5,381/25,750)	17.2 (4,033/23,394)	−3.7	0.83 [0.80, 0.86]
Non-stimulants	25.2 (6,075/24,160)	35.9 (6,980/19,412)	+10.7	1.43 [1.39, 1.47]
Alpha-2 agonists	22.1 (5,392/24,395)	33.6 (6,662/19,811)	+11.5	1.52 [1.48, 1.57]
Clonidine	10.4 (2,699/25,916)	17.7 (4,139/23,356)	+7.3	1.70 [1.63, 1.78]
Guanfacine	15.2 (3,885/25,568)	21.8 (4,895/22,418)	+6.6	1.44 [1.38, 1.49]
Atomoxetine	5.4 (1,471/27,186)	5.5 (1,464/26,458)	+0.1	1.02 [0.95, 1.10]
Viloxazine	0.6 (170/27,539)	0.5 (143/27,517)	−0.1	0.84 [0.67, 1.05]
Antidepressants	27.8 (6,120/22,000)	37.6 (7,489/19,894)	+9.8	1.35 [1.32, 1.39]
Antipsychotics	15.0 (3,768/25,190)	24.8 (5,683/22,937)	+9.8	1.66 [1.60, 1.72]
Mood stabilizers^d^	4.8 (1,291/26,711)	7.5 (1,966/26,298)	+2.7	1.55 [1.45, 1.66]
Psychotherapy	16.8 (4,157/24,816)	23.8 (5,281/22,173)	+7.0	1.42 [1.37, 1.47]

*Note.* ADHD = Attention-deficit/hyperactivity disorder; CI = Confidence interval; RR = Relative risk; PTSD = Post-traumatic stress disorder.

a Reference group.

b Propensity score matching was used to adjust for demographic factors and psychiatric comorbidities.

c Absolute Risk Difference (%) represents the difference in the proportion of patients with the outcome between the “ADHD without PTSD” and “ADHD with PTSD” subgroups, expressed in percentage points.

d Mood stabilizers: lithium, valproate, oxcarbazepine, carbamazepine, and lamotrigine.

In a separate sensitivity analysis excluding youth with documented sleep disorders from both cohorts, clonidine prescriptions remained significantly higher in the PTSD cohort compared to the ADHD cohort (*RR*: 1.64; 95% CI [1.58, 1.70]).

#### Sex

In sex-stratified analyses comparing the PTSD and ADHD cohorts, overall prescribing patterns were largely consistent across sexes (Table 2). However, notable exceptions emerged: CNS stimulants were prescribed significantly, though modestly, more frequently among males in the PTSD cohort compared to males in the ADHD cohort (*RR*: 1.03; 95% CI [1.01, 1.06]), while females in PTSD cohort were less likely to receive these medications than those in ADHD cohort (*RR*: 0.95; 95% CI [0.92, 0.95]).

#### Age

In age-stratified analyses, prescription patterns were generally similar for children (ages 6–11) and adolescents (ages 12–18) across most treatment modalities (Supplemental Table 1). Notable differences included CNS stimulants were prescribed more frequently to younger children with PTSD (*RR*: 1.10; 95% CI [1.05, 1.14]) compared to younger children in the ADHD cohort, whereas prescribed less among adolescents (*RR*: 0.95; 95% CI [0.94, 0.97]) in PTSD cohort versus adolescents in ADHD cohort. The relative increase in clonidine prescriptions was much higher among younger children in the PTSD cohort compared to those in the ADHD cohort, with a smaller relative difference observed between the two adolescent cohorts (*RR* = 2.16 vs. 1.80).

#### Age and Sex Subgroups

Further stratification by age and sex revealed additional subgroup differences. Among younger children, CNS stimulant prescriptions were higher for males in PTSD compared to male in ADHD cohort (*RR*: 1.07; 95% CI [1.02, 1.13]), whereas no differences were observed between cohorts for females (Supplemental Table 1). In contrast, among adolescents, stimulant prescription rates remained stable for males but were significantly lower for females in the PTSD cohort compared to the ADHD cohort (*RR*: 0.92; 95% CI [0.89, 0.95]).

#### Race and Ethnicity

Across racial and ethnic groups, prescription patterns were generally consistent with the main analysis between the PTSD and ADHD cohorts across most treatment modalities (Supplemental Table 2). A notable exception was methylphenidate: it was prescribed more frequently among Black youth (*RR*: 1.09; 95% CI [1.04, 1.15]) in the PTSD cohort as compared Black youth in ADHD Cohort and less frequently among White youth (*RR*: 0.92; 95% CI [0.90, 0.95]).

### Medication Class and Clinical Outcomes in the PTSD Cohort

Among youth in the PTSD cohort, those treated with either ADHD medications or antidepressants experienced significantly higher rates of adverse clinical outcomes, including inpatient hospitalizations, emergency department visits, and subsequent prescriptions for antipsychotics and mood stabilizers, compared to untreated individuals (aHRs ranging from 1.16 to 2.68) (Table 4).

**Table 4.** Association Between Medication Type and Long-Term Outcomes in Individuals with ADHD and PTSD: Cox Proportional Hazards Models Adjusted for Demographics and Comorbidities.

Medication type outcomes	aHR [95% CI]	*p*-value	FDR-adjusted *p*-value
ADHD medication (vs. No ADHD medication^a^)			
Inpatient hospitalization	1.47 [1.38, 1.56]	<.001	.001
Emergency visit	1.16 [1.11, 1.22]	<.001	.001
Antipsychotics	2.68 [2.52, 2.84]	<.001	.001
Mood stabilizers^b^	2.65 [2.39, 2.94]	<.001	.001
Antidepressants (vs. no antidepressants^a^)			
Inpatient hospitalization	1.94 [1.82, 2.06]	<.001	.001
Emergency visit	1.25 [1.19, 1.31]	<.001	.001
Antipsychotics	2.54 [2.42, 2.67]	<.001	.001
Mood stabilizers	2.23 [2.05, 2.43]	<.001	.001
ADHD medication (vs. antidepressants^a^)			
Inpatient hospitalization	0.62 [0.55, 0.70]	<.001	.001
Emergency visit	0.83 [0.75, 0.93]	.001	.001
Antipsychotics	0.95 [0.85, 1.06]	.368	.368
Mood stabilizers^b^	1.32 [1.09, 1.59]	.004	.004
CNS stimulants (vs. antidepressants^a^)			
Inpatient hospitalization	0.53 [0.48, 0.58]	<.001	.001
Emergency visit	0.74 [0.68, 0.80]	<.001	.001
Antipsychotics	0.58 [0.53, 0.63]	<.001	.001
Mood stabilizers^b^	0.59 [0.51, 0.69]	<.001	.001
Non-stimulants (vs. antidepressants^a^)			
Inpatient hospitalization	0.76 [0.69, 0.85]	<.001	.001
Emergency visit	0.95 [0.87, 1.04]	.259	.270
Antipsychotics	1.14 [1.04, 1.24]	.004	.004
Mood stabilizers^b^	1.45 [1.24, 1.69]	<.001	.001
CNS stimulants (vs. non-stimulants^a^)			
Inpatient hospitalization	0.69 [0.63, 0.74]	<.001	.001
Emergency visit	0.73 [0.68, 0.78]	<.001	.001
Antipsychotics	0.52 [0.48, 0.55]	<.001	.001
Mood stabilizers^b^	0.52 [0.46, 0.59]	<.001	.001

*Note*. All models were adjusted for socio-demographic (age, sex, race/ethnicity) and comorbidity. FDR-adjusted *p*-values were calculated using the Benjamini-Hochberg method to control for multiple comparisons. aHR = Adjusted hazard ratio; SE = Standard error; CI = Confidence interval.

a Reference group.

b Mood stabilizers: lithium, valproate, oxcarbazepine, carbamazepine, and lamotrigine.

When directly comparing ADHD medications with antidepressants, ADHD medications were associated with lower risks of inpatient hospitalizations and emergency visits (aHRs ranging from 0.62 to 0.83) but higher use of mood stabilizers (aHR 1.32; 95% CI [1.09, 1.59]).

Further analyses comparing CNS stimulants and non-stimulants to antidepressants revealed that CNS stimulants were associated with significantly reduced risks across all outcomes (aHRs ranging from 0.53 to 0.68). In contrast, non-stimulants as compared to antidepressants were linked to reduced hospitalizations (aHR 0.76; 95% CI [0.69, 0.85]), increased antipsychotics and mood stabilizer prescriptions (aHR ranging from 1.14 to 1.45).

Finally, compared to non-stimulants, CNS stimulants were associated with significantly lower risks across all adverse clinical outcomes examined (aHRs ranging from 0.52 to 0.73).

### Sustained ADHD Medication Exposure and Acute Clinical Outcomes in PTSD Cohort

In a sensitivity analysis, we limited the cohort to individuals with repeated prescription of CNS stimulant or non-stimulants, defined as at least two prescriptions within the 6 months preceding the index event. For this analysis, outcomes were assessed outcomes during only the following 6 months (Supplemental Table 3). This criterion included approximately 80% of the stimulant and non-stimulant subgroups. The findings remained largely consistent with those observed in the primary analysis.

### Association Between ADHD Treatment and Subsequent PTSD Diagnosis

In the full cohort, after adjusting for demographic variables, youth with ADHD who received any ADHD medication were significantly more likely to be subsequently diagnosed with PTSD compared to those who did not receive ADHD medication (aHR = 1.73; 95% CI [1.68, 1.79]). When comparing specific ADHD medication types, youth treated with CNS stimulants had a significantly lower hazard of being diagnosed with PTSD compared to those treated with non-stimulants (aHR = 0.24; 95% CI [0.23, 0.25]).

### Treatment Pathways and Medication Trends

**Figure 1.**
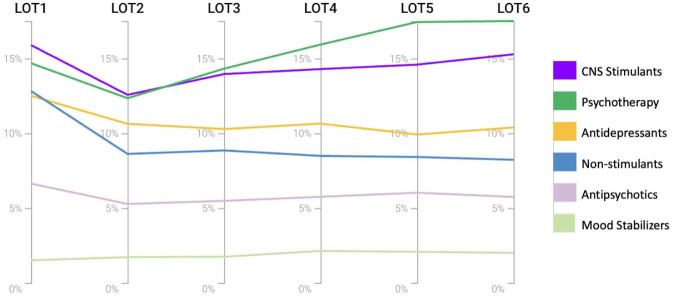
Treatment distribution across lines of treatment in individuals with ADHD and PTSD. *Note*. ADHD = Attention-deficit/hyperactivity disorder; LOT = Line of treatment; PTSD = Post-traumatic stress disorder.

In the longitudinal analysis of sequential treatment stages within the PTSD cohort, CNS stimulants and psychotherapy were the most frequently utilized treatments, with psychotherapy use gradually increasing over time. At the first stage, approximately 12% to 13% of patients received non-stimulants or antidepressants. In later stages, non-stimulant use declined and stabilized around 8%, while antidepressant use decreased to about 10%. Antipsychotic prescriptions remained steady at approximately 6% across all stages, and mood stabilizer use was consistently low and unchanged (Figure 1).

The longitudinal comparison between the PTSD and ADHD cohorts showed broadly similar stimulant prescribing patterns. Methylphenidate was the most commonly prescribed CNS stimulant in both groups, comprising about 50% of stimulant use at the initial treatment stage (Supplemental Figure 2). Non-stimulant prescribing differed: guanfacine was more frequent in the ADHD cohort (55% vs. 49%), whereas clonidine use was higher in the PTSD cohort (39% vs. 30%). Atomoxetine use varied across stages and was more common in later stages, accounting for about 20% of non-stimulant prescriptions (Supplemental Figure 3).

## Discussion

This large-scale, retrospective analysis of over 700,000 youth provides novel insight into how comorbid PTSD alters the clinical trajectory of youth with ADHD. This study observed significant differences in pharmacologic treatment patterns, and clinical outcomes between those with ADHD with and without PTSD. The diagnosis of PTSD in youth with ADHD shifted treatment patterns from CNS stimulants to non-stimulants, despite CNS stimulants being associated with lower rates of adverse clinical outcomes. These findings highlight the complex clinical needs of this population and carry important implications for assessment, treatment planning, and engagement with the health system.

Youth with comorbid ADHD and PTSD were more likely to be female, older, and from racially minoritized backgrounds. These demographic patterns are consistent with prior research demonstrating a higher prevalence of trauma exposure and psychiatric comorbidities, such as PTSD and ADHD, among racially minoritized youth (Thompson et al., 2023). Such disparities likely reflect inequities in exposure to adversity, systemic barriers to timely mental health care, and variations in diagnostic practices (Roberts et al., 2011). The higher prevalence of PTSD among females, which our findings also replicate, is well documented (Boodoo et al., 2022; McLaughlin et al., 2013) and may reflect a combination of biological vulnerability, sex-based differences in trauma exposure and symptom expression, as well as potential clinician bias in the diagnostic classification of overlapping symptoms such as irritability and hyperarousal.

A diagnosis of PTSD in youth with ADHD was associated with a marked increase in clinical complexity compared to those with ADHD without PTSD. These youth exhibited significantly higher rates of psychiatric comorbidities, greater utilization of mental health services, and elevated rates of hospitalization and emergency department visits. These findings are consistent with prior literature linking PTSD to more functional impairment than ADHD alone (Biederman et al., 2013; Gelner et al., 2023). Together, the data suggest that PTSD in the context of ADHD signals greater psychiatric burden and severity, warranting heightened attention in both assessment and treatment. These findings support the need for trauma-informed approaches to ADHD care. Routine screening for trauma related disorders should be integrated into standard ADHD evaluations, particularly for older girls and racially minoritized youth who may be at higher risk for underdiagnosed PTSD. Importantly, environmental contexts can also shape clinical presentation—for example, youth involved in maltreatment investigations or placed in foster care may experience such high levels of stress and perceived chaos that difficulties with focus and attention reflect environmental instability rather than underlying diagnostic symptoms (Sege et al., 2017). Therefore, a thorough assessment of the child’s environment across settings is advised before diagnosing ADHD in a child with appreciable trauma exposures, especially for those displaying prominent symptoms of PTSD.

The presence of a PTSD diagnosis appreciably influenced treatment patterns. Youth with comorbid PTSD had higher rates of prescriptions for non-stimulant ADHD medications, antidepressants, antipsychotics, mood stabilizers, and psychotherapy. In contrast, PTSD was associated with reduced methylphenidate use, while amphetamine prescribing remained stable. Specifically, new CNS stimulant prescriptions declined by approximately 7% following a PTSD diagnosis. These shifts may reflect clinician concerns that stimulants could exacerbate trauma-related symptoms, such as hyperarousal, although the evidence on this risk remains limited and mixed (Crum-Cianflone et al., 2015; Houlihan, 2011). The relatively stable use of non-stimulant ADHD medications after a PTSD diagnosis suggests that the decline in stimulant prescribing was not due to deprioritization of ADHD symptom treatment in favor of addressing PTSD or other non-ADHD symptoms. Instead, the observed trends reflect a shift toward non-stimulant and adjunctive treatment approaches when managing co-occurring ADHD and PTSD.

While rates of antipsychotic and mood stabilizer prescriptions increased following a PTSD diagnosis, it is encouraging that CNS stimulants and psychotherapy remained the most commonly used treatments. This pattern aligns with clinical guidelines and the established efficacy of these interventions for managing co-occurring ADHD and PTSD (Cohen & The AACAP Work Group On Quality Issues, 2010; Cortese et al., 2018; B. Keeshin et al., 2020; Pliszka & AACAP Work Group on Quality Issues, 2007). Antipsychotics and mood stabilizers are typically reserved for treatment-resistant or more severe presentations (Bajor et al., 2022; B. R. Keeshin & Strawn, 2014). However, in this analysis, these medications were frequently initiated early in the treatment course despite a lack of an evidence base to support their selection as initial treatment options.

Methylphenidate was the most commonly prescribed CNS stimulant regardless of PTSD status. Although amphetamines have been associated with a higher risk of psychosis and irritability compared to methylphenidate (Moran et al., 2019; Stuckelman et al., 2017), surprisingly it was methylphenidate, rather than amphetamines, that was more affected by a PTSD diagnosis. While hallucinations and irritability can occur as part of complex PTSD presentations, the relatively lesser impact on amphetamine prescriptions suggests that clinicians may not view these risks as a major concern, perhaps reflecting the overall low incidence of stimulant-induced psychosis in youth.

Guanfacine was the most commonly prescribed non-stimulant in both groups, consistent with previous research showing that it is the most frequently used non-stimulant for ADHD, particularly in the presence of psychiatric comorbidities (Baweja et al., 2025). In contrast, clonidine was more frequently prescribed in youth with PTSD, likely to address trauma-related hyperarousal symptoms (Strawn & Geracioti, 2008). Clonidine is also commonly used for sleep disturbances, particularly in younger children (Schnoes et al., 2006). Its elevated use in youth with PTSD, even after adjusting for diagnosed sleep disorders, suggests clinicians select it for other reasons, possibly such as reducing hyperarousal, which is a core symptoms of PTSD (Jagtiani et al., 2024), though such off-label prescribing warrants careful clinical oversight.

Among non-ADHD medications, antidepressants were the most prescribed medication across both groups, with a 29% relative increase in the PTSD cohort. This increase may reflect their proven effectiveness for PTSD in adults and their recommendation as a treatment option for youth after psychotherapy (Brady et al., 2000; Cohen & The AACAP Work Group On Quality Issues, 2010; Marshall et al., 2001). Antipsychotics showed the largest relative increase, approximately 70% higher in the PTSD group, potentially indicating efforts to manage hyperarousal, aggression, and psychotic-like symptoms sometimes observed in PTSD (Bajor et al., 2022; B. R. Keeshin & Strawn, 2014). Similar to patterns seen in other populations with elevated rates of hyperarousal and aggression, clinicians appear to prefer antipsychotics over mood stabilizers in more treatment-resistant or clinically complex cases (Baweja et al., 2025).

Despite historical concerns about stimulant use in trauma-exposed populations (Crum-Cianflone et al., 2015), our findings suggest that CNS stimulants were associated with more favorable outcomes than non-stimulants or antidepressants in this comorbid presentation. Stimulant use was associated with lower rates of hospitalization, emergency department visits, and subsequent prescriptions of antipsychotics and mood stabilizers. These results are consistent with emerging data from population-based ADHD studies (Taipale et al., 2024; Vasiliadis et al., 2024) and with literature on stimulant effects in addressing aggression associated with ADHD (Baweja & Waxmonsky, 2022; Blader et al., 2009, 2021; Towbin et al., 2020), though prior research has not focused on comorbid PTSD. Notably, youth treated with stimulants were less likely to eventually be diagnosed with PTSD, raising the possibility that stimulant treatment may mitigate trauma-related symptom expression (Houlihan, 2011). Furthermore, CNS stimulants have been shown to reduce the risk of traumatic events, including motor vehicle accidents and unintentional injuries, in youth (Ghirardi et al., 2020; L. Li et al., 2025). However, the association does not establish causality. Prospective studies are needed to determine whether stimulant use offers protective benefits against the emergence of PTSD (Kern et al., 2022). While cautious prescribing remains appropriate for trauma-exposed populations, our findings suggest that, with careful monitoring, stimulants may be both safe and potentially protective in this context.

Despite overall lower treatment utilization across modalities and medication classes among racially and ethnically minoritized youth, we observed a relatively greater increase in ADHD-related treatment, including methylphenidate, after PTSD diagnosis in these populations compared to non-Hispanic White youth. The additional impairment associated with PTSD may increase parental or provider motivation to initiate or intensify medication treatment. Differences in prescribing patterns may also reflect perceptions of efficacy or tolerability concerns in racially and ethnically minoritized youth. Notably, antidepressant use increased most among Black youth, while antipsychotic use rose more modestly among Hispanic youth following a PTSD diagnosis. These findings suggest that PTSD comorbidity may prompt greater treatment intensification in minoritized youth with ADHD. Nevertheless, persistent disparities in overall access and utilization remain, underscoring the ongoing need to address systemic barriers, including provider bias, cultural mistrust, and structural inequities in care access (E. T. Li et al., 2022).

Currently, treatment approaches for comorbid ADHD and PTSD vary widely and remain largely clinician-dependent, likely due to the absence of standardized guidelines. Our findings underscore the urgent need for evidence-based protocols that integrate pharmacologic and psychosocial interventions while addressing the complex interplay of attentional deficits, hyperactivity, hyperarousal, trauma-related dysregulation, and co-occurring mood or anxiety symptoms. Developing and validating these frameworks should be a research priority to better support this vulnerable and underrecognized population.

Despite the strengths of a large sample size and robust analytic methods, this study has several limitations inherent to retrospective analyses of EHRs. Diagnostic coding practices may vary across sites and providers, potentially leading to misclassification or underdiagnosis. Additionally, while prescribing data reflect treatment decisions, they do not capture actual medication adherence, treatment response, or symptom severity, or the type of psychotherapy. Clinician intent, psychosocial factors influencing treatment selection, and patient-level information such as income, education, and foster care placement were not available, and causal inferences cannot be drawn from observational data. Selection bias is also possible, as individuals with higher functioning or less severe PTSD symptoms may have been more likely to receive stimulant prescriptions in the clinical outcomes analysis. Furthermore, some youth with ADHD have a significant history of trauma and exhibit emotional and behavioral disturbances that overlap with ADHD symptoms, even if they do not meet full criteria for PTSD; these trauma-related features may still influence treatment decisions.

Although we applied propensity score matching and Cox proportional hazards models to adjust for measured confounders, residual confounding from unmeasured variables, such as trauma severity, family context, or socioeconomic status, likely remains. The greedy nearest-neighbor matching without replacement may not achieve a globally optimal set of matches, potentially leaving some residual imbalance. The large cohort size (over 36,000 youth with ADHD and PTSD) strengthens the study by increasing statistical power and generalizability; however, it also raises the likelihood of detecting statistically significant associations with modest effect sizes. While most relative risks were substantial (e.g., *RR* = 1.54; 95% CI [1.51, 1.57]] for non-stimulants), a few reflected only modest differences (e.g., *RR* = 0.97; 95% CI [0.95, 0.99] for methylphenidate), and thus should be interpreted with caution. In addition, findings are limited to healthcare data captured within TriNetX and may not be generalizable to youth receiving care outside the included healthcare organizations.

To conclude, youth with comorbid ADHD and PTSD represent a clinically complex population, characterized by elevated psychiatric burden and increased mental health service utilization. This study demonstrates that PTSD comorbidity significantly influences ADHD treatment trajectories, with a shift away from CNS stimulants toward non-stimulants and other psychotropic medications. Notably, CNS stimulant use was associated with a lower likelihood of subsequent PTSD diagnosis. Among youth with PTSD, stimulant use was linked to more favorable clinical outcomes, suggesting a potential stabilizing or protective effect. While trauma-focused psychotherapy remains the first-line treatment for PTSD in youth, complex presentations such as prominent ADHD symptoms, often lead to adjunctive pharmacologic approaches. Future research should clarify the causal mechanisms leading to these complex presentations that trigger polypharmacy to spur treatments development targeting these specific impairments. Randomized controlled trials and prospective longitudinal studies are essential to inform best practices and optimize care for youth facing the dual challenges of ADHD and PTSD.

## Supplemental Material

sj-docx-1-jad-10.1177_10870547261416173 – Supplemental material for Treatment Patterns and Clinical Outcomes in Youth with Comorbid ADHD and PTSD: Insights from Real-World Data Supplemental material, sj-docx-1-jad-10.1177_10870547261416173 for Treatment Patterns and Clinical Outcomes in Youth with Comorbid ADHD and PTSD: Insights from Real-World Data by Raman Baweja, Fabiana Lopes, Felix M Padilla, Ritika Baweja, Lisa Amaya-Jackson, Daniel A. Waschbusch and James G. Waxmonsky in Journal of Attention Disorders
