# Supplementary material for: Treatment Patterns and Clinical Outcomes in Youth with Comorbid ADHD and PTSD: Insights from Real-World Data
Source: J Atten Disord. 2026 Feb 12;30(7):872–86. doi: 10.1177/10870547261416173 (PMC13230649; doi:10.1177/10870547261416173)
Supplement: sj-docx-1-jad-10.1177_10870547261416173 – Supplemental material for Treatment Patterns and Clinical Outcomes in Youth with Comorbid ADHD and PTSD: Insights from Real-World Data [file sj-docx-1-jad-10.1177_10870547261416173.docx]

**Supplement**

**Treatment Patterns and Clinical Outcomes in Youth with Comorbid ADHD and PTSD: Insights from Real-World Data**

**Table of contents**

| **Supplemental Tables** | | Page |
| --- | --- | --- |
| S1 | Treatment Trends by Age and Sex | 3 |
| S2 | Treatment Trends by Race and Ethnicity | 5 |
| S3 | Sensitivity Analysis – Association of Sustained ADHD Medication Exposure on Acute Clinical Outcomes in Individuals with ADHD and PTSD | 7 |
| **Supplemental Figures** | |  |
| SF1 | CNS Stimulants Distribution Across Lines of Treatment by Cohort | 8 |
| SF2 | Non-stimulants Distribution Across Lines of Treatment | 9 |
| **Appendix** | |  |
| Appendix | Diagnosis, Procedure and Medications codes | 10 |
| **Statistical Analyses** | |  |
| SA 1 | Summary Statistics for ADHD Cohort in TriNetX Database (N =714,129) | 11 |
| **Compare Outcomes Analysis** | |  |
| SA2 | ADHD Treatment Patterns in Patients with Subsequent PTSD Diagnosis  SA2.1. Cohorts: ADHD Cohort with PTSD (vs. ADHD Cohort without PTSD)  SA2.2. Cohorts: Male ADHD Cohort with PTSD (vs. Male ADHD Cohort without PTSD)  SA2.3. Cohorts: Female ADHD Cohort with PTSD (vs. Female ADHD Cohort without PTSD) | 13 |
| SA3 | Supplement Analysis 3. ADHD Prescription Patterns in Patients with Subsequent PTSD Diagnosis (Sensitivity Analysis _ Excluding youth with documented sleep disorders)  Cohorts: ADHD Cohort with PTSD (vs ADHD Cohort without PTSD) | 20 |
| SA4 | Supplement Analysis 4. New ADHD Prescription Patterns in Patients with Subsequent PTSD Diagnosis (Sensitivity Analysis)  Cohorts: ADHD Cohort with PTSD (vs ADHD Cohort without PTSD) | 22 |
| SA5 | Treatment Trends by Age and Gender  SA5.1. Cohorts: 6-11 ADHD Cohort with PTSD (vs 6-11 ADHD Cohort without PTSD)  SA5.2. Cohorts: 6-11 Male ADHD Cohort with PTSD (vs 6-11ADHD Male Cohort without PTSD)  SA5.3. Cohorts: 6-11 Female ADHD Cohort with PTSD (vs 6-11ADHD Female Cohort without PTSD)  SA5.4. Cohorts: 12-18 ADHD Cohort with PTSD (vs 12-18 ADHD Female Cohort without PTSD)  SA5.5. Cohorts: 12-18 Male ADHD Cohort with PTSD (vs 12-18 Male ADHD Cohort without PTSD)  SA5.6. Cohorts: 12-18 Female ADHD Cohort with PTSD (vs 12-18 Female ADHD Cohort without PTSD)  SA5.2. Cohorts: 15-17 Male ADHD Cohort with SUD (vs 15-17 Male ADHD Cohort without SUD) | 24 |
| SA6 | Treatment Trends by Race and Ethnicity  SA6.1. Cohorts: White ADHD Cohort with PTSD (vs White ADHD Cohort without PTSD)  SA6.2. Cohorts: Black ADHD Cohort with PTSD (vs Black ADHD Cohort without PTSD)  SA6.3. Cohorts: Non-Hispanic ADHD Cohort with PTSD (vs Non-Hispanic ADHD Cohort without PTSD)  SA6.4. Cohorts: Hispanic ADHD Cohort with PTSD (vs Hispanic ADHD Cohort without PTSD) | 34 |
| Cox Proportional Hazards Model | |  |
| SA7 | Association Between Medication Type and Long-Term Outcomes in Individuals with ADHD and PTSD: Cox Proportional Hazards Models Adjusted for Demographics and Comorbidities  SA7.1. Cohorts: ADHD Medications (vs No ADHD Medications)  SA7.2. Cohorts: Antidepressants (vs. No Antidepressants)  SA7.3. Cohorts: ADHD Medication (vs. Antidepressants)  SA7.4. Cohorts: CNS stimulants (vs. Antidepressants)  SA7.5. Cohorts: Non-Stimulants (vs. Antidepressants)  SA7.6. Cohorts: CNS Stimulants (vs. Non-Stimulants) | 41 |
| SA8 | Sensitivity Analysis – Association of Sustained ADHD Medication Exposure on Acute Clinical Outcomes in Individuals with ADHD PTSD: Cox Proportional Hazards Models Adjusted for Demographics and Comorbidities  Cohorts: CNS stimulants (vs Non-Stimulants) | 62 |
| SA9 | Association Between ADHD Treatment and Subsequent PTSD Diagnosis  SA9.1. Cohorts: ADHD Medications (vs No ADHD Medications)  SA9.2. Cohorts: CNS stimulants (vs Non-Stimulants) | 66 |
| **Treatment Pathways Analysis Report** | |  |
| SA10 | Treatment Pathways Analysis Report  SA10.1. Treatment Distribution Across Lines of Treatment in Individuals with ADHD and PTSD  SA10.2. CNS Stimulants Distribution Across Lines of Treatment by Cohort  SA10.3. Non-stimulants Distribution Across Lines of Treatment by Cohort | 68 |

**Supplement Table 1: Treatment Trends by Age and Sex**

| **Medication Class**  **Age group** | **All**  **Sex** | **ADHD without**  **PTSD^a^; n^b^(%)** | **ADHD with**  **PTSD; n^b^(%)** | **Absolute Risk Difference^c^ (%)** | **RR (95%CI)** |
| --- | --- | --- | --- | --- | --- |
| **ADHD medications** |  |  |  |  |  |
| 6-11 | All | 2,769 (57.2) | 3,311 (68.3) | +11.1 | 1.20(1.16,1.23) |
|  | Male | 1,779 (59.3) | 2,029 (67.7) | +8.4 | 1.14(1.10,1.19) |
|  | Female | 613 (53.4) | 737 (64.3) | +10.9 | 1.20(1.12,1.29) |
| 12-18 | All | 13,507 (57.9) | 14,392 (61.7) | +3.8 | 1.07(1.05,1.08) |
|  | Male | 7,049 (59.4) | 7,758 (65.4) | +6.0 | 1.10(1.08,1.12) |
|  | Female | 5,993 (56.5) | 6,255 (58.9) | +2.4 | 1.04(1.02,1.07) |
| **CNS Stimulants** |  |  |  |  |  |
| 6-11 | All | 2,316 (47.8) | 2,541 (52.4) | +4.6 | 1.10(1.05,1.14) |
|  | Male | 1,469 (49.0) | 1,574 (52.5) | +3.5 | 1.07(1.02,1.13) |
|  | Female | 512 (44.6) | 549 (47.9) | +3.3 | 1.07(0.98,1.17) |
| 12-18 | All | 11,044 (47.4) | 10,532 (45.2) | −2.2 | 0.95(0.94,0.97) |
|  | Male | 5,738 (48.4) | 5,767 (48.6) | +0.2 | 1.01(0.98,1.03) |
|  | Female | 4,885 (46.0) | 4,485 (42.3) | −3.7 | 0.92(0.89,0.95) |
| Methylphenidate |  |  |  |  |  |
| 6-11 | All | 1,829 (37.8) | 1,963 (40.5) | +2.7 | 1.07(1.02,1.13) |
|  | Male | 1,163 (38.8) | 1,204 (40.2) | +1.4 | 1.04(0.97,1.10) |
|  | Female | 394 (34.4) | 428 (37.3) | +2.9 | 1.09(0.97,1.21) |
| 12-18 | All | 7,841 (33.6) | 7,311 (31.3) | −2.3 | 0.93(0.91,0.96) |
|  | Male | 4,144 (34.9) | 4,061 (34.2) | −0.7 | 0.98(0.95,1.02) |
|  | Female | 3,389 (31.9) | 3,037 (28.6) | −3.3 | 0.90(0.86,0.93) |
| Amphetamine |  |  |  |  |  |
| 6-11 | All | 975 (20.1) | 1,067 (22,0) | +1.9 | 1.09(1.01,1.18) |
|  | Male | 620 (20.7) | 663 (22.1) | +1.4 | 1.07(0.97,1.18) |
|  | Female | 236 (20.6) | 229 (20.0) | −0.6 | 0.97(0.83,1.14) |
| 12-18 | All | 5,576 (23.9) | 5,260 (22.6) | −1.3 | 0.94(0.91,0.98) |
|  | Male | 2,920 (24.6) | 2,897 (24.4) | −0.2 | 0.99(0.95,1.04) |
|  | Female | 2,433 (22.9) | 2,237 (21.1) | −1.8 | 0.92(0.87,0.97) |
| **Non-stimulants** |  |  |  |  |  |
| 6-11 | All | 1,474 (30.4) | 2,528 (52.2) | +21.8 | 1.72(1.63,1.80) |
|  | Male | 974 (32.5) | 1,583 (52.8) | +20.3 | 1.63(1.53,1.73) |
|  | Female | 298 (26.0) | 541 (47.2) | +21.2 | 1.82(1.62,2.04) |
| 12-18 | All | 7,034 (30.2) | 10,233 (43.9) | +13.7 | 1.46(1.42,1.49) |
|  | Male | 3,960 (33.4) | 5,896 (49.7) | +16.3 | 1.49(1.44,1.54) |
|  | Female | 2,820 (26.6) | 4,118 (38.8) | +12.2 | 1.46(1.40,1.52) |
| **Alpha-2 agonists** |  |  |  |  |  |
| 6-11 | All | 1,381 (28.5) | 2,438 (50.3) | +21.8 | 1.77(1.68,1.86) |
|  | Male | 909 (30.3) | 1,535 (51.2) | +20.9 | 1.69(1.58,1.80) |
|  | Female | 285 (24.8) | 517 (45.1) | +20.3 | 1.81(1.61,2.04) |
| 12-18 | All | 6,295 (27.0) | 9,654 (41.4) | +14.4 | 1.53(1.49,1.57) |
|  | Male | 3,619 (30.5) | 5,668 (47.8) | +17.3 | 1.57(1.52,1.63) |
|  | Female | 2,433 (22.9) | 3,796 (35.8) | +12.9 | 1.56(1.49,1.63) |
| Clonidine |  |  |  |  |  |
| 6-11 | All | 625 (12.9) | 1,347 (27.8) | +14.9 | 2.16(1.98,2.35) |
|  | Male | 379 (12.6) | 846 (28.2) | +15.6 | 2.23(2.00,2.49) |
|  | Female | 129 (11.2) | 305 (26.6) | +15.4 | 2.36(1.96,2.86) |
| 12-18 | All | 2,914 (12.5) | 5,240 (22.5) | +10.0 | 1.80(1.73,1.87) |
|  | Male | 1,739 (14.7) | 3,098 (26.1) | +11.4 | 1.78(1.69,1.88) |
|  | Female | 1,113 (10.5) | 2,018 (19.0) | +8.5 | 1.81(1.69,1.94) |
| Guanfacine |  |  |  |  |  |
| 6-11 | All | 1,012 (20.9) | 1,599 (33.0) | +12.1 | 1.58(1.48,1.69) |
|  | Male | 692 (23.1) | 1,036 (34.6) | +11.5 | 1.50(1.38,1.63) |
|  | Female | 198 (17.3) | 315 (27.5) | +10.2 | 1.59(1.36,1.86) |
| 12-18 | All | 4,309 (18.5) | 6,305 (27.0) | +8.5 | 1.46(1.41,1.51) |
|  | Male | 2,452 (20.7) | 3,766 (31.7) | +11.0 | 1.54(1.47,1.61) |
|  | Female | 1,628 (15.3) | 2,417 (22.8) | +7.5 | 1.49(1.40,1.57) |
| **Atomoxetine** |  |  |  |  |  |
| 6-11 | All | 198 (4.1) | 303 (6.3) | +2.2 | 1.53(1.29,1.82) |
|  | Male | 138 (4.6) | 181 (6.0) | +1.4 | 1.31(1.06,1.63) |
|  | Female | 35 (3.1) | 71 (6.2) | +3.1 | 2.03(1.37,3.02) |
| 12-18 | All | 1,355 (5.8) | 1,625 (7.0) | +1.2 | 1.20(1.12,1.29) |
|  | Male | 699 (5.9) | 875 (7.4) | +1.5 | 1.25(1.14,1.38) |
|  | Female | 610 (5.7) | 687 (6.5) | +0.8 | 1.13(1.01,1.25) |
| **Viloxazine** |  |  |  |  |  |
| 6-11 | All | 42 (0.9) | 47 (1.0) | +0.1 | 1.12(0.74,1.69) |
|  | Male | 30 (1.0) | 26 (0.9) | −0.1 | 0.87(0.51,1.46) |
|  | Female | 10(0.9) | 10(0.9) | +0.0 | 1.00(0.42,2.39) |
| 12-18 | All | 136 (0.6) | 109 (0.5) | −0.1 | 0.80(0.62,1.03) |
|  | Male | 59 (0.5) | 64 (0.5) | +0.0 | 1.09(0.76,1.54) |
|  | Female | 51 (0.5) | 34 (0.3) | −0.2 | 0.67(0.43,1.03) |
| **Antidepressants** |  |  |  |  |  |
| 6-11 | All | 907 (18.7) | 1,537 (31.7) | +13.0 | 1.70(1.58,1.82) |
|  | Male | 525 (17.5) | 902 (30.1) | +12.6 | 1.72(1.56,1.89) |
|  | Female | 184 (16.0) | 357 (31.1) | +15.1 | 1.94(1.66,2.27) |
| 12-18 | All | 9,663 (41.4) | 11,969 (51.3) | +9.9 | 1.24(1.21,1.26) |
|  | Male | 4,132 (34.8) | 5,364 (45.2) | +10.4 | 1.30(1.26,1.34) |
|  | Female | 5,267 (49.6) | 6,211 (58.5) | +8.9 | 1.18(1.15,1.21) |
| **Antipsychotics** |  |  |  |  |  |
| 6-11 | All | 423 (8.7) | 1,158 (23.9) | +15.2 | 2.74(2.47,3.04) |
|  | Male | 317 (10.6) | 745 (24.8) | +14.2 | 2.35(2.08,2.65) |
|  | Female | 81 (7.1) | 210 (18.3) | +11.2 | 2.59(2.03,3.31) |
| 12-18 | All | 4,957 (21.3) | 7,846 (33.6) | +12.3 | 1.58(1.54,1.63) |
|  | Male | 2,612 (22.0) | 4,107 (34.6) | +12.6 | 1.57(1.52,1.64) |
|  | Female | 2,297 (21.6) | 3,585 (33.8) | +12.2 | 1.56(1.49,1.63) |
| **Mood Stabilizers** |  |  |  |  |  |
| 6-11 | All | 129 (2.7) | 204 (4.2) | +1.5 | 1.58(1.27,1.96) |
|  | Male | 87 (2.9) | 133 (4.4) | +1.5 | 1.53(1.17,1.99) |
|  | Female | 24 (2.1) | 39 (3.4) | +1.3 | 1.63(0.98,2.68) |
| 12-18 | All | 1,677 (7.2) | 2,474 (10.6) | +3.4 | 1.48(1.39,1.57) |
|  | Male | 840 (7.1) | 1,260 (10.6) | +3.5 | 1.50(1.38,1.63) |
|  | Female | 853 (8.0) | 1,174 (11.1) | +3.1 | 1.38(1.27,1.50) |
| **Psychotherapy** |  |  |  |  |  |
| 6-11 | All | 756 (15.6) | 1,451 (29.9) | +14.3 | 1.92(1.78,2.07) |
|  | Male | 467 (15.6) | 890 (29.7) | +14.1 | 1.91(1.73,2.11) |
|  | Female | 150 (13.1) | 279 (24.3) | +11.2 | 1.86(1.55,2.23) |
| 12-18 | All | 4,783 (20.5) | 7,428 (31.9) | +11.4 | 1.55(1.51,1.60) |
|  | Male | 2,302 (19.4) | 3,522 (29.7) | +10.3 | 1.53(1.46,1.60) |
|  | Female | 2,400 (22.6) | 3,383 (31.9) | +9.3 | 1.41(1.35,1.47) |

Abbreviation: ADHD: Attention-deficit/hyperactivity disorder; CI: Confidence Interval; RR: Relative Risk; PTSD: Post-traumatic stress disorder

^a^Reference group

^b^Propensity score matching was used to adjust for demographic factors and psychiatric comorbidities;

n=6-11: All 4,845; Male 2,998; Female 1,147; 12-18 All 23,321; Male 11,864; Female 10,611

^c^Absolute Risk Difference (%) represents the difference in the proportion of patients with the outcome between the “ADHD without PTSD” and “ADHD with PTSD” subgroups, expressed in percentage points.

**Supplement Table 2: Treatment Trends by Race and Ethnicity**

| **Medication Class** | **Groups** | **ADHD without**  **PTSD^a;^ n^b^(%)** | **ADHD with**  **PTSD; n^b^(%)** | **Absolute Risk Difference^c^ (%)** | **RR (95%CI)** |
| --- | --- | --- | --- | --- | --- |
| **ADHD medications** | White | 10,653 (60.3) | 11,435 (64.7) | +4.4 | 1.07(1.06,1.09) |
|  | Black | 3,076 (53.8) | 3,519 (61.5) | +7.7 | 1.14(1.11,1.18) |
|  | Non-Hispanic | 10,496 (59.7) | 11,227 (63.8) | +4.1 | 1.07(1.05,1.09) |
|  | Hispanic | 1,880 (55.9) | 2,125 (63.2) | +7.3 | 1.13(1.09,1.18) |
| **CNS Stimulants** | White | 8,694 (49.2) | 8,252 (46.7) | -2.5 | 0.95(0.93,0.97) |
|  | Black | 2,592 (45.3) | 2,745 (48.0) | +2.7 | 1.06(1.02,1.10) |
|  | Non-Hispanic | 8,679 (49.3) | 8,481 (48.2) | -1.1 | 0.98(0.96,1.00) |
|  | Hispanic | 1,461 (43.5) | 1,382 (41.1) | -2.4 | 0.95(0.90,1.00) |
| Methylphenidate | White | 6,270 (35.5) | 5,777 (32.7) | -2.8 | 0.92(0.90,0.95) |
|  | Black | 1,826 (31.9) | 1,990 (34.8) | +2.9 | 1.09(1.04,1.15) |
|  | Non-Hispanic | 6,318 (35.9) | 6,020 (34.2) | -1.7 | 0.95(0.93,0.98) |
|  | Hispanic | 1,076 (32.0) | 990 (29.4) | -2.6 | 0.92(0.86,0.99) |
| Amphetamine | White | 4,287 (24.2) | 4,107 (23.2) | -1.0 | 0.96(0.92,0.99) |
|  | Black | 1,292 (22.6) | 1,266 (22.1) | -0.5 | 0.98(0.92,1.05) |
|  | Non-Hispanic | 4,380 (24.9) | 4,215 (24.0) | -0.9 | 0.96(0.93,1.00) |
|  | Hispanic | 649 (19.3) | 640 (19.0) | -0.3 | 0.99(0.89,1.09) |
| **Non-stimulants** | White | 5,583 (31.6) | 8,331 (47.2) | +15.6 | 1.49(1.45,1.53) |
|  | Black | 1,576 (27.6) | 2,558 (44.7) | +17.1 | 1.62(1.54,1.71) |
|  | Non-Hispanic | 5,500 (31.3) | 8,121 (46.2) | +14.9 | 1.48(1.44,1.52) |
|  | Hispanic | 992 (29.5) | 1,551 (46.1) | +16.6 | 1.56(1.47,1.67) |
| **Alpha-2 agonists** | White | 5,004 (28.3) | 7,846 (44.4) | +16.1 | 1.57(1.52,1.61) |
|  | Black | 1,475 (25.8) | 2,493 (43.8) | +18.0 | 1.69(1.60,1.78) |
|  | Non-Hispanic | 5,018 (28.5) | 7,727 (43.9) | +15.4 | 1.54(1.50,1.59) |
|  | Hispanic | 905 (26.9) | 1,474 (43.8) | +16.9 | 1.63(1.52,1.74) |
| Clonidine | White | 2,292 (13.0) | 4,267 (24.2) | +11.2 | 1.86(1.78,1.95) |
|  | Black | 781 (13.7) | 1,386 (24.2) | +10.5 | 1.78(1.64,1.92) |
|  | Non-Hispanic | 2,390 (13.6) | 4,187 (23.8) | +10.2 | 1.75(1.67,1.83) |
|  | Hispanic | 442 (13.1) | 819 (24.4) | +11.3 | 1.85(1.67,2.06) |
| Guanfacine | White | 3,468 (19.6) | 5,122 (29.0) | +9.4 | 1.48(1.42,1.53) |
|  | Black | 921 (16.1) | 1,614 (28.2) | +12.1 | 1.75(1.63,1.88) |
|  | Non-Hispanic | 3,408 (19.4) | 5,100 (29.0) | +9.6 | 1.50(1.44,1.55) |
|  | Hispanic | 606 (18.0) | 956 (28.4) | +10.4 | 1.58(1.44,1.73) |
| **Atomoxetine** | White | 1,112 (6.3) | 1,366 (7.7) | +1.4 | 1.23(1.14,1.33) |
|  | Black | 185 (3.2) | 249 (4.4) | +1.2 | 1.35(1.12,1.62) |
|  | Non-Hispanic | 954 (5.4) | 1,211 (6.9) | +1.5 | 1.27(1.17,1.38) |
|  | Hispanic | 167 (5.0) | 234 (7.0) | +2.0 | 1.40(1.16,1.70) |
| **Viloxazine** | White | 130 (0.7) | 119 (0.7) | 0.0 | 0.92(0.71,1.17) |
|  | Black | 31 (0.5) | 14 (0.2) | -0.3 | 0.45(0.24,0.85) |
|  | Non-Hispanic | 120 (0.7) | 115 (0.7) | 0.0 | 0.96(0.74,1.24) |
|  | Hispanic | 17 (0.5) | 10 (0.3) | -0.2 | 0.59(0.27,1.28) |
| **Antidepressants** | White | 7,475 (42.3) | 9,256 (52.4) | +10.1 | 1.24(1.21,1.27) |
|  | Black | 1,531 (26.8) | 2,279 (39.8) | +13.0 | 1.49(1.41,1.57) |
|  | Non-Hispanic | 6,725 (38.2) | 8,620 (49.2) | +11.0 | 1.28(1.25,1.31) |
|  | Hispanic | 1,091 (32.5) | 1,390 (41.3) | +8.8 | 1.27(1.20,1.36) |
| **Antipsychotics** | White | 3,548 (20.1) | 5,942 (33.6) | +13.5 | 1.68(1.62,1.74) |
|  | Black | 1,119 (19.6) | 1,900 (33.2) | +13.6 | 1.70(1.59,1.81) |
|  | Non-Hispanic | 3,460 (19.7) | 5,786 (32.9) | +13.2 | 1.67(1.61,1.74) |
|  | Hispanic | 632 (18.8) | 925 (27.5) | +8.7 | 1.46(1.34,1.60) |
| **Mood Stabilizers** | White | 1,262 (7.1) | 1,894 (10.7) | +3.6 | 1.50(1.40,1.62) |
|  | Black | 299 (5.2) | 447 (7.8) | +2.6 | 1.50(1.30,1.72) |
|  | Non-Hispanic | 1,169 (6.6) | 1,703 (9.7) | +3.1 | 1.46(1.36,1.57) |
|  | Hispanic | 182 (5.4) | 302 (9.0) | +3.6 | 1.66(1.39,1.98) |
| **Psychotherapy** | White | 3,717 (21.0) | 5,593 (31.7) | +10.7 | 1.51(1.45,1.56) |
|  | Black | 961 (16.8) | 1,592 (27.8) | +11.0 | 1.66(1.54,1.78) |
|  | Non-Hispanic | 3,499 (19.9) | 5,371 (30.5) | +10.6 | 1.54(1.48,1.59) |
|  | Hispanic | 673 (20.0) | 972 (28.9) | +8.9 | 1.44(1.33,1.57) |

Abbreviation: ADHD: Attention-deficit/hyperactivity disorder; CI: Confidence Interval; RR: Relative Risk; PTSD: Post-traumatic stress disorder

^a^Reference group

^b^Propensity score matching was used to adjust for demographic factors and psychiatric comorbidities;

n=White17,668; Black 5,720; Non-Hispanic17,593; Hispanic 3,362

^c^Absolute Risk Difference (%) represents the difference in the proportion of patients with the outcome between the “ADHD without PTSD” and “ADHD with PTSD” subgroups, expressed in percentage point

### Supplement Table 3: Sensitivity Analysis – Association of Sustained ADHD Medication Exposure on Acute Clinical Outcomes in Individuals with ADHD and PTSD

| Medication TypeOutcomes | aHR (95% CI) | p-value | FDR-adjusted p-value |
| --- | --- | --- | --- |
| CNS stimulants (vs. Non-stimulants^a^) |  |  |  |
| Inpatient hospitalization | 0.60 (0.55, 0.66) | < 0.001 | 0.001 |
| Emergency visit | 0.63 (0.57, 0.70) | < 0.001 | 0.001 |
| Antipsychotics | 0.48 (0.44, 0.52) | < 0.001 | 0.001 |
| Mood Stabilizers^b^ | 0.52 (0.45, 0.60) | < 0.001 | 0.001 |

aHR = adjusted hazard ratio; SE = standard error; CI = confidence interval.

All models were adjusted for socio-demographic (age, sex, race/ethnicity) and comorbidity.

FDR-adjusted p-values were calculated using the Benjamini-Hochberg method to control for multiple comparisons.

^a^Reference group

^b^Mood stabilizers: lithium, valproate, oxcarbazepine, carbamazepine, and lamotrigine.

**** ****

**Appendix**

**Diagnosis code**

| F10-19 | Mental and behavioral disorders due to psychoactive substance use |
| --- | --- |
| F20-29 | Schizophrenia, schizotypal, delusional, and other non-mood psychotic disorders |
| F30-39 | Mood [affective] disorders |
| F31 | Bipolar disorder |
| F32-3 | Depressive episode (including MDD) |
| F41.9 | Anxiety disorder, unspecified |
| F41.1 | Generalized anxiety disorder |
| F41.0 | Panic disorder |
| F42 | Obsessive-compulsive disorder |
| F43.1 | Post-traumatic stress disorder |
| F50 | Eating Disorders |
| F51 | Sleep disorders not due to a substance or known physiological condition |
| F60.3 | Borderline personality disorder |
| F70-79 | Intellectual Disabilities |
| F84 | Autism spectrum disorder |
| F90 | Attention-deficit hyperactivity disorders |
| F91 | Disruptive behavior disorders |
| F95 | Tic disorder |

**Procedure Code**

| 99201-5, 99211-5 | Outpatient Services |
| --- | --- |
| 99281-5 | Emergency Department Services |
| 99217-6,99231-6,99238-39 | Hospital Inpatient/ Observation Services |
| 90791-2,90832-8, 90845-7, 90849, 90853 | Psychiatric Services and Procedures |
| 90832-34, 36-40, 90846-7, 90849, 90853, 90785, 90882, 90885, 90887 | Psychotherapy Services and Procedures |

**Medication Code**

|  |  | VA Drug Classification System | ATC classification system (RxNorm) |
| --- | --- | --- | --- |
| CNS Stimulants | Amphetamines | CN 801 |  |
|  | Mixed Amphetamine Salts |  | 725 |
|  | Lisdexamfetamine |  | 700810 |
|  | Amphetamine-like Stimulants | CN802 |  |
|  | Methylphenidate |  | 6901 |
|  | Dexmethylphenidate |  | 352372 |
| Non-stimulants | Atomoxetine |  | 38400 |
|  | Bupropion |  | 42347 |
|  | Viloxazine |  | 11193 |
|  | Clonidine |  | 2599 |
|  | Guanfacine |  | 40114 |
| Antidepressants |  | CN600 |  |
| Antipsychotics |  | CN700 |  |
| Mood Stabilizers | Lithium Salts | CN750 |  |
|  | Oxcarbazepine |  | 32624 |
|  | Lamotrigine |  | 28439 |
|  | Valproate |  | 40254 |
|  | Carbamazepine |  | 2002 |

ATC: Anatomical Therapeutic Chemical, VA: Veterans Affairs

**Supplement Analysis 1.**

**Summary Statistics for ADHD Cohort in TriNetX Database (N = 714,129)**

**
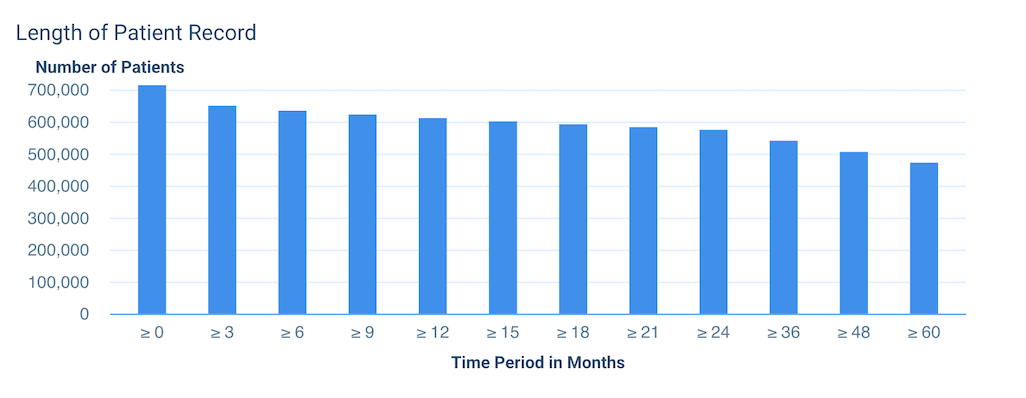
**

**
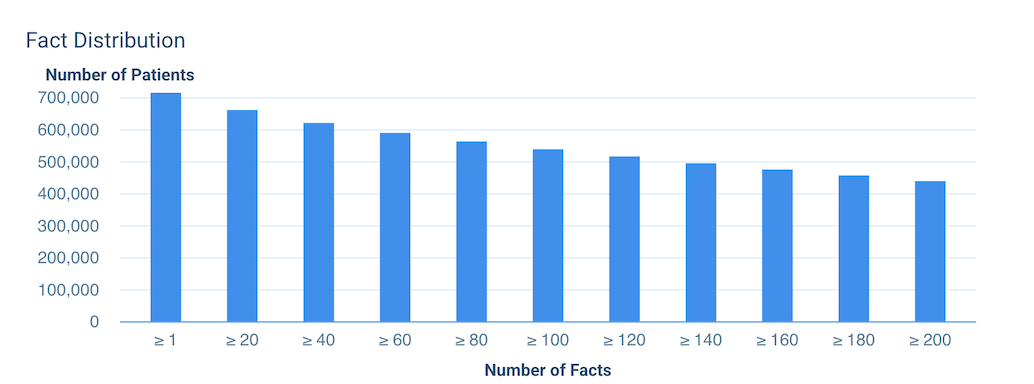
**

**
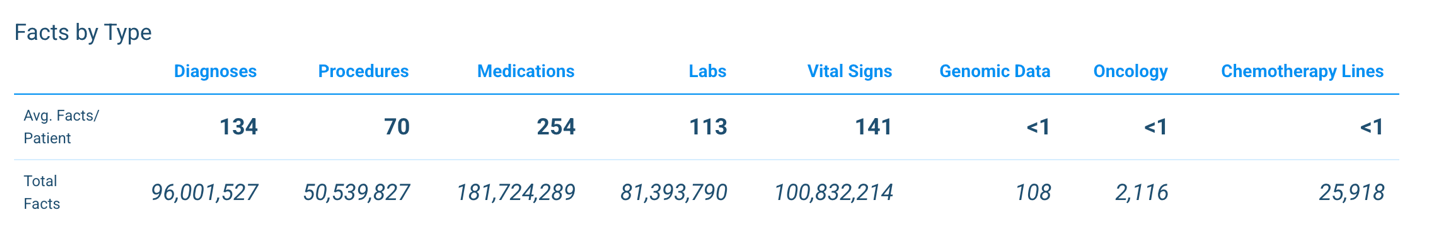
**

**
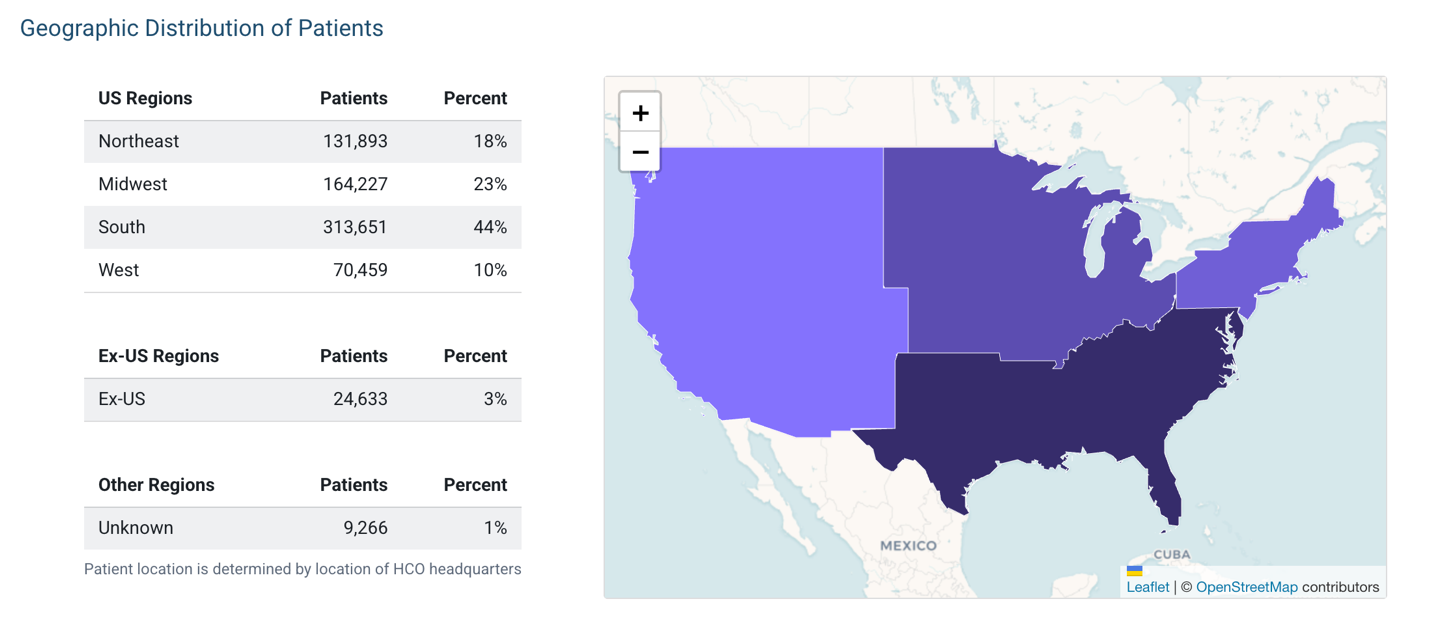
**

**Compare Outcomes Analysis**

**Supplement Analysis 2.**  **ADHD Treatment Patterns in Patients with Subsequent PTSD Diagnosis**

**SA2.1. Cohorts: ADHD Cohort with PTSD (vs. ADHD Cohort without PTSD)**

### Query Criteria for Cohort 1 ((query name: ADHD+PTSD)

This query was run on the network Research with 101 HCO(s) queried and 101 HCO(s) responded. A total of 68 provider(s) responded with patients. The final cohort included 30,373 patients who matched the query criteria listed in the table below.

| Cohort 1 | | | | | |
| --- | --- | --- | --- | --- | --- |
|  | must have |  | demographics | Age | Age (between 6 and 18 years (most recent occurrence)) |
|  |  | and | diagnosis | UMLS:ICD10CM:F90 | Attention-deficit hyperactivity disorders |
|  |  | and | diagnosis | UMLS:ICD10CM:F43.1 | Post-traumatic stress disorder (PTSD) |

### Query Criteria for Cohort 2 (query name: ADHD)

This query was run on the network Research with 101 HCO(s) queried and 101 HCO(s) responded. A total of 93 provider(s) responded with patients. The final cohort included 684,202 patients who matched the query criteria listed in the table below.

| Cohort 2 | | | | | |
| --- | --- | --- | --- | --- | --- |
|  | must have |  | demographics | Age | Age (between 6 and 18 years (most recent occurrence)) |
|  |  | and | diagnosis | UMLS:ICD10CM:F90 | Attention-deficit hyperactivity disorders |
|  | cannot have |  | diagnosis | UMLS:ICD10CM:F43.1 | Post-traumatic stress disorder (PTSD) |

### Outcome Definitions

Table below outlines the definitions for each outcome and the analysis specifications. For outcome definitions consisting of more than one term, at least one term must match.

| ADHD medications | | | | |
| --- | --- | --- | --- | --- |
|  | **Outcome definition** | | | |
|  | | Medication | NLM:VA:CN801 | AMPHETAMINES |
|  | | Medication | NLM:VA:CN802 | AMPHETAMINE LIKE STIMULANTS |
|  | | Medication | NLM:RXNORM:2599 | clonidine |
|  | | Medication | NLM:RXNORM:40114 | guanfacine |
|  | | Medication | NLM:RXNORM:38400 | atomoxetine |
|  | | Medication | NLM:RXNORM:11196 | viloxazine |
|  | **Settings for the performed analyses** | | | |
|  | | Risk analysis | | including patients with outcome prior to the time window |
| CNS Stimulants | | | | |
|  | **Outcome definition** | | | |
|  | | Medication | NLM:VA:CN802 | AMPHETAMINE LIKE STIMULANTS |
|  | | Medication | NLM:VA:CN801 | AMPHETAMINES |
|  | **Settings for the performed analyses** | | | |
|  | | Risk analysis | | including patients with outcome prior to the time window |
| Methylphenidate | | | | |
|  | **Outcome definition** | | | |
|  | | Medication | NLM:VA:CN802 | AMPHETAMINE LIKE STIMULANTS |
|  | **Settings for the performed analyses** | | | |
|  | | Risk analysis | | including patients with outcome prior to the time window |
| Amphetamine | | | | |
|  | **Outcome definition** | | | |
|  | | Medication | NLM:VA:CN801 | AMPHETAMINES |
|  | **Settings for the performed analyses** | | | |
|  | | Risk analysis | | including patients with outcome prior to the time window |
| Non-stimulants | | | | |
|  | **Outcome definition** | | | |
|  | | Medication | NLM:RXNORM:2599 | clonidine |
|  | | Medication | NLM:RXNORM:40114 | guanfacine |
|  | | Medication | NLM:RXNORM:38400 | atomoxetine |
|  | | Medication | NLM:RXNORM:11196 | viloxazine |
|  | **Settings for the performed analyses** | | | |
|  | | Risk analysis | | including patients with outcome prior to the time window |
| Alpha-2 agonists | | | | |
|  | **Outcome definition** | | | |
|  | | Medication | NLM:RXNORM:40114 | guanfacine |
|  | | Medication | NLM:RXNORM:2599 | clonidine |
|  | **Settings for the performed analyses** | | | |
|  | | Risk analysis | | including patients with outcome prior to the time window |
| Clonidine | | | | |
|  | **Outcome definition** | | | |
|  | | Medication | NLM:RXNORM:2599 | clonidine |
|  | **Settings for the performed analyses** | | | |
|  | | Risk analysis | | including patients with outcome prior to the time window |
| Guanfacine | | | | |
|  | **Outcome definition** | | | |
|  | | Medication | NLM:RXNORM:40114 | guanfacine |
|  | **Settings for the performed analyses** | | | |
|  | | Risk analysis | | including patients with outcome prior to the time window |
| Atomoxetine | | | | |
|  | **Outcome definition** | | | |
|  | | Medication | NLM:RXNORM:38400 | atomoxetine |
|  | **Settings for the performed analyses** | | | |
|  | | Risk analysis | | including patients with outcome prior to the time window |
| Viloxazine | | | | |
|  | **Outcome definition** | | | |
|  | | Medication | NLM:RXNORM:11196 | viloxazine |
|  | **Settings for the performed analyses** | | | |
|  | | Risk analysis | | including patients with outcome prior to the time window |
| Antidepressants | | | | |
|  | **Outcome definition** | | | |
|  | | Medication | NLM:VA:CN600 | ANTIDEPRESSANTS |
|  | **Settings for the performed analyses** | | | |
|  | | Risk analysis | | including patients with outcome prior to the time window |
| Antipsychotics | | | | |
|  | **Outcome definition** | | | |
|  | | Medication | NLM:VA:CN700 | ANTIPSYCHOTICS |
|  | **Settings for the performed analyses** | | | |
|  | | Risk analysis | | including patients with outcome prior to the time window |
| Mood Stabilizers | | | | |
|  | **Outcome definition** | | | |
|  | | Medication | NLM:VA:CN750 | LITHIUM SALTS |
|  | | Medication | NLM:RXNORM:2002 | carbamazepine |
|  | | Procedure | UMLS:CPT:80183 | Oxcarbazepine |
|  | | Medication | NLM:RXNORM:40254 | valproate |
|  | | Medication | NLM:RXNORM:28439 | lamotrigine |
|  | **Settings for the performed analyses** | | | |
|  | | Risk analysis | | including patients with outcome prior to the time window |
| Psychotherapy | | | | |
|  | **Outcome definition** | | | |
|  | | Procedure | UMLS:CPT:1021137 | Psychotherapy Services and Procedures |
|  | **Settings for the performed analyses** | | | |
|  | | Risk analysis | | including patients with outcome prior to the time window |

### Time Window Used in this Analysis

This analysis included outcomes that occurred in the time window that started on the same day as the first occurrence of the index event. Since no end date was specified, all outcomes after the first occurrence of the index event were included.

Propensity Score Matching

| **Cohort 1 (N = 29,471) and cohort 2 (N = 29,471) characteristics after propensity score matching** | | | | | | | | | |
| --- | --- | --- | --- | --- | --- | --- | --- | --- | --- |
|  | **Demographics** | | | | | | | | |
|  |  | Cohort | |  | Mean ± SD | Patients | % of Cohort | P-Value | Std diff. |
|  |  | 1 2 | AI | Age at Index | 10.8 +/- 3.4 10.8 +/- 3.5 | 29,471 29,471 | 100% 100% | 0.394 | 0.007 |
|  |  | 1 2 | 2186-5 | Not Hispanic or Latino |  | 20,999 21,027 | 71.3% 71.3% | 0.799 | 0.002 |
|  |  | 1 2 | 2054-5 | Black or African American |  | 5,825 5,680 | 19.8% 19.3% | 0.132 | 0.012 |
|  |  | 1 2 | M | Male |  | 15,419 15,367 | 52.3% 52.1% | 0.668 | 0.004 |
|  | **Diagnosis** | | | | | | | | |
|  |  | Cohort | |  | Mean ± SD | Patients | % of Cohort | P-Value | Std diff. |
|  |  | 1 2 | F41.9 | Anxiety disorder, unspecified |  | 10,285 10,400 | 34.9% 35.3% | 0.321 | 0.008 |
|  |  | 1 2 | F41.1 | Generalized anxiety disorder |  | 5,343 5,493 | 18.1% 18.6% | 0.111 | 0.013 |
|  |  | 1 2 | F30-F39 | Mood [affective] disorders |  | 14,342 14,444 | 48.7% 49.0% | 0.401 | 0.007 |
|  |  | 1 2 | F91 | Conduct disorders |  | 9,653 9,740 | 32.8% 33.0% | 0.446 | 0.006 |
|  |  | 1 2 | F42 | Obsessive-compulsive disorder |  | 1,113 1,145 | 3.8% 3.9% | 0.492 | 0.006 |
|  |  | 1 2 | F84 | Pervasive developmental disorders |  | 3,171 3,154 | 10.8% 10.7% | 0.821 | 0.002 |
|  |  | 1 2 | F51 | Sleep disorders not due to a substance or known physiological condition |  | 1,728 1,736 | 5.9% 5.9% | 0.889 | 0.001 |
|  |  | 1 2 | F50 | Eating disorders |  | 1,139 1,069 | 3.9% 3.6% | 0.129 | 0.013 |
|  |  | 1 2 | F10-F19 | Mental and behavioral disorders due to psychoactive substance use |  | 1,834 1,761 | 6.2% 6.0% | 0.209 | 0.010 |
|  |  | 1 2 | F20-F29 | Schizophrenia, schizotypal, delusional, and other non-mood psychotic disorders |  | 778 689 | 2.6% 2.3% | 0.019 | 0.019 |
|  |  | 1 2 | F70-F79 | Intellectual Disabilities |  | 829 742 | 2.8% 2.5% | 0.026 | 0.018 |
|  |  | 1 2 | F41.0 | Panic disorder [episodic paroxysmal anxiety] |  | 1,070 991 | 3.6% 3.4% | 0.076 | 0.015 |
|  |  | 1 2 | F32 | Depressive episode |  | 9,101 8,857 | 30.9% 30.1% | 0.029 | 0.018 |
|  |  | 1 2 | F33 | Major depressive disorder, recurrent |  | 3,888 3,914 | 13.2% 13.3% | 0.752 | 0.003 |
|  |  | 1 2 | F34.81 | Disruptive mood dysregulation disorder |  | 2,986 2,927 | 10.1% 9.9% | 0.419 | 0.007 |
|  |  | 1 2 | F31 | Bipolar disorder |  | 1,349 1,258 | 4.6% 4.3% | 0.068 | 0.015 |
|  |  | 1 2 | F60.3 | Borderline personality disorder |  | 642 566 | 2.2% 1.9% | 0.027 | 0.018 |
|  |  | 1 2 | F95 | Tic disorder |  | 738 669 | 2.5% 2.3% | 0.063 | 0.015 |

| **Follow-up Time (After Matching)** | | | | | |
| --- | --- | --- | --- | --- | --- |
| Cohort | Mean Follow-up (Days) | Standard Deviation | Median Follow-up (Days) | Interquartile Range |  |
| ADHD+PTSD | 856.591 | 898.228 | 562 | 1154 |  |
| ADHD | 863.059 | 945.638 | 536 | 1211 |  |

**SA2.2. Cohorts: Male ADHD Cohort with PTSD (vs. Male ADHD Cohort without PTSD)**

### Query Criteria for Cohort 1 (query name: ADHD+PTSD_M)

This query was run on the network Research with 100 HCO(s) queried and 100 HCO(s) responded. A total of 68 provider(s) responded with patients. The final cohort included 15,670 patients who matched the query criteria listed in the table below.

| Cohort 1 | | | | | |
| --- | --- | --- | --- | --- | --- |
|  | must have |  | demographics | Age | Age (between 6 and 18 years (most recent occurrence)) |
|  |  | and | demographics | UMLS:HL7V3.0:Gender:M | Male |
|  |  | and | diagnosis | UMLS:ICD10CM:F90 | Attention-deficit hyperactivity disorders |
|  |  | and | diagnosis | UMLS:ICD10CM:F43.1 | Post-traumatic stress disorder (PTSD) |

### Query Criteria for Cohort 2 (query name: ADHD_M)

This query was run on the network Research with 100 HCO(s) queried and 100 HCO(s) responded. A total of 91 provider(s) responded with patients. The final cohort included 465,048 patients who matched the query criteria listed in the table below.

| Cohort 2 | | | | | |
| --- | --- | --- | --- | --- | --- |
|  | must have |  | demographics | Age | Age (between 6 and 18 years (most recent occurrence)) |
|  |  | and | demographics | UMLS:HL7V3.0:Gender:M | Male |
|  |  | and | diagnosis | UMLS:ICD10CM:F90 | Attention-deficit hyperactivity disorders |
|  | cannot have |  | diagnosis | UMLS:ICD10CM:F43.1 | Post-traumatic stress disorder (PTSD) |

Propensity Score Matching

| **Cohort 1 (N = 15,200) and cohort 2 (N = 15,200) characteristics after propensity score matching** | | | | | | | | | |
| --- | --- | --- | --- | --- | --- | --- | --- | --- | --- |
|  | **Demographics** | | | | | | | | |
|  |  | Cohort | |  | Mean ± SD | Patients | % of Cohort | P-Value | Std diff. |
|  |  | 1 2 | AI | Age at Index | 10.0 +/- 3.3 10.0 +/- 3.4 | 15,200 15,200 | 100% 100% | 0.262 | 0.013 |
|  |  | 1 2 | 2186-5 | Not Hispanic or Latino |  | 10,967 10,945 | 72.2% 72.0% | 0.779 | 0.003 |
|  |  | 1 2 | 2054-5 | Black or African American |  | 3,246 3,143 | 21.4% 20.7% | 0.147 | 0.017 |
|  |  | 1 2 | M | Male |  | 15,200 15,200 | 100% 100% | -- | -- |
|  | **Diagnosis** | | | | | | | | |
|  |  | Cohort | |  | Mean ± SD | Patients | % of Cohort | P-Value | Std diff. |
|  |  | 1 2 | F41.9 | Anxiety disorder, unspecified |  | 4,493 4,622 | 29.6% 30.4% | 0.106 | 0.019 |
|  |  | 1 2 | F41.1 | Generalized anxiety disorder |  | 1,878 1,943 | 12.4% 12.8% | 0.261 | 0.013 |
|  |  | 1 2 | F30-F39 | Mood [affective] disorders |  | 6,187 6,231 | 40.7% 41.0% | 0.608 | 0.006 |
|  |  | 1 2 | F91 | Conduct disorders |  | 6,014 6,002 | 39.6% 39.5% | 0.888 | 0.002 |
|  |  | 1 2 | F42 | Obsessive-compulsive disorder |  | 470 438 | 3.1% 2.9% | 0.281 | 0.012 |
|  |  | 1 2 | F84 | Pervasive developmental disorders |  | 2,093 2,072 | 13.8% 13.6% | 0.726 | 0.004 |
|  |  | 1 2 | F51 | Sleep disorders not due to a substance or known physiological condition |  | 837 799 | 5.5% 5.3% | 0.334 | 0.011 |
|  |  | 1 2 | F50 | Eating disorders |  | 264 253 | 1.7% 1.7% | 0.626 | 0.006 |
|  |  | 1 2 | F10-F19 | Mental and behavioral disorders due to psychoactive substance use |  | 784 794 | 5.2% 5.2% | 0.796 | 0.003 |
|  |  | 1 2 | F20-F29 | Schizophrenia, schizotypal, delusional, and other non-mood psychotic disorders |  | 373 353 | 2.5% 2.3% | 0.452 | 0.009 |
|  |  | 1 2 | F70-F79 | Intellectual Disabilities |  | 493 445 | 3.2% 2.9% | 0.111 | 0.018 |
|  |  | 1 2 | F41.0 | Panic disorder [episodic paroxysmal anxiety] |  | 256 252 | 1.7% 1.7% | 0.858 | 0.002 |
|  |  | 1 2 | F32 | Depressive episode |  | 3,388 3,321 | 22.3% 21.8% | 0.354 | 0.011 |
|  |  | 1 2 | F33 | Major depressive disorder, recurrent |  | 1,060 1,069 | 7.0% 7.0% | 0.840 | 0.002 |
|  |  | 1 2 | F34.81 | Disruptive mood dysregulation disorder |  | 1,794 1,749 | 11.8% 11.5% | 0.421 | 0.009 |
|  |  | 1 2 | F31 | Bipolar disorder |  | 557 516 | 3.7% 3.4% | 0.203 | 0.015 |
|  |  | 1 2 | F60.3 | Borderline personality disorder |  | 302 252 | 2.0% 1.7% | 0.032 | 0.025 |
|  |  | 1 2 | F95 | Tic disorder |  | 421 371 | 2.8% 2.4% | 0.072 | 0.021 |

**SA2.3. Cohorts: Female ADHD Cohort with PTSD (vs. Female ADHD Cohort without PTSD)**

### Query Criteria for Cohort 1 (query name: ADHD+PTSD_F)

This query was run on the network Research with 100 HCO(s) queried and 100 HCO(s) responded. A total of 67 provider(s) responded with patients. The final cohort included 13,997 patients who matched the query criteria listed in the table below.

| Cohort 1 | | | | | |
| --- | --- | --- | --- | --- | --- |
|  | must have |  | demographics | Age | Age (between 6 and 18 years (most recent occurrence)) |
|  |  | and | demographics | UMLS:HL7V3.0:Gender:F | Female |
|  |  | and | diagnosis | UMLS:ICD10CM:F90 | Attention-deficit hyperactivity disorders |
|  |  | and | diagnosis | UMLS:ICD10CM:F43.1 | Post-traumatic stress disorder (PTSD) |

### Query Criteria for Cohort 2 (query name: ADHD_F)

This query was run on the network Research with 100 HCO(s) queried and 100 HCO(s) responded. A total of 90 provider(s) responded with patients. The final cohort included 209,608 patients who matched the query criteria listed in the table below.

| Cohort 2 | | | | | |
| --- | --- | --- | --- | --- | --- |
|  | must have |  | demographics | Age | Age (between 6 and 18 years (most recent occurrence)) |
|  |  | and | demographics | UMLS:HL7V3.0:Gender:F | Female |
|  |  | and | diagnosis | UMLS:ICD10CM:F90 | Attention-deficit hyperactivity disorders |
|  | cannot have |  | diagnosis | UMLS:ICD10CM:F43.1 | Post-traumatic stress disorder (PTSD) |

Propensity Score Matching

| **Cohort 1 (N = 12,389) and cohort 2 (N = 12,389) characteristics after propensity score matching** | | | | | | | | | |
| --- | --- | --- | --- | --- | --- | --- | --- | --- | --- |
|  | **Demographics** | | | | | | | | |
|  |  | Cohort | |  | Mean ± SD | Patients | % of Cohort | P-Value | Std diff. |
|  |  | 1 2 | AI | Age at Index | 11.6 +/- 3.3 11.6 +/- 3.4 | 12,389 12,389 | 100% 100% | 0.777 | 0.004 |
|  |  | 1 2 | 2186-5 | Not Hispanic or Latino |  | 9,518 9,541 | 76.8% 77.0% | 0.729 | 0.004 |
|  |  | 1 2 | 2054-5 | Black or African American |  | 2,281 2,251 | 18.4% 18.2% | 0.622 | 0.006 |
|  |  | 1 2 | M | Male |  | 0 0 | 0% 0% | -- | -- |
|  | **Diagnosis** | | | | | | | | |
|  |  | Cohort | |  | Mean ± SD | Patients | % of Cohort | P-Value | Std diff. |
|  |  | 1 2 | F41.9 | Anxiety disorder, unspecified |  | 5,292 5,330 | 42.7% 43.0% | 0.626 | 0.006 |
|  |  | 1 2 | F41.1 | Generalized anxiety disorder |  | 2,940 3,024 | 23.7% 24.4% | 0.212 | 0.016 |
|  |  | 1 2 | F30-F39 | Mood [affective] disorders |  | 7,172 7,215 | 57.9% 58.2% | 0.580 | 0.007 |
|  |  | 1 2 | F91 | Conduct disorders |  | 3,218 3,287 | 26.0% 26.5% | 0.319 | 0.013 |
|  |  | 1 2 | F42 | Obsessive-compulsive disorder |  | 549 540 | 4.4% 4.4% | 0.780 | 0.004 |
|  |  | 1 2 | F84 | Pervasive developmental disorders |  | 916 868 | 7.4% 7.0% | 0.238 | 0.015 |
|  |  | 1 2 | F51 | Sleep disorders not due to a substance or known physiological condition |  | 796 807 | 6.4% 6.5% | 0.776 | 0.004 |
|  |  | 1 2 | F50 | Eating disorders |  | 748 737 | 6.0% 5.9% | 0.768 | 0.004 |
|  |  | 1 2 | F10-F19 | Mental and behavioral disorders due to psychoactive substance use |  | 913 890 | 7.4% 7.2% | 0.574 | 0.007 |
|  |  | 1 2 | F20-F29 | Schizophrenia, schizotypal, delusional, and other non-mood psychotic disorders |  | 330 304 | 2.7% 2.5% | 0.296 | 0.013 |
|  |  | 1 2 | F70-F79 | Intellectual Disabilities |  | 305 274 | 2.5% 2.2% | 0.192 | 0.017 |
|  |  | 1 2 | F41.0 | Panic disorder [episodic paroxysmal anxiety] |  | 684 624 | 5.5% 5.0% | 0.088 | 0.022 |
|  |  | 1 2 | F32 | Depressive episode |  | 5,058 4,972 | 40.8% 40.1% | 0.266 | 0.014 |
|  |  | 1 2 | F33 | Major depressive disorder, recurrent |  | 2,384 2,399 | 19.2% 19.4% | 0.809 | 0.003 |
|  |  | 1 2 | F34.81 | Disruptive mood dysregulation disorder |  | 1,003 977 | 8.1% 7.9% | 0.542 | 0.008 |
|  |  | 1 2 | F31 | Bipolar disorder |  | 711 616 | 5.7% 5.0% | 0.007 | 0.034 |
|  |  | 1 2 | F60.3 | Borderline personality disorder |  | 274 252 | 2.2% 2.0% | 0.332 | 0.012 |
|  |  | 1 2 | F95 | Tic disorder |  | 276 242 | 2.2% 2.0% | 0.131 | 0.019 |

**Supplement Analysis 3. ADHD Prescription Patterns in Patients with Subsequent PTSD Diagnosis (Sensitivity Analysis _** **Excluding youth with documented sleep disorders)**

**Cohorts: ADHD Cohort with PTSD (vs ADHD Cohort without PTSD)**

### Query Criteria for Cohort 1 (query name: ADHD+PTSD_Nosleep)

### This query was run on the network Research with 101 HCO(s) queried and 101 HCO(s) responded. A total of 68 provider(s) responded with patients. The final cohort included 29,738 patients who matched the query criteria listed in the table below.

| Cohort 1 | | | | | |
| --- | --- | --- | --- | --- | --- |
|  | must have |  | demographics | Age | Age (between 6 and 18 years (most recent occurrence)) |
|  |  | and | diagnosis | UMLS:ICD10CM:F90 | Attention-deficit hyperactivity disorders |
|  |  | and | diagnosis | UMLS:ICD10CM:F43.1 | Post-traumatic stress disorder (PTSD) |
|  | cannot have |  | diagnosis | UMLS:ICD10CM:F51 | Sleep disorders not due to a substance or known physiological condition |

### Query Criteria for Cohort 2 (query name: ADHD_Nosleep)

This query was run on the network Research with 101 HCO(s) queried and 101 HCO(s) responded. A total of 92 provider(s) responded with patients. The final cohort included 735,246 patients who matched the query criteria listed in the table below.

| Cohort 2 | | | | | |
| --- | --- | --- | --- | --- | --- |
|  | must have |  | demographics | Age | Age (between 6 and 18 years (most recent occurrence)) |
|  |  | and | diagnosis | UMLS:ICD10CM:F90 | Attention-deficit hyperactivity disorders |
|  | cannot have |  | diagnosis | UMLS:ICD10CM:F51 | Sleep disorders not due to a substance or known physiological condition |

Propensity Score Matching

| **Cohort 1 (N = 29,628) and cohort 2 (N = 29,628) characteristics after propensity score matching** | | | | | | | | | |
| --- | --- | --- | --- | --- | --- | --- | --- | --- | --- |
|  | **Demographics** | | | | | | | | |
|  |  | Cohort | |  | Mean ± SD | Patients | % of Cohort | P-Value | Std diff. |
|  |  | 1 2 | AI | Age at Index | 10.9 +/- 3.4 10.9 +/- 3.4 | 29,628 29,628 | 100% 100% | 0.634 | 0.004 |
|  |  | 1 2 | 2186-5 | Not Hispanic or Latino |  | 19,579 19,508 | 66.1% 65.8% | 0.538 | 0.005 |
|  |  | 1 2 | 2054-5 | Black or African American |  | 6,102 6,045 | 20.6% 20.4% | 0.562 | 0.005 |
|  |  | 1 2 | M | Male |  | 15,314 15,118 | 51.7% 51.0% | 0.107 | 0.013 |
|  | **Diagnosis** | | | | | | | | |
|  |  | Cohort | |  | Mean ± SD | Patients | % of Cohort | P-Value | Std diff. |
|  |  | 1 2 | F41.9 | Anxiety disorder, unspecified |  | 10,271 10,257 | 34.7% 34.6% | 0.904 | 0.001 |
|  |  | 1 2 | F41.1 | Generalized anxiety disorder |  | 5,387 5,404 | 18.2% 18.2% | 0.856 | 0.001 |
|  |  | 1 2 | F30-F39 | Mood [affective] disorders |  | 15,274 15,349 | 51.6% 51.8% | 0.538 | 0.005 |
|  |  | 1 2 | F91 | Conduct disorders |  | 9,674 9,649 | 32.7% 32.6% | 0.827 | 0.002 |
|  |  | 1 2 | F42 | Obsessive-compulsive disorder |  | 1,151 1,128 | 3.9% 3.8% | 0.623 | 0.004 |
|  |  | 1 2 | F84 | Pervasive developmental disorders |  | 3,113 2,991 | 10.5% 10.1% | 0.099 | 0.014 |
|  |  | 1 2 | F51 | Sleep disorders not due to a substance or known physiological condition |  | 0 0 | 0% 0% | -- | -- |
|  |  | 1 2 | F50 | Eating disorders |  | 1,160 1,140 | 3.9% 3.8% | 0.671 | 0.003 |
|  |  | 1 2 | F10-F19 | Mental and behavioral disorders due to psychoactive substance use |  | 2,243 2,268 | 7.6% 7.7% | 0.699 | 0.003 |
|  |  | 1 2 | F20-F29 | Schizophrenia, schizotypal, delusional, and other non-mood psychotic disorders |  | 978 951 | 3.3% 3.2% | 0.532 | 0.005 |
|  |  | 1 2 | F70-F79 | Intellectual Disabilities |  | 828 767 | 2.8% 2.6% | 0.122 | 0.013 |
|  |  | 1 2 | F41.0 | Panic disorder [episodic paroxysmal anxiety] |  | 1,137 1,088 | 3.8% 3.7% | 0.290 | 0.009 |
|  |  | 1 2 | F32 | Depressive episode |  | 9,704 9,627 | 32.8% 32.5% | 0.500 | 0.006 |
|  |  | 1 2 | F33 | Major depressive disorder, recurrent |  | 4,396 4,457 | 14.8% 15.0% | 0.482 | 0.006 |
|  |  | 1 2 | F34.81 | Disruptive mood dysregulation disorder |  | 3,601 3,547 | 12.2% 12.0% | 0.496 | 0.006 |
|  |  | 1 2 | F31 | Bipolar disorder |  | 1,680 1,583 | 5.7% 5.3% | 0.081 | 0.014 |
|  |  | 1 2 | F60.3 | Borderline personality disorder |  | 719 688 | 2.4% 2.3% | 0.403 | 0.007 |
|  |  | 1 2 | F95 | Tic disorder |  | 682 592 | 2.3% 2.0% | 0.011 | 0.021 |

**Supplement Analysis 4. New ADHD Prescription Patterns in Patients with PTSD Diagnosis (Sensitivity Analysis)**

**Cohorts: ADHD Cohort with PTSD (vs ADHD Cohort without PTSD)**

### Query Criteria for Cohort 1 (query name: ADHD+PTSD)

This query was run on the network Research with 101 HCO(s) queried and 101 HCO(s) responded. A total of 68 provider(s) responded with patients. The final cohort included 30,373 patients who matched the query criteria listed in the table below.

| Cohort | | | | | |
| --- | --- | --- | --- | --- | --- |
|  | must have |  | demographics | Age | Age (between 6 and 18 years (most recent occurrence)) |
|  |  | and | diagnosis | UMLS:ICD10CM:F90 | Attention-deficit hyperactivity disorders |
|  |  | and | diagnosis | UMLS:ICD10CM:F43.1 | Post-traumatic stress disorder (PTSD) |

### Query Criteria for Cohort 2 (query name: ADHD)

### This query was run on the network Research with 101 HCO(s) queried and 101 HCO(s) responded. A total of 93 provider(s) responded with patients. The final cohort included 684,202 patients who matched the query criteria listed in the table below.

| Cohort | | | | | |
| --- | --- | --- | --- | --- | --- |
|  | must have |  | demographics | Age | Age (between 6 and 18 years (most recent occurrence)) |
|  |  | and | diagnosis | UMLS:ICD10CM:F90 | Attention-deficit hyperactivity disorders |
|  | cannot have |  | diagnosis | UMLS:ICD10CM:F43.1 | Post-traumatic stress disorder (PTSD) |

Outcome Definitions

|  | Settings for the performed analyses | | |
| --- | --- | --- | --- |
|  | | Risk analysis | excluding patients with outcome prior to the time window |

Propensity Score Matching

| **Cohort 1 (N = 27,570) and cohort 2 (N = 27,570) characteristics after propensity score matching** | | | | | | | | | |
| --- | --- | --- | --- | --- | --- | --- | --- | --- | --- |
|  | **Demographics** | | | | | | | | |
|  |  | Cohort | |  | Mean ± SD | Patients | % of Cohort | P-Value | Std diff. |
|  |  | 1 2 | AI | Age at Index | 10.8 +/- 3.4 10.8 +/- 3.5 | 27,570 27,570 | 100% 100% | 0.719 | 0.003 |
|  |  | 1 2 | 2186-5 | Not Hispanic or Latino |  | 19,390 19,400 | 70.3% 70.4% | 0.926 | 0.001 |
|  |  | 1 2 | 2054-5 | Black or African American |  | 5,241 5,131 | 19.0% 18.6% | 0.231 | 0.010 |
|  |  | 1 2 | M | Male |  | 14,404 14,355 | 52.2% 52.1% | 0.676 | 0.004 |
|  | **Diagnosis** | | | | | | | | |
|  |  | Cohort | |  | Mean ± SD | Patients | % of Cohort | P-Value | Std diff. |
|  |  | 1 2 | F41.9 | Anxiety disorder, unspecified |  | 9,678 9,772 | 35.1% 35.4% | 0.402 | 0.007 |
|  |  | 1 2 | F41.1 | Generalized anxiety disorder |  | 4,955 5,135 | 18.0% 18.6% | 0.047 | 0.017 |
|  |  | 1 2 | F30-F39 | Mood [affective] disorders |  | 13,433 13,498 | 48.7% 49.0% | 0.580 | 0.005 |
|  |  | 1 2 | F91 | Conduct disorders |  | 8,999 9,101 | 32.6% 33.0% | 0.355 | 0.008 |
|  |  | 1 2 | F42 | Obsessive-compulsive disorder |  | 1,059 1,066 | 3.8% 3.9% | 0.877 | 0.001 |
|  |  | 1 2 | F84 | Pervasive developmental disorders |  | 2,999 3,021 | 10.9% 11.0% | 0.764 | 0.003 |
|  |  | 1 2 | F51 | Sleep disorders not due to a substance or known physiological condition |  | 1,626 1,601 | 5.9% 5.8% | 0.650 | 0.004 |
|  |  | 1 2 | F50 | Eating disorders |  | 1,076 1,015 | 3.9% 3.7% | 0.174 | 0.012 |
|  |  | 1 2 | F10-F19 | Mental and behavioral disorders due to psychoactive substance use |  | 1,746 1,648 | 6.3% 6.0% | 0.082 | 0.015 |
|  |  | 1 2 | F20-F29 | Schizophrenia, schizotypal, delusional, and other non-mood psychotic disorders |  | 719 674 | 2.6% 2.4% | 0.222 | 0.010 |
|  |  | 1 2 | F70-F79 | Intellectual Disabilities |  | 768 685 | 2.8% 2.5% | 0.027 | 0.019 |
|  |  | 1 2 | F41.0 | Panic disorder [episodic paroxysmal anxiety] |  | 1,024 964 | 3.7% 3.5% | 0.170 | 0.012 |
|  |  | 1 2 | F32 | Depressive episode |  | 8,492 8,238 | 30.8% 29.9% | 0.019 | 0.020 |
|  |  | 1 2 | F33 | Major depressive disorder, recurrent |  | 3,651 3,698 | 13.2% 13.4% | 0.556 | 0.005 |
|  |  | 1 2 | F34.81 | Disruptive mood dysregulation disorder |  | 2,733 2,706 | 9.9% 9.8% | 0.700 | 0.003 |
|  |  | 1 2 | F31 | Bipolar disorder |  | 1,255 1,108 | 4.6% 4.0% | 0.002 | 0.026 |
|  |  | 1 2 | F60.3 | Borderline personality disorder |  | 602 547 | 2.2% 2.0% | 0.101 | 0.014 |
|  |  | 1 2 | F95 | Tic disorder |  | 674 585 | 2.4% 2.1% | 0.011 | 0.022 |

**Supplement Analysis 5. Treatment Trends by Age and Gender**

**SA5.1. Cohorts: 6-11 ADHD Cohort with PTSD (vs 6-11 ADHD Cohort without PTSD)**

### Query Criteria for Cohort 1 (query name: ADHD+PTSD_6-11)

This query was run on the network Research with 100 HCO(s) queried and 100 HCO(s) responded. A total of 67 provider(s) responded with patients. The final cohort included 5,106 patients who matched the query criteria listed in the table below.

| Cohort 1 | | | | | |
| --- | --- | --- | --- | --- | --- |
|  | must have |  | demographics | Age | Age (between 6 and 11 years (most recent occurrence)) |
|  |  | and | diagnosis | UMLS:ICD10CM:F90 | Attention-deficit hyperactivity disorders |
|  |  | and | diagnosis | UMLS:ICD10CM:F43.1 | Post-traumatic stress disorder (PTSD) |

### Query Criteria for Cohort 2 (query name: ADHD_6-11)

This query was run on the network Research with 100 HCO(s) queried and 100 HCO(s) responded. A total of 87 provider(s) responded with patients. The final cohort included 218,984 patients who matched the query criteria listed in the table below.

| Cohort 2 | | | | | |
| --- | --- | --- | --- | --- | --- |
|  | must have |  | demographics | Age | Age (between 6 and 11 years (most recent occurrence)) |
|  |  | and | diagnosis | UMLS:ICD10CM:F90 | Attention-deficit hyperactivity disorders |
|  | cannot have |  | diagnosis | UMLS:ICD10CM:F43.1 | Post-traumatic stress disorder (PTSD) |

Propensity Score Matching

| **Cohort 1 (N = 4,845) and cohort 2 (N = 4,845) characteristics after propensity score matching** | | | | | | | | | |
| --- | --- | --- | --- | --- | --- | --- | --- | --- | --- |
|  | **Demographics** | | | | | | | | |
|  |  | Cohort | |  | Mean ± SD | Patients | % of Cohort | P-Value | Std diff. |
|  |  | 1 2 | AI | Age at Index | 7.2 +/- 1.8 7.2 +/- 1.8 | 4,845 4,845 | 100% 100% | 0.627 | 0.010 |
|  |  | 1 2 | 2186-5 | Not Hispanic or Latino |  | 3,384 3,391 | 69.8% 70.0% | 0.877 | 0.003 |
|  |  | 1 2 | 2054-5 | Black or African American |  | 1,034 1,036 | 21.3% 21.4% | 0.960 | 0.001 |
|  |  | 1 2 | M | Male |  | 2,990 2,988 | 61.7% 61.7% | 0.967 | 0.001 |
|  | **Diagnosis** | | | | | | | | |
|  |  | Cohort | |  | Mean ± SD | Patients | % of Cohort | P-Value | Std diff. |
|  |  | 1 2 | F41.9 | Anxiety disorder, unspecified |  | 1,204 1,262 | 24.9% 26.0% | 0.176 | 0.027 |
|  |  | 1 2 | F41.1 | Generalized anxiety disorder |  | 440 462 | 9.1% 9.5% | 0.442 | 0.016 |
|  |  | 1 2 | F30-F39 | Mood [affective] disorders |  | 1,007 993 | 20.8% 20.5% | 0.725 | 0.007 |
|  |  | 1 2 | F91 | Conduct disorders |  | 1,591 1,599 | 32.8% 33.0% | 0.863 | 0.004 |
|  |  | 1 2 | F42 | Obsessive-compulsive disorder |  | 90 89 | 1.9% 1.8% | 0.940 | 0.002 |
|  |  | 1 2 | F84 | Pervasive developmental disorders |  | 586 561 | 12.1% 11.6% | 0.432 | 0.016 |
|  |  | 1 2 | F51 | Sleep disorders not due to a substance or known physiological condition |  | 373 372 | 7.7% 7.7% | 0.970 | 0.001 |
|  |  | 1 2 | F50 | Eating disorders |  | 116 105 | 2.4% 2.2% | 0.454 | 0.015 |
|  |  | 1 2 | F10-F19 | Mental and behavioral disorders due to psychoactive substance use |  | 56 61 | 1.2% 1.3% | 0.642 | 0.009 |
|  |  | 1 2 | F20-F29 | Schizophrenia, schizotypal, delusional, and other non-mood psychotic disorders |  | 42 31 | 0.9% 0.6% | 0.196 | 0.026 |
|  |  | 1 2 | F70-F79 | Intellectual Disabilities |  | 107 95 | 2.2% 2.0% | 0.394 | 0.017 |
|  |  | 1 2 | F41.0 | Panic disorder [episodic paroxysmal anxiety] |  | 41 36 | 0.8% 0.7% | 0.567 | 0.012 |
|  |  | 1 2 | F32 | Depressive episode |  | 453 444 | 9.3% 9.2% | 0.752 | 0.006 |
|  |  | 1 2 | F33 | Major depressive disorder, recurrent |  | 88 66 | 1.8% 1.4% | 0.074 | 0.036 |
|  |  | 1 2 | F34.81 | Disruptive mood dysregulation disorder |  | 398 386 | 8.2% 8.0% | 0.655 | 0.009 |
|  |  | 1 2 | F31 | Bipolar disorder |  | 39 29 | 0.8% 0.6% | 0.224 | 0.025 |
|  |  | 1 2 | F60.3 | Borderline personality disorder |  | 48 49 | 1.0% 1.0% | 0.919 | 0.002 |
|  |  | 1 2 | F95 | Tic disorder |  | 92 81 | 1.9% 1.7% | 0.399 | 0.017 |

**SA5.2. Cohorts: 6-11 Male ADHD Cohort with PTSD (vs 6-11ADHD Male Cohort without PTSD)**

### Query Criteria for Cohort 1 (query name: ADHD+PTSD_6-11M)

This query was run on the network Research with 100 HCO(s) queried and 100 HCO(s) responded. A total of 66 provider(s) responded with patients. The final cohort included 3,167 patients who matched the query criteria listed in the table below.

| Cohort 1 | | | | | |
| --- | --- | --- | --- | --- | --- |
|  | must have |  | demographics | Age | Age (between 6 and 11 years (most recent occurrence)) |
|  |  | and | demographics | UMLS:HL7V3.0:Gender:M | Male |
|  |  | and | diagnosis | UMLS:ICD10CM:F90 | Attention-deficit hyperactivity disorders |
|  |  | and | diagnosis | UMLS:ICD10CM:F43.1 | Post-traumatic stress disorder (PTSD) |

### Query Criteria for Cohort 2 (query name: ADHD_6-11M)

This query was run on the network Research with 100 HCO(s) queried and 100 HCO(s) responded. A total of 87 provider(s) responded with patients. The final cohort included 153,508 patients who matched the query criteria listed in the table below.

| Cohort 2 | | | | | |
| --- | --- | --- | --- | --- | --- |
|  | must have |  | demographics | Age | Age (between 6 and 11 years (most recent occurrence)) |
|  |  | and | demographics | UMLS:HL7V3.0:Gender:M | Male |
|  |  | and | diagnosis | UMLS:ICD10CM:F90 | Attention-deficit hyperactivity disorders |
|  | cannot have |  | diagnosis | UMLS:ICD10CM:F43.1 | Post-traumatic stress disorder (PTSD) |

Propensity Score Matching

| **Cohort 1 (N = 2,998) and cohort 2 (N = 2,998) characteristics after propensity score matching** | | | | | | | | | |
| --- | --- | --- | --- | --- | --- | --- | --- | --- | --- |
|  | **Demographics** | | | | | | | | |
|  |  | Cohort | |  | Mean ± SD | Patients | % of Cohort | P-Value | Std diff. |
|  |  | 1 2 | AI | Age at Index | 7.1 +/- 1.8 7.2 +/- 1.8 | 2,998 2,998 | 100% 100% | 0.637 | 0.012 |
|  |  | 1 2 | 2186-5 | Not Hispanic or Latino |  | 2,099 2,107 | 70.0% 70.3% | 0.821 | 0.006 |
|  |  | 1 2 | 2054-5 | Black or African American |  | 624 604 | 20.8% 20.1% | 0.522 | 0.017 |
|  |  | 1 2 | M | Male |  | 2,998 2,998 | 100% 100% | -- | -- |
|  | **Diagnosis** | | | | | | | | |
|  |  | Cohort | |  | Mean ± SD | Patients | % of Cohort | P-Value | Std diff. |
|  |  | 1 2 | F41.9 | Anxiety disorder, unspecified |  | 687 709 | 22.9% 23.6% | 0.501 | 0.017 |
|  |  | 1 2 | F41.1 | Generalized anxiety disorder |  | 244 261 | 8.1% 8.7% | 0.429 | 0.020 |
|  |  | 1 2 | F30-F39 | Mood [affective] disorders |  | 628 615 | 20.9% 20.5% | 0.679 | 0.011 |
|  |  | 1 2 | F91 | Conduct disorders |  | 1,027 1,035 | 34.3% 34.5% | 0.828 | 0.006 |
|  |  | 1 2 | F42 | Obsessive-compulsive disorder |  | 55 58 | 1.8% 1.9% | 0.776 | 0.007 |
|  |  | 1 2 | F84 | Pervasive developmental disorders |  | 429 407 | 14.3% 13.6% | 0.412 | 0.021 |
|  |  | 1 2 | F51 | Sleep disorders not due to a substance or known physiological condition |  | 216 214 | 7.2% 7.1% | 0.920 | 0.003 |
|  |  | 1 2 | F50 | Eating disorders |  | 60 55 | 2.0% 1.8% | 0.638 | 0.012 |
|  |  | 1 2 | F10-F19 | Mental and behavioral disorders due to psychoactive substance use |  | 36 39 | 1.2% 1.3% | 0.727 | 0.009 |
|  |  | 1 2 | F20-F29 | Schizophrenia, schizotypal, delusional, and other non-mood psychotic disorders |  | 23 18 | 0.8% 0.6% | 0.433 | 0.020 |
|  |  | 1 2 | F70-F79 | Intellectual Disabilities |  | 71 69 | 2.4% 2.3% | 0.864 | 0.004 |
|  |  | 1 2 | F41.0 | Panic disorder [episodic paroxysmal anxiety] |  | 19 15 | 0.6% 0.5% | 0.491 | 0.018 |
|  |  | 1 2 | F32 | Depressive episode |  | 265 249 | 8.8% 8.3% | 0.460 | 0.019 |
|  |  | 1 2 | F33 | Major depressive disorder, recurrent |  | 49 42 | 1.6% 1.4% | 0.460 | 0.019 |
|  |  | 1 2 | F34.81 | Disruptive mood dysregulation disorder |  | 268 239 | 8.9% 8.0% | 0.178 | 0.035 |
|  |  | 1 2 | F31 | Bipolar disorder |  | 27 24 | 0.9% 0.8% | 0.673 | 0.011 |
|  |  | 1 2 | F60.3 | Borderline personality disorder |  | 32 29 | 1.1% 1.0% | 0.699 | 0.010 |
|  |  | 1 2 | F95 | Tic disorder |  | 66 62 | 2.2% 2.1% | 0.721 | 0.009 |

**SA5.3. Cohorts: 6-11 Female ADHD Cohort with PTSD (vs 6-11ADHD Female Cohort without PTSD)**

### Query Criteria for Cohort 1 (query name: ADHD+PTSD_6-11F)

This query was run on the network Research with 100 HCO(s) queried and 100 HCO(s) responded. A total of 58 provider(s) responded with patients. The final cohort included 1,832 patients who matched the query criteria listed in the table below.

| Cohort 1 | | | | | |
| --- | --- | --- | --- | --- | --- |
|  | must have |  | demographics | Age | Age (between 6 and 11 years (most recent occurrence)) |
|  |  | and | demographics | UMLS:HL7V3.0:Gender:F | Female |
|  |  | and | diagnosis | UMLS:ICD10CM:F90 | Attention-deficit hyperactivity disorders |
|  |  | and | diagnosis | UMLS:ICD10CM:F43.1 | Post-traumatic stress disorder (PTSD) |

### Query Criteria for Cohort 2 (query name: ADHD_6-11F)

This query was run on the network Research with 100 HCO(s) queried and 100 HCO(s) responded. A total of 87 provider(s) responded with patients. The final cohort included 62,920 patients who matched the query criteria listed in the table below.

| Cohort 2 | | | | | |
| --- | --- | --- | --- | --- | --- |
|  | must have |  | demographics | Age | Age (between 6 and 11 years (most recent occurrence)) |
|  |  | and | demographics | UMLS:HL7V3.0:Gender:F | Female |
|  |  | and | diagnosis | UMLS:ICD10CM:F90 | Attention-deficit hyperactivity disorders |
|  | cannot have |  | diagnosis | UMLS:ICD10CM:F43.1 | Post-traumatic stress disorder (PTSD) |

Propensity Score Matching

| **Cohort 1 (N = 1,147) and cohort 2 (N = 1,147) characteristics after propensity score matching** | | | | | | | | | |
| --- | --- | --- | --- | --- | --- | --- | --- | --- | --- |
|  | **Demographics** | | | | | | | | |
|  |  | Cohort | |  | Mean ± SD | Patients | % of Cohort | P-Value | Std diff. |
|  |  | 1 2 | AI | Age at Index | 7.3 +/- 1.8 7.2 +/- 1.9 | 1,147 1,147 | 100% 100% | 0.222 | 0.051 |
|  |  | 1 2 | 2186-5 | Not Hispanic or Latino |  | 807 817 | 70.4% 71.2% | 0.646 | 0.019 |
|  |  | 1 2 | 2054-5 | Black or African American |  | 333 342 | 29.0% 29.8% | 0.680 | 0.017 |
|  |  | 1 2 | M | Male |  | 0 0 | 0% 0% | -- | -- |
|  | **Diagnosis** | | | | | | | | |
|  |  | Cohort | |  | Mean ± SD | Patients | % of Cohort | P-Value | Std diff. |
|  |  | 1 2 | F41.9 | Anxiety disorder, unspecified |  | 283 310 | 24.7% 27.0% | 0.198 | 0.054 |
|  |  | 1 2 | F41.1 | Generalized anxiety disorder |  | 120 122 | 10.5% 10.6% | 0.892 | 0.006 |
|  |  | 1 2 | F30-F39 | Mood [affective] disorders |  | 219 205 | 19.1% 17.9% | 0.451 | 0.031 |
|  |  | 1 2 | F91 | Conduct disorders |  | 334 348 | 29.1% 30.3% | 0.522 | 0.027 |
|  |  | 1 2 | F42 | Obsessive-compulsive disorder |  | 20 17 | 1.7% 1.5% | 0.619 | 0.021 |
|  |  | 1 2 | F84 | Pervasive developmental disorders |  | 93 94 | 8.1% 8.2% | 0.939 | 0.003 |
|  |  | 1 2 | F51 | Sleep disorders not due to a substance or known physiological condition |  | 96 105 | 8.4% 9.2% | 0.506 | 0.028 |
|  |  | 1 2 | F50 | Eating disorders |  | 32 33 | 2.8% 2.9% | 0.900 | 0.005 |
|  |  | 1 2 | F10-F19 | Mental and behavioral disorders due to psychoactive substance use |  | 10 10 | 0.9% 0.9% | 1 | <0.001 |
|  |  | 1 2 | F20-F29 | Schizophrenia, schizotypal, delusional, and other non-mood psychotic disorders |  | 10 10 | 0.9% 0.9% | 1 | <0.001 |
|  |  | 1 2 | F70-F79 | Intellectual Disabilities |  | 23 16 | 2.0% 1.4% | 0.258 | 0.047 |
|  |  | 1 2 | F41.0 | Panic disorder [episodic paroxysmal anxiety] |  | 13 10 | 1.1% 0.9% | 0.530 | 0.026 |
|  |  | 1 2 | F32 | Depressive episode |  | 114 104 | 9.9% 9.1% | 0.476 | 0.030 |
|  |  | 1 2 | F33 | Major depressive disorder, recurrent |  | 23 16 | 2.0% 1.4% | 0.258 | 0.047 |
|  |  | 1 2 | F34.81 | Disruptive mood dysregulation disorder |  | 76 78 | 6.6% 6.8% | 0.867 | 0.007 |
|  |  | 1 2 | F31 | Bipolar disorder |  | 10 10 | 0.9% 0.9% | 1 | <0.001 |
|  |  | 1 2 | F60.3 | Borderline personality disorder |  | 12 12 | 1.0% 1.0% | 1 | <0.001 |
|  |  | 1 2 | F95 | Tic disorder |  | 13 14 | 1.1% 1.2% | 0.846 | 0.008 |

**SA5.4. Cohorts: 12-18 ADHD Cohort with PTSD (vs 12-18 ADHD Female Cohort without PTSD)**

### Query Criteria for Cohort 1 (query name: ADHD+PTSD_12-18)

This query was run on the network Research with 100 HCO(s) queried and 100 HCO(s) responded. A total of 67 provider(s) responded with patients. The final cohort included 25,235 patients who matched the query criteria listed in the table below.

| Cohort 1 | | | | | |
| --- | --- | --- | --- | --- | --- |
|  | must have |  | demographics | Age | Age (between 12 and 18 years (most recent occurrence)) |
|  |  | and | diagnosis | UMLS:ICD10CM:F90 | Attention-deficit hyperactivity disorders |
|  |  | and | diagnosis | UMLS:ICD10CM:F43.1 | Post-traumatic stress disorder (PTSD) |

### Query Criteria for Cohort 2 (query name: ADHD_12-18)

This query was run on the network Research with 100 HCO(s) queried and 100 HCO(s) responded. A total of 92 provider(s) responded with patients. The final cohort included 464,804 patients who matched the query criteria listed in the table below.

| Cohort 2 | | | | | |
| --- | --- | --- | --- | --- | --- |
|  | must have |  | demographics | Age | Age (between 12 and 18 years (most recent occurrence)) |
|  |  | and | diagnosis | UMLS:ICD10CM:F90 | Attention-deficit hyperactivity disorders |
|  | cannot have |  | diagnosis | UMLS:ICD10CM:F43.1 | Post-traumatic stress disorder (PTSD) |

Propensity Score Matching

| **Cohort 1 (N = 23,321) and cohort 2 (N = 23,321) characteristics after propensity score matching** | | | | | | | | | |
| --- | --- | --- | --- | --- | --- | --- | --- | --- | --- |
|  | **Demographics** | | | | | | | | |
|  |  | Cohort | |  | Mean ± SD | Patients | % of Cohort | P-Value | Std diff. |
|  |  | 1 2 | AI | Age at Index | 11.5 +/- 3.2 11.5 +/- 3.3 | 23,321 23,321 | 100% 100% | 0.658 | 0.004 |
|  |  | 1 2 | 2186-5 | Not Hispanic or Latino |  | 16,664 16,689 | 71.5% 71.6% | 0.798 | 0.002 |
|  |  | 1 2 | 2054-5 | Black or African American |  | 4,680 4,597 | 20.1% 19.7% | 0.336 | 0.009 |
|  |  | 1 2 | M | Male |  | 11,732 11,691 | 50.3% 50.1% | 0.704 | 0.004 |
|  | **Diagnosis** | | | | | | | | |
|  |  | Cohort | |  | Mean ± SD | Patients | % of Cohort | P-Value | Std diff. |
|  |  | 1 2 | F41.9 | Anxiety disorder, unspecified |  | 8,534 8,648 | 36.6% 37.1% | 0.274 | 0.010 |
|  |  | 1 2 | F41.1 | Generalized anxiety disorder |  | 4,667 4,767 | 20.0% 20.4% | 0.249 | 0.011 |
|  |  | 1 2 | F30-F39 | Mood [affective] disorders |  | 12,653 12,734 | 54.3% 54.6% | 0.451 | 0.007 |
|  |  | 1 2 | F91 | Conduct disorders |  | 7,557 7,654 | 32.4% 32.8% | 0.338 | 0.009 |
|  |  | 1 2 | F42 | Obsessive-compulsive disorder |  | 942 961 | 4.0% 4.1% | 0.657 | 0.004 |
|  |  | 1 2 | F84 | Pervasive developmental disorders |  | 2,385 2,378 | 10.2% 10.2% | 0.915 | 0.001 |
|  |  | 1 2 | F51 | Sleep disorders not due to a substance or known physiological condition |  | 1,291 1,237 | 5.5% 5.3% | 0.269 | 0.010 |
|  |  | 1 2 | F50 | Eating disorders |  | 937 883 | 4.0% 3.8% | 0.197 | 0.012 |
|  |  | 1 2 | F10-F19 | Mental and behavioral disorders due to psychoactive substance use |  | 1,670 1,611 | 7.2% 6.9% | 0.285 | 0.010 |
|  |  | 1 2 | F20-F29 | Schizophrenia, schizotypal, delusional, and other non-mood psychotic disorders |  | 686 657 | 2.9% 2.8% | 0.422 | 0.007 |
|  |  | 1 2 | F70-F79 | Intellectual Disabilities |  | 690 611 | 3.0% 2.6% | 0.026 | 0.021 |
|  |  | 1 2 | F41.0 | Panic disorder [episodic paroxysmal anxiety] |  | 1,002 926 | 4.3% 4.0% | 0.077 | 0.016 |
|  |  | 1 2 | F32 | Depressive episode |  | 8,127 7,900 | 34.8% 33.9% | 0.027 | 0.020 |
|  |  | 1 2 | F33 | Major depressive disorder, recurrent |  | 3,606 3,646 | 15.5% 15.6% | 0.609 | 0.005 |
|  |  | 1 2 | F34.81 | Disruptive mood dysregulation disorder |  | 2,473 2,438 | 10.6% 10.5% | 0.597 | 0.005 |
|  |  | 1 2 | F31 | Bipolar disorder |  | 1,259 1,130 | 5.4% 4.8% | 0.007 | 0.025 |
|  |  | 1 2 | F60.3 | Borderline personality disorder |  | 561 505 | 2.4% 2.2% | 0.083 | 0.016 |
|  |  | 1 2 | F95 | Tic disorder |  | 601 523 | 2.6% 2.2% | 0.019 | 0.022 |

**SA5.5. Cohorts: 12-18 Male ADHD Cohort with PTSD (vs 12-18 Male ADHD Cohort without PTSD)**

### Query Criteria for Cohort 1 (query name: ADHD+PTSD_12-18M)

This query was run on the network Research with 100 HCO(s) queried and 100 HCO(s) responded. A total of 67 provider(s) responded with patients. The final cohort included 12,503 patients who matched the query criteria listed in the table below.

| Cohort 1 | | | | | |
| --- | --- | --- | --- | --- | --- |
|  | must have |  | demographics | Age | Age (between 12 and 18 years (most recent occurrence)) |
|  |  | and | demographics | UMLS:HL7V3.0:Gender:M | Male |
|  |  | and | diagnosis | UMLS:ICD10CM:F90 | Attention-deficit hyperactivity disorders |
|  |  | and | diagnosis | UMLS:ICD10CM:F43.1 | Post-traumatic stress disorder (PTSD) |

### Query Criteria for Cohort 2 (query name: ADHD_12-18M)

This query was run on the network Research with 100 HCO(s) queried and 100 HCO(s) responded. A total of 91 provider(s) responded with patients. The final cohort included 311,540 patients who matched the query criteria listed in the table below.

| Cohort 2 | | | | | |
| --- | --- | --- | --- | --- | --- |
|  | must have |  | demographics | Age | Age (between 12 and 18 years (most recent occurrence)) |
|  |  | and | demographics | UMLS:HL7V3.0:Gender:M | Male |
|  |  | and | diagnosis | UMLS:ICD10CM:F90 | Attention-deficit hyperactivity disorders |
|  | cannot have |  | diagnosis | UMLS:ICD10CM:F43.1 | Post-traumatic stress disorder (PTSD) |

Propensity Score Matching

| **Cohort 1 (N = 11,864) and cohort 2 (N = 11,864) characteristics after propensity score matching** | | | | | | | | | |
| --- | --- | --- | --- | --- | --- | --- | --- | --- | --- |
|  | **Demographics** | | | | | | | | |
|  |  | Cohort | |  | Mean ± SD | Patients | % of Cohort | P-Value | Std diff. |
|  |  | 1 2 | AI | Age at Index | 10.8 +/- 3.2 10.8 +/- 3.3 | 11,864 11,864 | 100% 100% | 0.305 | 0.013 |
|  |  | 1 2 | 2186-5 | Not Hispanic or Latino |  | 8,759 8,737 | 73.8% 73.6% | 0.746 | 0.004 |
|  |  | 1 2 | 2054-5 | Black or African American |  | 2,586 2,511 | 21.8% 21.2% | 0.236 | 0.015 |
|  |  | 1 2 | M | Male |  | 11,864 11,864 | 100% 100% | -- | -- |
|  | **Diagnosis** | | | | | | | | |
|  |  | Cohort | |  | Mean ± SD | Patients | % of Cohort | P-Value | Std diff. |
|  |  | 1 2 | F41.9 | Anxiety disorder, unspecified |  | 3,743 3,852 | 31.5% 32.5% | 0.129 | 0.020 |
|  |  | 1 2 | F41.1 | Generalized anxiety disorder |  | 1,607 1,658 | 13.5% 14.0% | 0.336 | 0.012 |
|  |  | 1 2 | F30-F39 | Mood [affective] disorders |  | 5,474 5,489 | 46.1% 46.3% | 0.845 | 0.003 |
|  |  | 1 2 | F91 | Conduct disorders |  | 4,830 4,850 | 40.7% 40.9% | 0.792 | 0.003 |
|  |  | 1 2 | F42 | Obsessive-compulsive disorder |  | 404 368 | 3.4% 3.1% | 0.188 | 0.017 |
|  |  | 1 2 | F84 | Pervasive developmental disorders |  | 1,593 1,605 | 13.4% 13.5% | 0.820 | 0.003 |
|  |  | 1 2 | F51 | Sleep disorders not due to a substance or known physiological condition |  | 608 563 | 5.1% 4.7% | 0.177 | 0.018 |
|  |  | 1 2 | F50 | Eating disorders |  | 200 188 | 1.7% 1.6% | 0.539 | 0.008 |
|  |  | 1 2 | F10-F19 | Mental and behavioral disorders due to psychoactive substance use |  | 755 733 | 6.4% 6.2% | 0.556 | 0.008 |
|  |  | 1 2 | F20-F29 | Schizophrenia, schizotypal, delusional, and other non-mood psychotic disorders |  | 363 332 | 3.1% 2.8% | 0.233 | 0.015 |
|  |  | 1 2 | F70-F79 | Intellectual Disabilities |  | 410 370 | 3.5% 3.1% | 0.145 | 0.019 |
|  |  | 1 2 | F41.0 | Panic disorder [episodic paroxysmal anxiety] |  | 231 243 | 1.9% 2.0% | 0.578 | 0.007 |
|  |  | 1 2 | F32 | Depressive episode |  | 3,102 3,046 | 26.1% 25.7% | 0.407 | 0.011 |
|  |  | 1 2 | F33 | Major depressive disorder, recurrent |  | 992 1,027 | 8.4% 8.7% | 0.415 | 0.011 |
|  |  | 1 2 | F34.81 | Disruptive mood dysregulation disorder |  | 1,490 1,463 | 12.6% 12.3% | 0.595 | 0.007 |
|  |  | 1 2 | F31 | Bipolar disorder |  | 512 476 | 4.3% 4.0% | 0.242 | 0.015 |
|  |  | 1 2 | F60.3 | Borderline personality disorder |  | 274 260 | 2.3% 2.2% | 0.540 | 0.008 |
|  |  | 1 2 | F95 | Tic disorder |  | 345 319 | 2.9% 2.7% | 0.306 | 0.013 |

**SA5.6. Cohorts: 12-18 Female ADHD Cohort with PTSD (vs 12-18 Female ADHD Cohort without PTSD)**

### Query Criteria for Cohort 1 (query name: ADHD+PTSD_12-18F)

This query was run on the network Research with 100 HCO(s) queried and 100 HCO(s) responded. A total of 66 provider(s) responded with patients. The final cohort included 12,165 patients who matched the query criteria listed in the table below.

| Cohort 1 | | | | | |
| --- | --- | --- | --- | --- | --- |
|  | must have |  | demographics | Age | Age (between 12 and 18 years (most recent occurrence)) |
|  |  | and | demographics | UMLS:HL7V3.0:Gender:F | Female |
|  |  | and | diagnosis | UMLS:ICD10CM:F90 | Attention-deficit hyperactivity disorders |
|  |  | and | diagnosis | UMLS:ICD10CM:F43.1 | Post-traumatic stress disorder (PTSD) |

### Query Criteria for Cohort 2 (query name: ADHD_12-18F)

This query was run on the network Research with 100 HCO(s) queried and 100 HCO(s) responded. A total of 89 provider(s) responded with patients. The final cohort included 146,688 patients who matched the query criteria listed in the table below.

| Cohort | | | | | |
| --- | --- | --- | --- | --- | --- |
|  | must have |  | demographics | Age | Age (between 12 and 18 years (most recent occurrence)) |
|  |  | and | demographics | UMLS:HL7V3.0:Gender:F | Female |
|  |  | and | diagnosis | UMLS:ICD10CM:F90 | Attention-deficit hyperactivity disorders |
|  | cannot have |  | diagnosis | UMLS:ICD10CM:F43.1 | Post-traumatic stress disorder (PTSD) |

Propensity Score Matching

| **Cohort 1 (N = 10,611) and cohort 2 (N = 10,611) characteristics after propensity score matching** | | | | | | | | | |
| --- | --- | --- | --- | --- | --- | --- | --- | --- | --- |
|  | **Demographics** | | | | | | | | |
|  |  | Cohort | |  | Mean ± SD | Patients | % of Cohort | P-Value | Std diff. |
|  |  | 1 2 | AI | Age at Index | 12.3 +/- 3.0 12.3 +/- 3.1 | 10,611 10,611 | 100% 100% | 0.505 | 0.009 |
|  |  | 1 2 | 2186-5 | Not Hispanic or Latino |  | 7,666 7,708 | 72.2% 72.6% | 0.519 | 0.009 |
|  |  | 1 2 | 2054-5 | Black or African American |  | 1,988 1,983 | 18.7% 18.7% | 0.930 | 0.001 |
|  |  | 1 2 | M | Male |  | 0 0 | 0% 0% | -- | -- |
|  | **Diagnosis** | | | | | | | | |
|  |  | Cohort | |  | Mean ± SD | Patients | % of Cohort | P-Value | Std diff. |
|  |  | 1 2 | F41.9 | Anxiety disorder, unspecified |  | 4,615 4,567 | 43.5% 43.0% | 0.506 | 0.009 |
|  |  | 1 2 | F41.1 | Generalized anxiety disorder |  | 2,857 2,914 | 26.9% 27.5% | 0.379 | 0.012 |
|  |  | 1 2 | F30-F39 | Mood [affective] disorders |  | 6,835 6,890 | 64.4% 64.9% | 0.430 | 0.011 |
|  |  | 1 2 | F91 | Conduct disorders |  | 2,663 2,741 | 25.1% 25.8% | 0.219 | 0.017 |
|  |  | 1 2 | F42 | Obsessive-compulsive disorder |  | 541 607 | 5.1% 5.7% | 0.045 | 0.027 |
|  |  | 1 2 | F84 | Pervasive developmental disorders |  | 759 722 | 7.2% 6.8% | 0.319 | 0.014 |
|  |  | 1 2 | F51 | Sleep disorders not due to a substance or known physiological condition |  | 636 644 | 6.0% 6.1% | 0.818 | 0.003 |
|  |  | 1 2 | F50 | Eating disorders |  | 720 706 | 6.8% 6.7% | 0.701 | 0.005 |
|  |  | 1 2 | F10-F19 | Mental and behavioral disorders due to psychoactive substance use |  | 917 892 | 8.6% 8.4% | 0.539 | 0.008 |
|  |  | 1 2 | F20-F29 | Schizophrenia, schizotypal, delusional, and other non-mood psychotic disorders |  | 332 303 | 3.1% 2.9% | 0.243 | 0.016 |
|  |  | 1 2 | F70-F79 | Intellectual Disabilities |  | 263 207 | 2.5% 2.0% | 0.009 | 0.036 |
|  |  | 1 2 | F41.0 | Panic disorder [episodic paroxysmal anxiety] |  | 684 632 | 6.4% 6.0% | 0.139 | 0.020 |
|  |  | 1 2 | F32 | Depressive episode |  | 4,947 4,811 | 46.6% 45.3% | 0.061 | 0.026 |
|  |  | 1 2 | F33 | Major depressive disorder, recurrent |  | 2,494 2,541 | 23.5% 23.9% | 0.448 | 0.010 |
|  |  | 1 2 | F34.81 | Disruptive mood dysregulation disorder |  | 913 920 | 8.6% 8.7% | 0.864 | 0.002 |
|  |  | 1 2 | F31 | Bipolar disorder |  | 663 621 | 6.2% 5.9% | 0.227 | 0.017 |
|  |  | 1 2 | F60.3 | Borderline personality disorder |  | 285 247 | 2.7% 2.3% | 0.095 | 0.023 |
|  |  | 1 2 | F95 | Tic disorder |  | 247 192 | 2.3% 1.8% | 0.008 | 0.036 |

**Supplement Analysis 6. Treatment Trends by Race and Ethnicity**

**SA6.1. Cohorts: White ADHD Cohort with PTSD (vs White ADHD Cohort without PTSD)**

### Query Criteria for Cohort 1 (query name: ADHD+PTSD_W)

This query was run on the network Research with 100 HCO(s) queried and 100 HCO(s) responded. A total of 65 provider(s) responded with patients. The final cohort included 18,116 patients who matched the query criteria listed in the table below.

| Cohort 1 | | | | | |
| --- | --- | --- | --- | --- | --- |
|  | must have |  | demographics | Age | Age (between 6 and 18 years (most recent occurrence)) |
|  |  | and | diagnosis | UMLS:ICD10CM:F90 | Attention-deficit hyperactivity disorders |
|  |  | and | diagnosis | UMLS:ICD10CM:F43.1 | Post-traumatic stress disorder (PTSD) |
|  |  | and | demographics | UMLS:HL7V3.0:Race:2106-3 | White |

### Query Criteria for Cohort 2 (query name: ADHD_W)

This query was run on the network Research with 100 HCO(s) queried and 100 HCO(s) responded. A total of 72 provider(s) responded with patients. The final cohort included 405,912 patients who matched the query criteria listed in the table below.

| Cohort 2 | | | | | |
| --- | --- | --- | --- | --- | --- |
|  | must have |  | demographics | Age | Age (between 6 and 18 years (most recent occurrence)) |
|  |  | and | diagnosis | UMLS:ICD10CM:F90 | Attention-deficit hyperactivity disorders |
|  |  | and | demographics | UMLS:HL7V3.0:Race:2106-3 | White |
|  | cannot have |  | diagnosis | UMLS:ICD10CM:F43.1 | Post-traumatic stress disorder (PTSD) |

Propensity Score Matching

| **Cohort 1 (N = 17,668) and cohort 2 (N = 17,668) characteristics after propensity score matching** | | | | | | | | | |
| --- | --- | --- | --- | --- | --- | --- | --- | --- | --- |
|  | **Demographics** | | | | | | | | |
|  |  | Cohort | |  | Mean ± SD | Patients | % of Cohort | P-Value | Std diff. |
|  |  | 1 2 | AI | Age at Index | 11.0 +/- 3.4 11.0 +/- 3.5 | 17,668 17,668 | 100% 100% | 0.948 | 0.001 |
|  |  | 1 2 | 2186-5 | Not Hispanic or Latino |  | 14,092 14,107 | 79.8% 79.8% | 0.842 | 0.002 |
|  |  | 1 2 | 2054-5 | Black or African American |  | 0 0 | 0% 0% | -- | -- |
|  |  | 1 2 | M | Male |  | 9,191 9,198 | 52.0% 52.1% | 0.941 | 0.001 |
|  | **Diagnosis** | | | | | | | | |
|  |  | Cohort | |  | Mean ± SD | Patients | % of Cohort | P-Value | Std diff. |
|  |  | 1 2 | F41.9 | Anxiety disorder, unspecified |  | 7,003 7,089 | 39.6% 40.1% | 0.350 | 0.010 |
|  |  | 1 2 | F41.1 | Generalized anxiety disorder |  | 3,642 3,714 | 20.6% 21.0% | 0.345 | 0.010 |
|  |  | 1 2 | F30-F39 | Mood [affective] disorders |  | 9,077 9,105 | 51.4% 51.5% | 0.766 | 0.003 |
|  |  | 1 2 | F91 | Conduct disorders |  | 5,639 5,744 | 31.9% 32.5% | 0.232 | 0.013 |
|  |  | 1 2 | F42 | Obsessive-compulsive disorder |  | 804 816 | 4.6% 4.6% | 0.760 | 0.003 |
|  |  | 1 2 | F84 | Pervasive developmental disorders |  | 2,084 2,057 | 11.8% 11.6% | 0.655 | 0.005 |
|  |  | 1 2 | F51 | Sleep disorders not due to a substance or known physiological condition |  | 1,114 1,137 | 6.3% 6.4% | 0.616 | 0.005 |
|  |  | 1 2 | F50 | Eating disorders |  | 760 695 | 4.3% 3.9% | 0.082 | 0.019 |
|  |  | 1 2 | F10-F19 | Mental and behavioral disorders due to psychoactive substance use |  | 1,179 1,157 | 6.7% 6.5% | 0.638 | 0.005 |
|  |  | 1 2 | F20-F29 | Schizophrenia, schizotypal, delusional, and other non-mood psychotic disorders |  | 443 420 | 2.5% 2.4% | 0.428 | 0.008 |
|  |  | 1 2 | F70-F79 | Intellectual Disabilities |  | 495 414 | 2.8% 2.3% | 0.006 | 0.029 |
|  |  | 1 2 | F41.0 | Panic disorder [episodic paroxysmal anxiety] |  | 750 687 | 4.2% 3.9% | 0.090 | 0.018 |
|  |  | 1 2 | F32 | Depressive episode |  | 5,943 5,764 | 33.6% 32.6% | 0.043 | 0.022 |
|  |  | 1 2 | F33 | Major depressive disorder, recurrent |  | 2,626 2,610 | 14.9% 14.8% | 0.811 | 0.003 |
|  |  | 1 2 | F34.81 | Disruptive mood dysregulation disorder |  | 1,775 1,764 | 10.0% 10.0% | 0.845 | 0.002 |
|  |  | 1 2 | F31 | Bipolar disorder |  | 883 800 | 5.0% 4.5% | 0.038 | 0.022 |
|  |  | 1 2 | F60.3 | Borderline personality disorder |  | 434 382 | 2.5% 2.2% | 0.066 | 0.020 |
|  |  | 1 2 | F95 | Tic disorder |  | 539 465 | 3.1% 2.6% | 0.018 | 0.025 |

**SA6.2. Cohorts: Black ADHD Cohort with PTSD (vs Black ADHD Cohort without PTSD)**

### Query Criteria for Cohort 1 (query name: ADHD+PTSD_B)

This query was run on the network Research with 100 HCO(s) queried and 100 HCO(s) responded. A total of 63 provider(s) responded with patients. The final cohort included 6,058 patients who matched the query criteria listed in the table below.

| Cohort 1 | | | | | |
| --- | --- | --- | --- | --- | --- |
|  | must have |  | demographics | Age | Age (between 6 and 18 years (most recent occurrence)) |
|  |  | and | diagnosis | UMLS:ICD10CM:F90 | Attention-deficit hyperactivity disorders |
|  |  | and | diagnosis | UMLS:ICD10CM:F43.1 | Post-traumatic stress disorder (PTSD) |
|  |  | and | demographics | UMLS:HL7V3.0:Race:2054-5 | Black or African American |

### Query Criteria for Cohort 2 (query name: ADHD_B)

This query was run on the network Research with 100 HCO(s) queried and 100 HCO(s) responded. A total of 68 provider(s) responded with patients. The final cohort included 111,485 patients who matched the query criteria listed in the table below.

| Cohort 2 | | | | | |
| --- | --- | --- | --- | --- | --- |
|  | must have |  | demographics | Age | Age (between 6 and 18 years (most recent occurrence)) |
|  |  | and | diagnosis | UMLS:ICD10CM:F90 | Attention-deficit hyperactivity disorders |
|  |  | and | demographics | UMLS:HL7V3.0:Race:2054-5 | Black or African American |
|  | cannot have |  | diagnosis | UMLS:ICD10CM:F43.1 | Post-traumatic stress disorder (PTSD) |

Propensity Score Matching

| **Cohort 1 (N = 5,720) and cohort 2 (N = 5,720) characteristics after propensity score matching** | | | | | | | | | |
| --- | --- | --- | --- | --- | --- | --- | --- | --- | --- |
|  | **Demographics** | | | | | | | | |
|  |  | Cohort | |  | Mean ± SD | Patients | % of Cohort | P-Value | Std diff. |
|  |  | 1 2 | AI | Age at Index | 10.2 +/- 3.3 10.2 +/- 3.4 | 5,720 5,720 | 100% 100% | 0.430 | 0.015 |
|  |  | 1 2 | 2186-5 | Not Hispanic or Latino |  | 4,860 4,841 | 85.0% 84.6% | 0.621 | 0.009 |
|  |  | 1 2 | 2054-5 | Black or African American |  | 5,720 5,720 | 100% 100% | -- | -- |
|  |  | 1 2 | M | Male |  | 3,217 3,123 | 56.2% 54.6% | 0.077 | 0.033 |
|  | **Diagnosis** | | | | | | | | |
|  |  | Cohort | |  | Mean ± SD | Patients | % of Cohort | P-Value | Std diff. |
|  |  | 1 2 | F41.9 | Anxiety disorder, unspecified |  | 1,498 1,557 | 26.2% 27.2% | 0.212 | 0.023 |
|  |  | 1 2 | F41.1 | Generalized anxiety disorder |  | 582 616 | 10.2% 10.8% | 0.299 | 0.019 |
|  |  | 1 2 | F30-F39 | Mood [affective] disorders |  | 2,438 2,439 | 42.6% 42.6% | 0.985 | <0.001 |
|  |  | 1 2 | F91 | Conduct disorders |  | 2,137 2,118 | 37.4% 37.0% | 0.713 | 0.007 |
|  |  | 1 2 | F42 | Obsessive-compulsive disorder |  | 110 107 | 1.9% 1.9% | 0.837 | 0.004 |
|  |  | 1 2 | F84 | Pervasive developmental disorders |  | 437 387 | 7.6% 6.8% | 0.071 | 0.034 |
|  |  | 1 2 | F51 | Sleep disorders not due to a substance or known physiological condition |  | 314 304 | 5.5% 5.3% | 0.679 | 0.008 |
|  |  | 1 2 | F50 | Eating disorders |  | 124 125 | 2.2% 2.2% | 0.949 | 0.001 |
|  |  | 1 2 | F10-F19 | Mental and behavioral disorders due to psychoactive substance use |  | 285 254 | 5.0% 4.4% | 0.171 | 0.026 |
|  |  | 1 2 | F20-F29 | Schizophrenia, schizotypal, delusional, and other non-mood psychotic disorders |  | 166 165 | 2.9% 2.9% | 0.956 | 0.001 |
|  |  | 1 2 | F70-F79 | Intellectual Disabilities |  | 176 149 | 3.1% 2.6% | 0.129 | 0.028 |
|  |  | 1 2 | F41.0 | Panic disorder [episodic paroxysmal anxiety] |  | 110 92 | 1.9% 1.6% | 0.201 | 0.024 |
|  |  | 1 2 | F32 | Depressive episode |  | 1,443 1,409 | 25.2% 24.6% | 0.462 | 0.014 |
|  |  | 1 2 | F33 | Major depressive disorder, recurrent |  | 477 470 | 8.3% 8.2% | 0.812 | 0.004 |
|  |  | 1 2 | F34.81 | Disruptive mood dysregulation disorder |  | 636 572 | 11.1% 10% | 0.052 | 0.036 |
|  |  | 1 2 | F31 | Bipolar disorder |  | 225 208 | 3.9% 3.6% | 0.405 | 0.016 |
|  |  | 1 2 | F60.3 | Borderline personality disorder |  | 101 91 | 1.8% 1.6% | 0.467 | 0.014 |
|  |  | 1 2 | F95 | Tic disorder |  | 70 67 | 1.2% 1.2% | 0.797 | 0.005 |

**SA6.3. Cohorts: Non-Hispanic ADHD Cohort with PTSD (vs Non-Hispanic ADHD Cohort without PTSD)**

### Query Criteria for Cohort 1 (query name: ADHD+PTSD_NH)

This query was run on the network Research with 100 HCO(s) queried and 100 HCO(s) responded. A total of 61 provider(s) responded with patients. The final cohort included 21,647 patients who matched the query criteria listed in the table below.

| Cohort 1 | | | | | |
| --- | --- | --- | --- | --- | --- |
|  | must have |  | demographics | Age | Age (between 6 and 18 years (most recent occurrence)) |
|  |  | and | diagnosis | UMLS:ICD10CM:F90 | Attention-deficit hyperactivity disorders |
|  |  | and | diagnosis | UMLS:ICD10CM:F43.1 | Post-traumatic stress disorder (PTSD) |
|  |  | and | demographics | UMLS:HL7V3.0:Ethnicity:2186-5 | Not Hispanic or Latino |

### Query Criteria for Cohort 2 (query name: ADHD_NH)

This query was run on the network Research with 100 HCO(s) queried and 100 HCO(s) responded. A total of 62 provider(s) responded with patients. The final cohort included 464,261 patients who matched the query criteria listed in the table below.

| Cohort 2 | | | | | |
| --- | --- | --- | --- | --- | --- |
|  | must have |  | demographics | Age | Age (between 6 and 18 years (most recent occurrence)) |
|  |  | and | diagnosis | UMLS:ICD10CM:F90 | Attention-deficit hyperactivity disorders |
|  |  | and | demographics | UMLS:HL7V3.0:Ethnicity:2186-5 | Not Hispanic or Latino |
|  | cannot have |  | diagnosis | UMLS:ICD10CM:F43.1 | Post-traumatic stress disorder (PTSD) |

Propensity Score Matching

| **Cohort 1 (N = 17,593) and cohort 2 (N = 17,593) characteristics after propensity score matching** | | | | | | | | | |
| --- | --- | --- | --- | --- | --- | --- | --- | --- | --- |
|  | **Demographics** | | | | | | | | |
|  |  | Cohort | |  | Mean ± SD | Patients | % of Cohort | P-Value | Std diff. |
|  |  | 1 2 | AI | Age at Index | 10.6 +/- 3.4 10.6 +/- 3.5 | 17,593 17,593 | 100% 100% | 0.840 | 0.002 |
|  |  | 1 2 | 2186-5 | Not Hispanic or Latino |  | 17,593 17,593 | 100% 100% | -- | -- |
|  |  | 1 2 | 2054-5 | Black or African American |  | 4,701 4,580 | 26.7% 26.0% | 0.143 | 0.016 |
|  |  | 1 2 | M | Male |  | 9,379 9,278 | 53.3% 52.7% | 0.281 | 0.012 |
|  | **Diagnosis** | | | | | | | | |
|  |  | Cohort | |  | Mean ± SD | Patients | % of Cohort | P-Value | Std diff. |
|  |  | 1 2 | F41.9 | Anxiety disorder, unspecified |  | 6,373 6,438 | 36.2% 36.6% | 0.471 | 0.008 |
|  |  | 1 2 | F41.1 | Generalized anxiety disorder |  | 3,054 3,147 | 17.4% 17.9% | 0.193 | 0.014 |
|  |  | 1 2 | F30-F39 | Mood [affective] disorders |  | 8,378 8,435 | 47.6% 47.9% | 0.543 | 0.006 |
|  |  | 1 2 | F91 | Conduct disorders |  | 5,851 5,861 | 33.3% 33.3% | 0.910 | 0.001 |
|  |  | 1 2 | F42 | Obsessive-compulsive disorder |  | 627 657 | 3.6% 3.7% | 0.394 | 0.009 |
|  |  | 1 2 | F84 | Pervasive developmental disorders |  | 1,842 1,811 | 10.5% 10.3% | 0.588 | 0.006 |
|  |  | 1 2 | F51 | Sleep disorders not due to a substance or known physiological condition |  | 1,064 1,068 | 6.0% 6.1% | 0.929 | 0.001 |
|  |  | 1 2 | F50 | Eating disorders |  | 611 596 | 3.5% 3.4% | 0.660 | 0.005 |
|  |  | 1 2 | F10-F19 | Mental and behavioral disorders due to psychoactive substance use |  | 982 935 | 5.6% 5.3% | 0.270 | 0.012 |
|  |  | 1 2 | F20-F29 | Schizophrenia, schizotypal, delusional, and other non-mood psychotic disorders |  | 448 424 | 2.5% 2.4% | 0.411 | 0.009 |
|  |  | 1 2 | F70-F79 | Intellectual Disabilities |  | 499 434 | 2.8% 2.5% | 0.031 | 0.023 |
|  |  | 1 2 | F41.0 | Panic disorder [episodic paroxysmal anxiety] |  | 568 528 | 3.2% 3.0% | 0.220 | 0.013 |
|  |  | 1 2 | F32 | Depressive episode |  | 5,210 5,050 | 29.6% 28.7% | 0.061 | 0.020 |
|  |  | 1 2 | F33 | Major depressive disorder, recurrent |  | 2,044 2,042 | 11.6% 11.6% | 0.973 | <0.001 |
|  |  | 1 2 | F34.81 | Disruptive mood dysregulation disorder |  | 1,821 1,838 | 10.4% 10.4% | 0.767 | 0.003 |
|  |  | 1 2 | F31 | Bipolar disorder |  | 759 700 | 4.3% 4.0% | 0.115 | 0.017 |
|  |  | 1 2 | F60.3 | Borderline personality disorder |  | 382 347 | 2.2% 2.0% | 0.190 | 0.014 |
|  |  | 1 2 | F95 | Tic disorder |  | 464 409 | 2.6% 2.3% | 0.059 | 0.020 |

**SA6.4. Cohorts: Hispanic ADHD Cohort with PTSD (vs Hispanic ADHD Cohort without PTSD)**

### Query Criteria for Cohort 1 (query name: ADHD+PTSD_H)

This query was run on the network Research with 100 HCO(s) queried and 100 HCO(s) responded. A total of 57 provider(s) responded with patients. The final cohort included 3,507 patients who matched the query criteria listed in the table below.

| Cohort 1 | | | | | |
| --- | --- | --- | --- | --- | --- |
|  | must have |  | demographics | Age | Age (between 6 and 18 years (most recent occurrence)) |
|  |  | and | diagnosis | UMLS:ICD10CM:F90 | Attention-deficit hyperactivity disorders |
|  |  | and | diagnosis | UMLS:ICD10CM:F43.1 | Post-traumatic stress disorder (PTSD) |
|  |  | and | demographics | UMLS:HL7V3.0:Ethnicity:2135-2 | Hispanic or Latino |

### Query Criteria for Cohort 2 (query name: ADHD_H)

This query was run on the network Research with 100 HCO(s) queried and 100 HCO(s) responded. A total of 60 provider(s) responded with patients. The final cohort included 81,327 patients who matched the query criteria listed in the table below.

| Cohort 2 | | | | | |
| --- | --- | --- | --- | --- | --- |
|  | must have |  | demographics | Age | Age (between 6 and 18 years (most recent occurrence)) |
|  |  | and | diagnosis | UMLS:ICD10CM:F90 | Attention-deficit hyperactivity disorders |
|  |  | and | demographics | UMLS:HL7V3.0:Ethnicity:2135-2 | Hispanic or Latino |
|  | cannot have |  | diagnosis | UMLS:ICD10CM:F43.1 | Post-traumatic stress disorder (PTSD) |

Propensity Score Matching

| **Cohort 1 (N = 3,362) and cohort 2 (N = 3,362) characteristics after propensity score matching** | | | | | | | | | |
| --- | --- | --- | --- | --- | --- | --- | --- | --- | --- |
|  | **Demographics** | | | | | | | | |
|  |  | Cohort | |  | Mean ± SD | Patients | % of Cohort | P-Value | Std diff. |
|  |  | 1 2 | AI | Age at Index | 10.5 +/- 3.5 10.5 +/- 3.5 | 3,362 3,362 | 100% 100% | 0.843 | 0.005 |
|  |  | 1 2 | 2186-5 | Not Hispanic or Latino |  | 0 0 | 0% 0% | -- | -- |
|  |  | 1 2 | 2054-5 | Black or African American |  | 264 269 | 7.9% 8.0% | 0.821 | 0.006 |
|  |  | 1 2 | M | Male |  | 1,888 1,890 | 56.2% 56.2% | 0.961 | 0.001 |
|  | **Diagnosis** | | | | | | | | |
|  |  | Cohort | |  | Mean ± SD | Patients | % of Cohort | P-Value | Std diff. |
|  |  | 1 2 | F41.9 | Anxiety disorder, unspecified |  | 1,158 1,173 | 34.4% 34.9% | 0.701 | 0.009 |
|  |  | 1 2 | F41.1 | Generalized anxiety disorder |  | 517 503 | 15.4% 15.0% | 0.634 | 0.012 |
|  |  | 1 2 | F30-F39 | Mood [affective] disorders |  | 1,670 1,681 | 49.7% 50% | 0.788 | 0.007 |
|  |  | 1 2 | F91 | Conduct disorders |  | 1,058 1,074 | 31.5% 31.9% | 0.675 | 0.010 |
|  |  | 1 2 | F42 | Obsessive-compulsive disorder |  | 90 82 | 2.7% 2.4% | 0.537 | 0.015 |
|  |  | 1 2 | F84 | Pervasive developmental disorders |  | 359 317 | 10.7% 9.4% | 0.089 | 0.042 |
|  |  | 1 2 | F51 | Sleep disorders not due to a substance or known physiological condition |  | 213 211 | 6.3% 6.3% | 0.920 | 0.002 |
|  |  | 1 2 | F50 | Eating disorders |  | 145 126 | 4.3% 3.7% | 0.239 | 0.029 |
|  |  | 1 2 | F10-F19 | Mental and behavioral disorders due to psychoactive substance use |  | 257 241 | 7.6% 7.2% | 0.456 | 0.018 |
|  |  | 1 2 | F20-F29 | Schizophrenia, schizotypal, delusional, and other non-mood psychotic disorders |  | 109 99 | 3.2% 2.9% | 0.481 | 0.017 |
|  |  | 1 2 | F70-F79 | Intellectual Disabilities |  | 89 74 | 2.6% 2.2% | 0.234 | 0.029 |
|  |  | 1 2 | F41.0 | Panic disorder [episodic paroxysmal anxiety] |  | 105 92 | 3.1% 2.7% | 0.347 | 0.023 |
|  |  | 1 2 | F32 | Depressive episode |  | 1,081 1,031 | 32.2% 30.7% | 0.189 | 0.032 |
|  |  | 1 2 | F33 | Major depressive disorder, recurrent |  | 501 518 | 14.9% 15.4% | 0.563 | 0.014 |
|  |  | 1 2 | F34.81 | Disruptive mood dysregulation disorder |  | 333 350 | 9.9% 10.4% | 0.493 | 0.017 |
|  |  | 1 2 | F31 | Bipolar disorder |  | 126 119 | 3.7% 3.5% | 0.649 | 0.011 |
|  |  | 1 2 | F60.3 | Borderline personality disorder |  | 79 59 | 2.3% 1.8% | 0.085 | 0.042 |
|  |  | 1 2 | F95 | Tic disorder |  | 88 83 | 2.6% 2.5% | 0.699 | 0.009 |

**Cox Proportional Hazards Model**

**Supplement Analysis 7. Association Between Medication Type and Long-Term Outcomes in Individuals with ADHD and PTSD: Cox Proportional Hazards Models Adjusted for Demographics and Comorbidities**

**SA7.1. Cohorts: ADHD Medications (vs No ADHD Medications)**

### Query Criteria for Cohort 1 (query name: ADHD+PTSD+ADHD meds)

This query was run on the network Research with 104 HCO(s) queried and 104 HCO(s) responded. A total of 68 provider(s) responded with patients. The final cohort included 24,307 patients who matched the query criteria listed in the table below.

| Cohort 1 | | | | | |
| --- | --- | --- | --- | --- | --- |
|  | must have |  | demographics | Age | Age (between 6 and 18 years (most recent occurrence)) |
|  |  | and | diagnosis | UMLS:ICD10CM:F90 | Attention-deficit hyperactivity disorders |
|  |  | and | diagnosis | UMLS:ICD10CM:F43.1 | Post-traumatic stress disorder (PTSD) |
|  |  | and any of | medication | NLM:VA:CN802 | AMPHETAMINE LIKE STIMULANTS |
|  |  |  | medication | NLM:VA:CN801 | AMPHETAMINES |
|  |  |  | medication | NLM:RXNORM:2599 | clonidine |
|  |  |  | medication | NLM:RXNORM:11196 | viloxazine |
|  |  |  | medication | NLM:RXNORM:38400 | atomoxetine |
|  |  |  | medication | NLM:RXNORM:40114 | guanfacine |

### Query Criteria for Cohort 2 (query name: ADHD+PTSD_ADHD meds)

This query was run on the network Research with 104 HCO(s) queried and 104 HCO(s) responded. A total of 70 provider(s) responded with patients. The final cohort included 9,325 patients who matched the query criteria listed in the table below.

| Cohort 2 | | | | | |
| --- | --- | --- | --- | --- | --- |
|  | must have |  | demographics | Age | Age (between 6 and 18 years (most recent occurrence)) |
|  |  | and | diagnosis | UMLS:ICD10CM:F90 | Attention-deficit hyperactivity disorders |
|  |  | and | diagnosis | UMLS:ICD10CM:F43.1 | Post-traumatic stress disorder (PTSD) |
|  | cannot have |  | medication | NLM:VA:CN801 | AMPHETAMINES |
|  |  | or | medication | NLM:VA:CN802 | AMPHETAMINE LIKE STIMULANTS |
|  |  | or | medication | NLM:RXNORM:38400 | atomoxetine |
|  |  | or | medication | NLM:RXNORM:40114 | guanfacine |
|  |  | or | medication | NLM:RXNORM:2599 | clonidine |
|  |  | or | medication | NLM:RXNORM:11196 | viloxazine |

| **Cohort Information** | | |
| --- | --- | --- |
|  | **Cohort Name** | **Index Count** |
|  | ADHD+PTSD+ADHD meds | 25043 |
|  | ADHD+PTSD_ADHD meds | 9441 |

### Time Window

This analysis included outcomes that occurred in the time window that started 1 day after the first occurrence of the index event. Since no end date was specified all outcomes after the first occurrence of the index event were included.

**SA7.11: Hospital Inpatient and Observation Care Services**

| Outcome | | | | |
| --- | --- | --- | --- | --- |
|  | **Outcome definition** | | | |
|  | | Procedure | UMLS:CPT:1013659 | Hospital Inpatient and Observation Care Services |

| **Cox Model Results** | | | | | | | |
| --- | --- | --- | --- | --- | --- | --- | --- |
|  | **Covariate** | **Hazard Ratio** | **Coefficient** | **Standard Error** | **z** | **P > \|z\|** | **95% Confidence Interval** |
|  | Cohort 1 or Cohort 2 Membership | 1.466 | 0.382 | 0.030 | 12.547 | 0.000 | (1.381, 1.556) |
| F84 | Pervasive developmental disorders | 1.209 | 0.190 | 0.034 | 5.544 | 0.000 | (1.131, 1.293) |
| F20-F29 | Schizophrenia, schizotypal, delusional, and other non-mood psychotic disorders | 1.433 | 0.360 | 0.047 | 7.601 | 0.000 | (1.306, 1.572) |
| M | Male | 0.884 | -0.124 | 0.025 | -5.045 | 0.000 | (0.842, 0.927) |
| AI | Age at Index | 1.043 | 0.043 | 0.004 | 9.654 | 0.000 | (1.034, 1.052) |
| 2054-5 | Black or African American | 1.063 | 0.061 | 0.029 | 2.109 | 0.035 | (1.004, 1.126) |
| 2186-5 | Not Hispanic or Latino | 1.421 | 0.351 | 0.026 | 13.396 | 0.000 | (1.350, 1.496) |
| F41.9 | Anxiety disorder, unspecified | 0.981 | -0.020 | 0.025 | -0.798 | 0.425 | (0.934, 1.029) |
| F91 | Conduct disorders | 1.273 | 0.241 | 0.024 | 9.963 | 0.000 | (1.214, 1.335) |
| F41.1 | Generalized anxiety disorder | 0.999 | -0.001 | 0.029 | -0.045 | 0.964 | (0.944, 1.057) |
| F41.0 | Panic disorder [episodic paroxysmal anxiety] | 1.449 | 0.371 | 0.045 | 8.211 | 0.000 | (1.326, 1.582) |
| F42 | Obsessive-compulsive disorder | 1.175 | 0.162 | 0.048 | 3.336 | 0.001 | (1.069, 1.293) |
| F70-F79 | Intellectual Disabilities | 1.294 | 0.257 | 0.056 | 4.621 | 0.000 | (1.160, 1.443) |
| F50 | Eating disorders | 1.232 | 0.209 | 0.047 | 4.423 | 0.000 | (1.123, 1.351) |
| F51 | Sleep disorders not due to a substance or known physiological condition | 0.814 | -0.206 | 0.045 | -4.540 | 0.000 | (0.745, 0.890) |
| F10-F19 | Mental and behavioral disorders due to psychoactive substance use | 1.710 | 0.537 | 0.035 | 15.380 | 0 | (1.597, 1.831) |
| F60.3 | Borderline personality disorder | 1.816 | 0.597 | 0.050 | 12.032 | 0.000 | (1.648, 2.002) |
| F30-F39 | Mood [affective] disorders | 2.292 | 0.830 | 0.029 | 28.361 | 0 | (2.164, 2.427) |

**SA7.12: Emergency Department Services**

| Outcome | | | | |
| --- | --- | --- | --- | --- |
|  | **Outcome definition** | | | |
|  | | Procedure | UMLS:CPT:1013711 | Emergency Department Services |

| **Cox Model Results** | | | | | | | |
| --- | --- | --- | --- | --- | --- | --- | --- |
|  | **Covariate** | **Hazard Ratio** | **Coefficient** | **Standard Error** | **z** | **P > \|z\|** | **95% Confidence Interval** |
|  | Cohort 1 or Cohort 2 Membership | 1.161 | 0.149 | 0.024 | 6.144 | 0.000 | (1.107, 1.217) |
| F84 | Pervasive developmental disorders | 1.061 | 0.059 | 0.031 | 1.934 | 0.053 | (0.999, 1.127) |
| F20-F29 | Schizophrenia, schizotypal, delusional, and other non-mood psychotic disorders | 1.340 | 0.293 | 0.045 | 6.444 | 0.000 | (1.226, 1.465) |
| M | Male | 0.946 | -0.055 | 0.020 | -2.730 | 0.006 | (0.909, 0.985) |
| AI | Age at Index | 1.058 | 0.056 | 0.004 | 15.214 | 0 | (1.050, 1.065) |
| 2054-5 | Black or African American | 1.132 | 0.124 | 0.024 | 5.246 | 0.000 | (1.081, 1.186) |
| 2186-5 | Not Hispanic or Latino | 1.048 | 0.047 | 0.021 | 2.213 | 0.027 | (1.005, 1.092) |
| F41.9 | Anxiety disorder, unspecified | 1.145 | 0.135 | 0.021 | 6.523 | 0.000 | (1.099, 1.192) |
| F91 | Conduct disorders | 1.192 | 0.176 | 0.020 | 8.636 | 0.000 | (1.146, 1.241) |
| F41.1 | Generalized anxiety disorder | 0.827 | -0.190 | 0.026 | -7.213 | 0.000 | (0.785, 0.871) |
| F41.0 | Panic disorder [episodic paroxysmal anxiety] | 1.234 | 0.210 | 0.046 | 4.556 | 0.000 | (1.127, 1.350) |
| F42 | Obsessive-compulsive disorder | 0.972 | -0.028 | 0.048 | -0.585 | 0.558 | (0.885, 1.068) |
| F70-F79 | Intellectual Disabilities | 1.017 | 0.017 | 0.053 | 0.323 | 0.747 | (0.917, 1.129) |
| F50 | Eating disorders | 0.918 | -0.086 | 0.049 | -1.749 | 0.080 | (0.833, 1.010) |
| F51 | Sleep disorders not due to a substance or known physiological condition | 1.034 | 0.034 | 0.036 | 0.939 | 0.348 | (0.964, 1.110) |
| F10-F19 | Mental and behavioral disorders due to psychoactive substance use | 1.304 | 0.265 | 0.036 | 7.466 | 0.000 | (1.216, 1.397) |
| F60.3 | Borderline personality disorder | 1.271 | 0.240 | 0.053 | 4.525 | 0.000 | (1.146, 1.410) |
| F30-F39 | Mood [affective] disorders | 1.408 | 0.342 | 0.022 | 15.318 | 0 | (1.348, 1.471) |

**SA7.13: ANTIPSYCHOTICS**

| Outcome | | | | |
| --- | --- | --- | --- | --- |
|  | **Outcome definition** | | | |
|  | | Medication | NLM:VA:CN700 | ANTIPSYCHOTICS |

| **Cox Model Results** | | | | | | | |
| --- | --- | --- | --- | --- | --- | --- | --- |
|  | **Covariate** | **Hazard Ratio** | **Coefficient** | **Standard Error** | **z** | **P > \|z\|** | **95% Confidence Interval** |
|  | Cohort 1 or Cohort 2 Membership | 2.676 | 0.984 | 0.030 | 32.355 | 0 | (2.521, 2.841) |
| F84 | Pervasive developmental disorders | 1.455 | 0.375 | 0.027 | 13.779 | 0.000 | (1.379, 1.535) |
| F20-F29 | Schizophrenia, schizotypal, delusional, and other non-mood psychotic disorders | 2.112 | 0.747 | 0.038 | 19.583 | 0 | (1.959, 2.276) |
| M | Male | 1.005 | 0.005 | 0.020 | 0.267 | 0.790 | (0.967, 1.046) |
| AI | Age at Index | 1.012 | 0.012 | 0.004 | 3.263 | 0.001 | (1.005, 1.019) |
| 2054-5 | Black or African American | 1.032 | 0.031 | 0.024 | 1.300 | 0.194 | (0.984, 1.082) |
| 2186-5 | Not Hispanic or Latino | 0.930 | -0.073 | 0.020 | -3.564 | 0.000 | (0.893, 0.968) |
| F41.9 | Anxiety disorder, unspecified | 0.883 | -0.124 | 0.021 | -6.032 | 0.000 | (0.848, 0.920) |
| F91 | Conduct disorders | 1.451 | 0.373 | 0.020 | 18.860 | 0 | (1.396, 1.509) |
| F41.1 | Generalized anxiety disorder | 0.871 | -0.138 | 0.025 | -5.475 | 0.000 | (0.829, 0.915) |
| F41.0 | Panic disorder [episodic paroxysmal anxiety] | 1.019 | 0.019 | 0.046 | 0.421 | 0.674 | (0.932, 1.115) |
| F42 | Obsessive-compulsive disorder | 1.100 | 0.095 | 0.043 | 2.207 | 0.027 | (1.011, 1.197) |
| F70-F79 | Intellectual Disabilities | 1.432 | 0.359 | 0.044 | 8.158 | 0.000 | (1.313, 1.561) |
| F50 | Eating disorders | 1.005 | 0.005 | 0.045 | 0.114 | 0.909 | (0.920, 1.098) |
| F51 | Sleep disorders not due to a substance or known physiological condition | 0.957 | -0.043 | 0.035 | -1.230 | 0.219 | (0.893, 1.026) |
| F10-F19 | Mental and behavioral disorders due to psychoactive substance use | 1.437 | 0.362 | 0.033 | 11.134 | 0.000 | (1.348, 1.532) |
| F60.3 | Borderline personality disorder | 1.404 | 0.339 | 0.047 | 7.258 | 0.000 | (1.281, 1.538) |
| F30-F39 | Mood [affective] disorders | 2.457 | 0.899 | 0.023 | 38.767 | 0 | (2.348, 2.572) |

**SA7.14: Mood Stabilizers**

| Outcome | | | | |
| --- | --- | --- | --- | --- |
|  | **Outcome definition** | | | |
|  | | Medication | NLM:VA:CN750 | LITHIUM SALTS |
|  | | Medication | NLM:RXNORM:32624 | oxcarbazepine |
|  | | Medication | NLM:RXNORM:2002 | carbamazepine |
|  | | Medication | NLM:RXNORM:28439 | lamotrigine |
|  | | Medication | NLM:RXNORM:40254 | valproate |

| **Cox Model Results** | | | | | | | |
| --- | --- | --- | --- | --- | --- | --- | --- |
|  | **Covariate** | **Hazard Ratio** | **Coefficient** | **Standard Error** | **z** | **P > \|z\|** | **95% Confidence Interval** |
|  | Cohort 1 or Cohort 2 Membership | 2.650 | 0.974 | 0.052 | 18.625 | 0 | (2.391, 2.936) |
| F84 | Pervasive developmental disorders | 1.457 | 0.376 | 0.043 | 8.662 | 0.000 | (1.338, 1.586) |
| F20-F29 | Schizophrenia, schizotypal, delusional, and other non-mood psychotic disorders | 1.596 | 0.468 | 0.061 | 7.628 | 0.000 | (1.415, 1.800) |
| M | Male | 0.964 | -0.036 | 0.033 | -1.097 | 0.272 | (0.904, 1.029) |
| AI | Age at Index | 1.075 | 0.072 | 0.006 | 11.859 | 0.000 | (1.062, 1.088) |
| 2054-5 | Black or African American | 0.873 | -0.136 | 0.041 | -3.310 | 0.001 | (0.805, 0.946) |
| 2186-5 | Not Hispanic or Latino | 0.995 | -0.005 | 0.034 | -0.159 | 0.873 | (0.931, 1.062) |
| F41.9 | Anxiety disorder, unspecified | 0.904 | -0.101 | 0.033 | -3.008 | 0.003 | (0.847, 0.966) |
| F91 | Conduct disorders | 1.310 | 0.270 | 0.033 | 8.275 | 0.000 | (1.229, 1.396) |
| F41.1 | Generalized anxiety disorder | 0.867 | -0.143 | 0.040 | -3.527 | 0.000 | (0.801, 0.939) |
| F41.0 | Panic disorder [episodic paroxysmal anxiety] | 1.072 | 0.070 | 0.071 | 0.985 | 0.325 | (0.933, 1.231) |
| F42 | Obsessive-compulsive disorder | 1.135 | 0.127 | 0.067 | 1.904 | 0.057 | (0.996, 1.294) |
| F70-F79 | Intellectual Disabilities | 1.510 | 0.412 | 0.067 | 6.128 | 0.000 | (1.324, 1.723) |
| F50 | Eating disorders | 1.081 | 0.077 | 0.070 | 1.101 | 0.271 | (0.941, 1.240) |
| F51 | Sleep disorders not due to a substance or known physiological condition | 0.946 | -0.055 | 0.058 | -0.958 | 0.338 | (0.845, 1.060) |
| F10-F19 | Mental and behavioral disorders due to psychoactive substance use | 1.100 | 0.095 | 0.055 | 1.731 | 0.083 | (0.988, 1.224) |
| F60.3 | Borderline personality disorder | 1.574 | 0.453 | 0.070 | 6.518 | 0.000 | (1.373, 1.804) |
| F30-F39 | Mood [affective] disorders | 2.347 | 0.853 | 0.039 | 22.087 | 0 | (2.176, 2.531) |

**SA7.2. Cohorts: Antidepressants (vs. No Antidepressants)**

### Query Criteria for Cohort 1 (query name: PTSD_on AD)

This query was run on the network Research with 101 HCO(s) queried and 101 HCO(s) responded. A total of 66 provider(s) responded with patients. The final cohort included 19,065 patients who matched the query criteria listed in the table below.

| Cohort 1 | | | | | |
| --- | --- | --- | --- | --- | --- |
|  | must have |  | demographics | Age | Age (between 6 and 18 years (most recent occurrence)) |
|  |  | and | diagnosis | UMLS:ICD10CM:F90 | Attention-deficit hyperactivity disorders |
|  |  | and | diagnosis | UMLS:ICD10CM:F43.1 | Post-traumatic stress disorder (PTSD) |
|  |  | and | medication | NLM:VA:CN600 | ANTIDEPRESSANTS |

### Query Criteria for Cohort 2 (query name: PTSD_not on AD)

This query was run on the network Research with 101 HCO(s) queried and 101 HCO(s) responded. A total of 68 provider(s) responded with patients. The final cohort included 13,981 patients who matched the query criteria listed in the table below.

| Cohort 2 | | | | | |
| --- | --- | --- | --- | --- | --- |
|  | must have |  | demographics | Age | Age (between 6 and 18 years (most recent occurrence)) |
|  |  | and | diagnosis | UMLS:ICD10CM:F90 | Attention-deficit hyperactivity disorders |
|  |  | and | diagnosis | UMLS:ICD10CM:F43.1 | Post-traumatic stress disorder (PTSD) |
|  | cannot have |  | medication | NLM:VA:CN600 | ANTIDEPRESSANTS |

| **Cohort Information** | | |
| --- | --- | --- |
|  | **Cohort Name** | **Index Count** |
|  | PTSD_on AD | 19558 |
|  | PTSD_not on AD | 13870 |

**SA7.21: Hospital Inpatient and Observation Care Services**

| **Cox Model Results** | | | | | | | |
| --- | --- | --- | --- | --- | --- | --- | --- |
|  | **Covariate** | **Hazard Ratio** | **Coefficient** | **Standard Error** | **z** | **P > \|z\|** | **95% Confidence Interval** |
|  | Cohort 1 or Cohort 2 Membership | 1.938 | 0.662 | 0.031 | 21.159 | 0 | (1.823, 2.061) |
| F84 | Pervasive developmental disorders | 1.179 | 0.164 | 0.035 | 4.681 | 0.000 | (1.100, 1.263) |
| F20-F29 | Schizophrenia, schizotypal, delusional, and other non-mood psychotic disorders | 1.460 | 0.379 | 0.047 | 8.063 | 0.000 | (1.332, 1.601) |
| M | Male | 0.901 | -0.105 | 0.025 | -4.161 | 0.000 | (0.857, 0.946) |
| AI | Age at Index | 1.030 | 0.029 | 0.005 | 6.442 | 0.000 | (1.021, 1.039) |
| 2054-5 | Black or African American | 1.156 | 0.145 | 0.030 | 4.901 | 0.000 | (1.091, 1.225) |
| 2186-5 | Not Hispanic or Latino | 1.434 | 0.361 | 0.027 | 13.412 | 0.000 | (1.361, 1.512) |
| F41.9 | Anxiety disorder, unspecified | 0.946 | -0.055 | 0.025 | -2.214 | 0.027 | (0.901, 0.994) |
| F91 | Conduct disorders | 1.162 | 0.150 | 0.025 | 6.050 | 0.000 | (1.107, 1.220) |
| F41.1 | Generalized anxiety disorder | 0.966 | -0.035 | 0.029 | -1.219 | 0.223 | (0.913, 1.021) |
| F41.0 | Panic disorder [episodic paroxysmal anxiety] | 1.411 | 0.344 | 0.045 | 7.729 | 0.000 | (1.293, 1.540) |
| F42 | Obsessive-compulsive disorder | 1.123 | 0.116 | 0.049 | 2.368 | 0.018 | (1.020, 1.235) |
| F70-F79 | Intellectual Disabilities | 1.196 | 0.179 | 0.058 | 3.089 | 0.002 | (1.067, 1.339) |
| F50 | Eating disorders | 1.232 | 0.208 | 0.047 | 4.467 | 0.000 | (1.124, 1.349) |
| F51 | Sleep disorders not due to a substance or known physiological condition | 0.804 | -0.218 | 0.045 | -4.850 | 0.000 | (0.736, 0.878) |
| F10-F19 | Mental and behavioral disorders due to psychoactive substance use | 1.769 | 0.570 | 0.035 | 16.526 | 0 | (1.653, 1.893) |
| F60.3 | Borderline personality disorder | 1.917 | 0.651 | 0.049 | 13.163 | 0.000 | (1.740, 2.112) |
| F30-F39 | Mood [affective] disorders | 2.127 | 0.755 | 0.031 | 24.301 | 0 | (2.001, 2.260) |

**SA7.22: Emergency Department Services**

| **Cox Model Results** | | | | | | | |
| --- | --- | --- | --- | --- | --- | --- | --- |
|  | **Covariate** | **Hazard Ratio** | **Coefficient** | **Standard Error** | **z** | **P > \|z\|** | **95% Confidence Interval** |
|  | Cohort 1 or Cohort 2 Membership | 1.247 | 0.221 | 0.023 | 9.536 | 0.000 | (1.192, 1.305) |
| F84 | Pervasive developmental disorders | 1.047 | 0.046 | 0.031 | 1.483 | 0.138 | (0.985, 1.112) |
| F20-F29 | Schizophrenia, schizotypal, delusional, and other non-mood psychotic disorders | 1.302 | 0.264 | 0.046 | 5.797 | 0.000 | (1.191, 1.424) |
| M | Male | 0.962 | -0.039 | 0.021 | -1.899 | 0.058 | (0.924, 1.001) |
| AI | Age at Index | 1.044 | 0.043 | 0.004 | 11.604 | 0.000 | (1.037, 1.052) |
| 2054-5 | Black or African American | 1.158 | 0.147 | 0.024 | 6.126 | 0.000 | (1.105, 1.214) |
| 2186-5 | Not Hispanic or Latino | 1.021 | 0.020 | 0.021 | 0.966 | 0.334 | (0.979, 1.064) |
| F41.9 | Anxiety disorder, unspecified | 1.115 | 0.109 | 0.021 | 5.235 | 0.000 | (1.071, 1.162) |
| F91 | Conduct disorders | 1.215 | 0.195 | 0.021 | 9.496 | 0.000 | (1.167, 1.265) |
| F41.1 | Generalized anxiety disorder | 0.827 | -0.190 | 0.026 | -7.305 | 0.000 | (0.786, 0.870) |
| F41.0 | Panic disorder [episodic paroxysmal anxiety] | 1.183 | 0.168 | 0.046 | 3.682 | 0.000 | (1.082, 1.293) |
| F42 | Obsessive-compulsive disorder | 0.956 | -0.045 | 0.047 | -0.955 | 0.340 | (0.871, 1.049) |
| F70-F79 | Intellectual Disabilities | 1.038 | 0.038 | 0.052 | 0.720 | 0.472 | (0.937, 1.151) |
| F50 | Eating disorders | 0.937 | -0.065 | 0.048 | -1.339 | 0.180 | (0.853, 1.030) |
| F51 | Sleep disorders not due to a substance or known physiological condition | 1.002 | 0.002 | 0.036 | 0.057 | 0.955 | (0.934, 1.075) |
| F10-F19 | Mental and behavioral disorders due to psychoactive substance use | 1.275 | 0.243 | 0.035 | 6.859 | 0.000 | (1.189, 1.366) |
| F60.3 | Borderline personality disorder | 1.269 | 0.239 | 0.053 | 4.489 | 0.000 | (1.144, 1.409) |
| F30-F39 | Mood [affective] disorders | 1.376 | 0.319 | 0.023 | 13.825 | 0.000 | (1.315, 1.440) |

**SA7.23: Antipsychotics**

| **Cox Model Results** | | | | | | | |
| --- | --- | --- | --- | --- | --- | --- | --- |
|  | **Covariate** | **Hazard Ratio** | **Coefficient** | **Standard Error** | **z** | **P > \|z\|** | **95% Confidence Interval** |
|  | Cohort 1 or Cohort 2 Membership | 2.541 | 0.933 | 0.026 | 36.103 | 0 | (2.416, 2.673) |
| F84 | Pervasive developmental disorders | 1.451 | 0.373 | 0.027 | 13.558 | 0.000 | (1.375, 1.532) |
| F20-F29 | Schizophrenia, schizotypal, delusional, and other non-mood psychotic disorders | 2.061 | 0.723 | 0.038 | 19.078 | 0 | (1.914, 2.220) |
| M | Male | 1.085 | 0.081 | 0.021 | 3.961 | 0.000 | (1.042, 1.129) |
| AI | Age at Index | 0.984 | -0.017 | 0.004 | -4.482 | 0.000 | (0.977, 0.991) |
| 2054-5 | Black or African American | 1.100 | 0.095 | 0.025 | 3.879 | 0.000 | (1.048, 1.154) |
| 2186-5 | Not Hispanic or Latino | 0.932 | -0.070 | 0.021 | -3.391 | 0.001 | (0.895, 0.971) |
| F41.9 | Anxiety disorder, unspecified | 0.829 | -0.188 | 0.021 | -9.120 | 0.000 | (0.796, 0.863) |
| F91 | Conduct disorders | 1.460 | 0.378 | 0.020 | 18.732 | 0 | (1.403, 1.519) |
| F41.1 | Generalized anxiety disorder | 0.834 | -0.182 | 0.025 | -7.277 | 0.000 | (0.794, 0.876) |
| F41.0 | Panic disorder [episodic paroxysmal anxiety] | 1.001 | 0.001 | 0.045 | 0.026 | 0.979 | (0.917, 1.093) |
| F42 | Obsessive-compulsive disorder | 1.051 | 0.049 | 0.043 | 1.145 | 0.252 | (0.965, 1.143) |
| F70-F79 | Intellectual Disabilities | 1.400 | 0.336 | 0.044 | 7.594 | 0.000 | (1.283, 1.527) |
| F50 | Eating disorders | 1.042 | 0.041 | 0.044 | 0.937 | 0.349 | (0.956, 1.137) |
| F51 | Sleep disorders not due to a substance or known physiological condition | 0.929 | -0.073 | 0.035 | -2.086 | 0.037 | (0.868, 0.996) |
| F10-F19 | Mental and behavioral disorders due to psychoactive substance use | 1.466 | 0.382 | 0.032 | 11.833 | 0.000 | (1.376, 1.562) |
| F60.3 | Borderline personality disorder | 1.429 | 0.357 | 0.047 | 7.622 | 0.000 | (1.304, 1.566) |
| F30-F39 | Mood [affective] disorders | 2.124 | 0.753 | 0.024 | 31.033 | 0 | (2.025, 2.227) |

**SA7.24: Mood Stabilizers**

| **Cox Model Results** | | | | | | | |
| --- | --- | --- | --- | --- | --- | --- | --- |
|  | **Covariate** | **Hazard Ratio** | **Coefficient** | **Standard Error** | **z** | **P > \|z\|** | **95% Confidence Interval** |
|  | Cohort 1 or Cohort 2 Membership | 2.229 | 0.802 | 0.044 | 18.298 | 0 | (2.046, 2.429) |
| F84 | Pervasive developmental disorders | 1.444 | 0.368 | 0.044 | 8.336 | 0.000 | (1.325, 1.575) |
| F20-F29 | Schizophrenia, schizotypal, delusional, and other non-mood psychotic disorders | 1.589 | 0.463 | 0.061 | 7.570 | 0.000 | (1.410, 1.792) |
| M | Male | 1.030 | 0.030 | 0.034 | 0.887 | 0.375 | (0.964, 1.101) |
| AI | Age at Index | 1.054 | 0.053 | 0.006 | 8.436 | 0.000 | (1.041, 1.067) |
| 2054-5 | Black or African American | 0.919 | -0.085 | 0.042 | -2.018 | 0.044 | (0.846, 0.998) |
| 2186-5 | Not Hispanic or Latino | 1.013 | 0.012 | 0.034 | 0.362 | 0.717 | (0.946, 1.083) |
| F41.9 | Anxiety disorder, unspecified | 0.866 | -0.144 | 0.033 | -4.287 | 0.000 | (0.811, 0.925) |
| F91 | Conduct disorders | 1.354 | 0.303 | 0.033 | 9.080 | 0.000 | (1.268, 1.445) |
| F41.1 | Generalized anxiety disorder | 0.852 | -0.161 | 0.040 | -4.017 | 0.000 | (0.788, 0.921) |
| F41.0 | Panic disorder [episodic paroxysmal anxiety] | 1.033 | 0.032 | 0.070 | 0.465 | 0.642 | (0.901, 1.184) |
| F42 | Obsessive-compulsive disorder | 1.111 | 0.105 | 0.066 | 1.581 | 0.114 | (0.975, 1.265) |
| F70-F79 | Intellectual Disabilities | 1.576 | 0.455 | 0.067 | 6.793 | 0.000 | (1.382, 1.797) |
| F50 | Eating disorders | 1.117 | 0.110 | 0.069 | 1.600 | 0.110 | (0.976, 1.278) |
| F51 | Sleep disorders not due to a substance or known physiological condition | 0.960 | -0.041 | 0.057 | -0.718 | 0.473 | (0.859, 1.073) |
| F10-F19 | Mental and behavioral disorders due to psychoactive substance use | 1.106 | 0.101 | 0.054 | 1.854 | 0.064 | (0.994, 1.231) |
| F60.3 | Borderline personality disorder | 1.501 | 0.406 | 0.072 | 5.673 | 0.000 | (1.304, 1.727) |
| F30-F39 | Mood [affective] disorders | 2.091 | 0.738 | 0.041 | 18.044 | 0 | (1.930, 2.265) |

**SA7.3. Cohorts: ADHD Medication (vs. Antidepressants)**

### Query Criteria for Cohort 1 (query name: ADHD+PTSD+ADHD meds+NoAD)

This query was run on the network Research with 104 HCO(s) queried and 104 HCO(s) responded. A total of 66 provider(s) responded with patients. The final cohort included 7,700 patients who matched the query criteria listed in the table below.

| Cohort 1 | | | | | |
| --- | --- | --- | --- | --- | --- |
|  | must have |  | demographics | Age | Age (between 6 and 18 years (most recent occurrence)) |
|  |  | and | diagnosis | UMLS:ICD10CM:F90 | Attention-deficit hyperactivity disorders |
|  |  | and | diagnosis | UMLS:ICD10CM:F43.1 | Post-traumatic stress disorder (PTSD) |
|  |  | and any of | medication | NLM:VA:CN802 | AMPHETAMINE LIKE STIMULANTS |
|  |  |  | medication | NLM:VA:CN801 | AMPHETAMINES |
|  |  |  | medication | NLM:RXNORM:2599 | clonidine |
|  |  |  | medication | NLM:RXNORM:11196 | viloxazine |
|  |  |  | medication | NLM:RXNORM:38400 | atomoxetine |
|  |  |  | medication | NLM:RXNORM:40114 | guanfacine |
|  | cannot have |  | medication | NLM:VA:CN600 | ANTIDEPRESSANTS |

### Query Criteria for Cohort 2 (query name: ADHD+PTSD+AD + No ADHD meds)

This query was run on the network Research with 104 HCO(s) queried and 104 HCO(s) responded. A total of 66 provider(s) responded with patients. The final cohort included 2,733 patients who matched the query criteria listed in the table below.

| Cohort 2 | | | | | |
| --- | --- | --- | --- | --- | --- |
|  | must have |  | demographics | Age | Age (between 6 and 18 years (most recent occurrence)) |
|  |  | and | diagnosis | UMLS:ICD10CM:F90 | Attention-deficit hyperactivity disorders |
|  |  | and | diagnosis | UMLS:ICD10CM:F43.1 | Post-traumatic stress disorder (PTSD) |
|  |  | and | medication | NLM:VA:CN600 | ANTIDEPRESSANTS |
|  | cannot have |  | medication | NLM:VA:CN801 | AMPHETAMINES |
|  |  | or | medication | NLM:VA:CN802 | AMPHETAMINE LIKE STIMULANTS |
|  |  | or | medication | NLM:RXNORM:38400 | atomoxetine |
|  |  | or | medication | NLM:RXNORM:40114 | guanfacine |
|  |  | or | medication | NLM:RXNORM:2599 | clonidine |
|  |  | or | medication | NLM:RXNORM:11196 | viloxazine |

| **Cohort Information** | | |
| --- | --- | --- |
|  | **Cohort Name** | **Index Count** |
|  | ADHD+PTSD+ADHD meds+NoAD | 7401 |
|  | ADHD+PTSD+AD + No ADHD meds | 2706 |

**SA7.31: Hospital Inpatient and Observation Care Services**

| **Cox Model Results** | | | | | | | |
| --- | --- | --- | --- | --- | --- | --- | --- |
|  | **Covariate** | **Hazard Ratio** | **Coefficient** | **Standard Error** | **z** | **P > \|z\|** | **95% Confidence Interval** |
|  | Cohort 1 or Cohort 2 Membership | 0.617 | -0.483 | 0.062 | -7.850 | 0.000 | (0.547, 0.696) |
| F84 | Pervasive developmental disorders | 1.216 | 0.196 | 0.071 | 2.752 | 0.006 | (1.058, 1.398) |
| F20-F29 | Schizophrenia, schizotypal, delusional, and other non-mood psychotic disorders | 1.573 | 0.453 | 0.090 | 5.024 | 0.000 | (1.318, 1.878) |
| M | Male | 0.931 | -0.072 | 0.051 | -1.418 | 0.156 | (0.842, 1.028) |
| AI | Age at Index | 1.018 | 0.018 | 0.009 | 1.969 | 0.049 | (1.000, 1.036) |
| 2054-5 | Black or African American | 1.016 | 0.016 | 0.056 | 0.280 | 0.780 | (0.910, 1.133) |
| 2186-5 | Not Hispanic or Latino | 1.384 | 0.325 | 0.051 | 6.391 | 0.000 | (1.253, 1.529) |
| F41.9 | Anxiety disorder, unspecified | 0.942 | -0.060 | 0.051 | -1.189 | 0.234 | (0.853, 1.040) |
| F91 | Conduct disorders | 1.273 | 0.242 | 0.049 | 4.882 | 0.000 | (1.156, 1.403) |
| F41.1 | Generalized anxiety disorder | 1.150 | 0.139 | 0.059 | 2.346 | 0.019 | (1.023, 1.292) |
| F41.0 | Panic disorder [episodic paroxysmal anxiety] | 1.347 | 0.298 | 0.091 | 3.291 | 0.001 | (1.128, 1.609) |
| F42 | Obsessive-compulsive disorder | 1.096 | 0.091 | 0.107 | 0.850 | 0.395 | (0.888, 1.352) |
| F70-F79 | Intellectual Disabilities | 1.160 | 0.149 | 0.126 | 1.182 | 0.237 | (0.907, 1.485) |
| F50 | Eating disorders | 1.196 | 0.179 | 0.093 | 1.926 | 0.054 | (0.997, 1.435) |
| F51 | Sleep disorders not due to a substance or known physiological condition | 0.882 | -0.125 | 0.093 | -1.338 | 0.181 | (0.735, 1.060) |
| F10-F19 | Mental and behavioral disorders due to psychoactive substance use | 1.720 | 0.542 | 0.067 | 8.037 | 0.000 | (1.507, 1.963) |
| F60.3 | Borderline personality disorder | 1.693 | 0.526 | 0.103 | 5.086 | 0.000 | (1.382, 2.073) |
| F30-F39 | Mood [affective] disorders | 2.297 | 0.832 | 0.059 | 14.013 | 0.000 | (2.045, 2.580) |

**SA7.32: Emergency Department Services**

| **Cox Model Results** | | | | | | | |
| --- | --- | --- | --- | --- | --- | --- | --- |
|  | **Covariate** | **Hazard Ratio** | **Coefficient** | **Standard Error** | **z** | **P > \|z\|** | **95% Confidence Interval** |
|  | Cohort 1 or Cohort 2 Membership | 0.834 | -0.182 | 0.055 | -3.332 | 0.001 | (0.749, 0.928) |
| F84 | Pervasive developmental disorders | 1.058 | 0.057 | 0.064 | 0.893 | 0.372 | (0.934, 1.199) |
| F20-F29 | Schizophrenia, schizotypal, delusional, and other non-mood psychotic disorders | 0.958 | -0.043 | 0.105 | -0.410 | 0.682 | (0.781, 1.176) |
| M | Male | 0.948 | -0.054 | 0.041 | -1.307 | 0.191 | (0.875, 1.027) |
| AI | Age at Index | 1.050 | 0.049 | 0.007 | 6.707 | 0.000 | (1.035, 1.065) |
| 2054-5 | Black or African American | 1.237 | 0.212 | 0.043 | 4.918 | 0.000 | (1.136, 1.346) |
| 2186-5 | Not Hispanic or Latino | 0.984 | -0.016 | 0.041 | -0.406 | 0.685 | (0.909, 1.065) |
| F41.9 | Anxiety disorder, unspecified | 1.085 | 0.081 | 0.043 | 1.895 | 0.058 | (0.997, 1.180) |
| F91 | Conduct disorders | 1.281 | 0.248 | 0.040 | 6.210 | 0.000 | (1.185, 1.386) |
| F41.1 | Generalized anxiety disorder | 0.849 | -0.164 | 0.058 | -2.819 | 0.005 | (0.758, 0.951) |
| F41.0 | Panic disorder [episodic paroxysmal anxiety] | 1.082 | 0.079 | 0.104 | 0.761 | 0.447 | (0.883, 1.328) |
| F42 | Obsessive-compulsive disorder | 0.938 | -0.064 | 0.115 | -0.555 | 0.579 | (0.748, 1.176) |
| F70-F79 | Intellectual Disabilities | 0.914 | -0.090 | 0.114 | -0.790 | 0.429 | (0.730, 1.143) |
| F50 | Eating disorders | 0.844 | -0.169 | 0.102 | -1.663 | 0.096 | (0.692, 1.031) |
| F51 | Sleep disorders not due to a substance or known physiological condition | 1.019 | 0.018 | 0.075 | 0.247 | 0.805 | (0.880, 1.179) |
| F10-F19 | Mental and behavioral disorders due to psychoactive substance use | 1.289 | 0.254 | 0.070 | 3.610 | 0.000 | (1.123, 1.479) |
| F60.3 | Borderline personality disorder | 1.108 | 0.103 | 0.118 | 0.872 | 0.383 | (0.880, 1.396) |
| F30-F39 | Mood [affective] disorders | 1.488 | 0.398 | 0.045 | 8.922 | 0.000 | (1.364, 1.624) |

**SA7.33: Antipsychotics**

| **Cox Model Results** | | | | | | | |
| --- | --- | --- | --- | --- | --- | --- | --- |
|  | **Covariate** | **Hazard Ratio** | **Coefficient** | **Standard Error** | **z** | **P > \|z\|** | **95% Confidence Interval** |
|  | Cohort 1 or Cohort 2 Membership | 0.952 | -0.050 | 0.055 | -0.900 | 0.368 | (0.854, 1.060) |
| F84 | Pervasive developmental disorders | 1.558 | 0.443 | 0.057 | 7.834 | 0.000 | (1.394, 1.740) |
| F20-F29 | Schizophrenia, schizotypal, delusional, and other non-mood psychotic disorders | 2.294 | 0.830 | 0.076 | 10.975 | 0.000 | (1.978, 2.661) |
| M | Male | 1.086 | 0.083 | 0.044 | 1.893 | 0.058 | (0.997, 1.183) |
| AI | Age at Index | 0.991 | -0.009 | 0.007 | -1.188 | 0.235 | (0.977, 1.006) |
| 2054-5 | Black or African American | 1.044 | 0.043 | 0.047 | 0.915 | 0.360 | (0.952, 1.146) |
| 2186-5 | Not Hispanic or Latino | 0.869 | -0.140 | 0.042 | -3.356 | 0.001 | (0.801, 0.943) |
| F41.9 | Anxiety disorder, unspecified | 0.839 | -0.176 | 0.045 | -3.870 | 0.000 | (0.767, 0.917) |
| F91 | Conduct disorders | 1.514 | 0.415 | 0.042 | 9.986 | 0.000 | (1.396, 1.643) |
| F41.1 | Generalized anxiety disorder | 0.805 | -0.217 | 0.060 | -3.641 | 0.000 | (0.716, 0.905) |
| F41.0 | Panic disorder [episodic paroxysmal anxiety] | 1.213 | 0.193 | 0.098 | 1.972 | 0.049 | (1.001, 1.470) |
| F42 | Obsessive-compulsive disorder | 1.100 | 0.095 | 0.104 | 0.912 | 0.362 | (0.896, 1.350) |
| F70-F79 | Intellectual Disabilities | 1.479 | 0.391 | 0.096 | 4.084 | 0.000 | (1.226, 1.784) |
| F50 | Eating disorders | 1.210 | 0.191 | 0.091 | 2.090 | 0.037 | (1.012, 1.447) |
| F51 | Sleep disorders not due to a substance or known physiological condition | 0.854 | -0.157 | 0.082 | -1.928 | 0.054 | (0.728, 1.003) |
| F10-F19 | Mental and behavioral disorders due to psychoactive substance use | 1.502 | 0.406 | 0.066 | 6.121 | 0.000 | (1.318, 1.710) |
| F60.3 | Borderline personality disorder | 1.345 | 0.296 | 0.102 | 2.912 | 0.004 | (1.102, 1.642) |
| F30-F39 | Mood [affective] disorders | 3.042 | 1.113 | 0.049 | 22.882 | 0 | (2.766, 3.346) |

**SA7.34: Mood Stabilizers**

| **Cox Model Results** | | | | | | | |
| --- | --- | --- | --- | --- | --- | --- | --- |
|  | **Covariate** | **Hazard Ratio** | **Coefficient** | **Standard Error** | **z** | **P > \|z\|** | **95% Confidence Interval** |
|  | Cohort 1 or Cohort 2 Membership | 1.320 | 0.278 | 0.096 | 2.888 | 0.004 | (1.093, 1.594) |
| F84 | Pervasive developmental disorders | 1.246 | 0.220 | 0.105 | 2.101 | 0.036 | (1.015, 1.529) |
| F20-F29 | Schizophrenia, schizotypal, delusional, and other non-mood psychotic disorders | 2.118 | 0.750 | 0.127 | 5.923 | 0.000 | (1.652, 2.715) |
| M | Male | 0.941 | -0.060 | 0.077 | -0.788 | 0.430 | (0.810, 1.094) |
| AI | Age at Index | 1.065 | 0.063 | 0.013 | 4.763 | 0.000 | (1.038, 1.093) |
| 2054-5 | Black or African American | 0.790 | -0.236 | 0.087 | -2.703 | 0.007 | (0.666, 0.937) |
| 2186-5 | Not Hispanic or Latino | 1.049 | 0.048 | 0.074 | 0.647 | 0.518 | (0.907, 1.214) |
| F41.9 | Anxiety disorder, unspecified | 0.892 | -0.114 | 0.079 | -1.444 | 0.149 | (0.765, 1.042) |
| F91 | Conduct disorders | 1.138 | 0.129 | 0.075 | 1.721 | 0.085 | (0.982, 1.318) |
| F41.1 | Generalized anxiety disorder | 0.817 | -0.202 | 0.103 | -1.961 | 0.050 | (0.668, 1.000) |
| F41.0 | Panic disorder [episodic paroxysmal anxiety] | 0.660 | -0.415 | 0.205 | -2.022 | 0.043 | (0.441, 0.987) |
| F42 | Obsessive-compulsive disorder | 1.305 | 0.266 | 0.168 | 1.582 | 0.114 | (0.938, 1.815) |
| F70-F79 | Intellectual Disabilities | 1.887 | 0.635 | 0.149 | 4.274 | 0.000 | (1.410, 2.524) |
| F50 | Eating disorders | 1.059 | 0.057 | 0.168 | 0.340 | 0.734 | (0.761, 1.473) |
| F51 | Sleep disorders not due to a substance or known physiological condition | 1.010 | 0.010 | 0.139 | 0.069 | 0.945 | (0.769, 1.326) |
| F10-F19 | Mental and behavioral disorders due to psychoactive substance use | 1.160 | 0.149 | 0.123 | 1.214 | 0.225 | (0.913, 1.476) |
| F60.3 | Borderline personality disorder | 1.052 | 0.051 | 0.195 | 0.260 | 0.795 | (0.718, 1.541) |
| F30-F39 | Mood [affective] disorders | 3.423 | 1.230 | 0.087 | 14.116 | 0.000 | (2.885, 4.060) |

**SA7.4. Cohorts: CNS stimulants (vs. Antidepressants)**

### Query Criteria for Cohort 1 (query name: PTSD_onST_Not on AD)

This query was run on the network Research with 101 HCO(s) queried and 101 HCO(s) responded. A total of 63 provider(s) responded with patients. The final cohort included 5,809 patients who matched the query criteria listed in the table below.

| Cohort 1 | | | | | |
| --- | --- | --- | --- | --- | --- |
|  | must have |  | demographics | Age | Age (between 6 and 18 years (most recent occurrence)) |
|  |  | and | diagnosis | UMLS:ICD10CM:F90 | Attention-deficit hyperactivity disorders |
|  |  | and | diagnosis | UMLS:ICD10CM:F43.1 | Post-traumatic stress disorder (PTSD) |
|  |  | and any of | medication | NLM:VA:CN801 | AMPHETAMINES |
|  |  |  | medication | NLM:VA:CN802 | AMPHETAMINE LIKE STIMULANTS |
|  | cannot have |  | medication | NLM:VA:CN600 | ANTIDEPRESSANTS |

### Query Criteria for Cohort 2 (query name: PTSD_onAD_Not on ST)

This query was run on the network Research with 101 HCO(s) queried and 101 HCO(s) responded. A total of 64 provider(s) responded with patients. The final cohort included 6,130 patients who matched the query criteria listed in the table below.

| Cohort 2 | | | | | |
| --- | --- | --- | --- | --- | --- |
|  | must have |  | demographics | Age | Age (between 6 and 18 years (most recent occurrence)) |
|  |  | and | diagnosis | UMLS:ICD10CM:F90 | Attention-deficit hyperactivity disorders |
|  |  | and | diagnosis | UMLS:ICD10CM:F43.1 | Post-traumatic stress disorder (PTSD) |
|  |  | and | medication | NLM:VA:CN600 | ANTIDEPRESSANTS |
|  | cannot have |  | medication | NLM:VA:CN801 | AMPHETAMINES |
|  |  | or | medication | NLM:VA:CN802 | AMPHETAMINE LIKE STIMULANTS |

| **Cohort Information** | | |
| --- | --- | --- |
|  | **Cohort Name** | **Index Count** |
|  | PTSD_onST_Not on AD | 5712 |
|  | PTSD_onAD_Not on ST | 6199 |

**SA7.41: Hospital Inpatient and Observation Care Services**

| **Cox Model Results** | | | | | | | |
| --- | --- | --- | --- | --- | --- | --- | --- |
|  | **Covariate** | **Hazard Ratio** | **Coefficient** | **Standard Error** | **z** | **P > \|z\|** | **95% Confidence Interval** |
|  | Cohort 1 or Cohort 2 Membership | 0.527 | -0.641 | 0.052 | -12.258 | 0.000 | (0.475, 0.584) |
| F84 | Pervasive developmental disorders | 1.163 | 0.151 | 0.058 | 2.629 | 0.009 | (1.039, 1.302) |
| F20-F29 | Schizophrenia, schizotypal, delusional, and other non-mood psychotic disorders | 1.429 | 0.357 | 0.069 | 5.182 | 0.000 | (1.248, 1.635) |
| M | Male | 0.887 | -0.119 | 0.042 | -2.835 | 0.005 | (0.817, 0.964) |
| AI | Age at Index | 1.016 | 0.016 | 0.008 | 2.105 | 0.035 | (1.001, 1.032) |
| 2054-5 | Black or African American | 1.088 | 0.084 | 0.049 | 1.736 | 0.083 | (0.989, 1.197) |
| 2186-5 | Not Hispanic or Latino | 1.399 | 0.336 | 0.042 | 7.949 | 0.000 | (1.288, 1.520) |
| F41.9 | Anxiety disorder, unspecified | 0.942 | -0.059 | 0.041 | -1.458 | 0.145 | (0.870, 1.021) |
| F91 | Conduct disorders | 1.193 | 0.176 | 0.041 | 4.334 | 0.000 | (1.101, 1.292) |
| F41.1 | Generalized anxiety disorder | 1.091 | 0.087 | 0.045 | 1.912 | 0.056 | (0.998, 1.193) |
| F41.0 | Panic disorder [episodic paroxysmal anxiety] | 1.275 | 0.243 | 0.068 | 3.575 | 0.000 | (1.116, 1.457) |
| F42 | Obsessive-compulsive disorder | 1.208 | 0.189 | 0.076 | 2.493 | 0.013 | (1.041, 1.402) |
| F70-F79 | Intellectual Disabilities | 1.115 | 0.109 | 0.103 | 1.060 | 0.289 | (0.912, 1.364) |
| F50 | Eating disorders | 1.252 | 0.225 | 0.070 | 3.227 | 0.001 | (1.092, 1.435) |
| F51 | Sleep disorders not due to a substance or known physiological condition | 0.773 | -0.258 | 0.076 | -3.373 | 0.001 | (0.665, 0.898) |
| F10-F19 | Mental and behavioral disorders due to psychoactive substance use | 1.564 | 0.447 | 0.053 | 8.426 | 0.000 | (1.410, 1.736) |
| F60.3 | Borderline personality disorder | 1.827 | 0.603 | 0.077 | 7.819 | 0.000 | (1.571, 2.125) |
| F30-F39 | Mood [affective] disorders | 2.202 | 0.789 | 0.054 | 14.620 | 0 | (1.981, 2.447) |

**SA7.42: Emergency Department Services**

| **Cox Model Results** | | | | | | | |
| --- | --- | --- | --- | --- | --- | --- | --- |
|  | **Covariate** | **Hazard Ratio** | **Coefficient** | **Standard Error** | **z** | **P > \|z\|** | **95% Confidence Interval** |
|  | Cohort 1 or Cohort 2 Membership | 0.740 | -0.302 | 0.043 | -7.049 | 0.000 | (0.680, 0.804) |
| F84 | Pervasive developmental disorders | 1.001 | 0.001 | 0.055 | 0.024 | 0.981 | (0.899, 1.115) |
| F20-F29 | Schizophrenia, schizotypal, delusional, and other non-mood psychotic disorders | 1.126 | 0.119 | 0.074 | 1.601 | 0.109 | (0.974, 1.303) |
| M | Male | 0.916 | -0.088 | 0.036 | -2.426 | 0.015 | (0.853, 0.983) |
| AI | Age at Index | 1.055 | 0.054 | 0.007 | 8.008 | 0.000 | (1.041, 1.069) |
| 2054-5 | Black or African American | 1.154 | 0.144 | 0.041 | 3.535 | 0.000 | (1.066, 1.250) |
| 2186-5 | Not Hispanic or Latino | 1.031 | 0.031 | 0.036 | 0.847 | 0.397 | (0.961, 1.106) |
| F41.9 | Anxiety disorder, unspecified | 1.080 | 0.077 | 0.037 | 2.107 | 0.035 | (1.005, 1.161) |
| F91 | Conduct disorders | 1.259 | 0.230 | 0.035 | 6.499 | 0.000 | (1.174, 1.349) |
| F41.1 | Generalized anxiety disorder | 0.821 | -0.197 | 0.045 | -4.330 | 0.000 | (0.751, 0.898) |
| F41.0 | Panic disorder [episodic paroxysmal anxiety] | 1.124 | 0.117 | 0.074 | 1.568 | 0.117 | (0.971, 1.300) |
| F42 | Obsessive-compulsive disorder | 0.926 | -0.077 | 0.085 | -0.898 | 0.369 | (0.784, 1.095) |
| F70-F79 | Intellectual Disabilities | 0.938 | -0.064 | 0.098 | -0.649 | 0.516 | (0.774, 1.137) |
| F50 | Eating disorders | 0.885 | -0.122 | 0.079 | -1.556 | 0.120 | (0.759, 1.032) |
| F51 | Sleep disorders not due to a substance or known physiological condition | 0.959 | -0.041 | 0.064 | -0.648 | 0.517 | (0.846, 1.088) |
| F10-F19 | Mental and behavioral disorders due to psychoactive substance use | 1.177 | 0.163 | 0.057 | 2.878 | 0.004 | (1.053, 1.316) |
| F60.3 | Borderline personality disorder | 1.014 | 0.014 | 0.096 | 0.142 | 0.887 | (0.839, 1.225) |
| F30-F39 | Mood [affective] disorders | 1.418 | 0.349 | 0.042 | 8.320 | 0.000 | (1.306, 1.539) |

**SA7.43: Antipsychotics**

| **Cox Model Results** | | | | | | | |
| --- | --- | --- | --- | --- | --- | --- | --- |
|  | **Covariate** | **Hazard Ratio** | **Coefficient** | **Standard Error** | **z** | **P > \|z\|** | **95% Confidence Interval** |
|  | Cohort 1 or Cohort 2 Membership | 0.578 | -0.548 | 0.042 | -12.910 | 0.000 | (0.532, 0.628) |
| F84 | Pervasive developmental disorders | 1.519 | 0.418 | 0.046 | 9.007 | 0.000 | (1.387, 1.663) |
| F20-F29 | Schizophrenia, schizotypal, delusional, and other non-mood psychotic disorders | 1.977 | 0.681 | 0.058 | 11.729 | 0.000 | (1.764, 2.215) |
| M | Male | 1.056 | 0.054 | 0.036 | 1.517 | 0.129 | (0.984, 1.132) |
| AI | Age at Index | 0.989 | -0.011 | 0.006 | -1.670 | 0.095 | (0.977, 1.002) |
| 2054-5 | Black or African American | 1.093 | 0.089 | 0.041 | 2.154 | 0.031 | (1.008, 1.185) |
| 2186-5 | Not Hispanic or Latino | 0.927 | -0.076 | 0.035 | -2.186 | 0.029 | (0.866, 0.992) |
| F41.9 | Anxiety disorder, unspecified | 0.839 | -0.175 | 0.036 | -4.882 | 0.000 | (0.783, 0.901) |
| F91 | Conduct disorders | 1.477 | 0.390 | 0.034 | 11.301 | 0.000 | (1.380, 1.580) |
| F41.1 | Generalized anxiety disorder | 0.807 | -0.214 | 0.043 | -4.968 | 0.000 | (0.742, 0.878) |
| F41.0 | Panic disorder [episodic paroxysmal anxiety] | 0.939 | -0.063 | 0.072 | -0.879 | 0.379 | (0.816, 1.080) |
| F42 | Obsessive-compulsive disorder | 1.094 | 0.090 | 0.073 | 1.221 | 0.222 | (0.947, 1.263) |
| F70-F79 | Intellectual Disabilities | 1.453 | 0.374 | 0.078 | 4.802 | 0.000 | (1.247, 1.692) |
| F50 | Eating disorders | 1.136 | 0.127 | 0.069 | 1.833 | 0.067 | (0.991, 1.301) |
| F51 | Sleep disorders not due to a substance or known physiological condition | 0.921 | -0.082 | 0.062 | -1.320 | 0.187 | (0.816, 1.041) |
| F10-F19 | Mental and behavioral disorders due to psychoactive substance use | 1.379 | 0.322 | 0.051 | 6.352 | 0.000 | (1.249, 1.523) |
| F60.3 | Borderline personality disorder | 1.440 | 0.365 | 0.076 | 4.779 | 0.000 | (1.240, 1.672) |
| F30-F39 | Mood [affective] disorders | 2.543 | 0.933 | 0.044 | 21.010 | 0 | (2.331, 2.774) |

**SA7.44: Mood Stabilizers**

| **Cox Model Results** | | | | | | | |
| --- | --- | --- | --- | --- | --- | --- | --- |
|  | **Covariate** | **Hazard Ratio** | **Coefficient** | **Standard Error** | **z** | **P > \|z\|** | **95% Confidence Interval** |
|  | Cohort 1 or Cohort 2 Membership | 0.593 | -0.522 | 0.074 | -7.030 | 0.000 | (0.513, 0.686) |
| F84 | Pervasive developmental disorders | 1.413 | 0.346 | 0.080 | 4.335 | 0.000 | (1.209, 1.653) |
| F20-F29 | Schizophrenia, schizotypal, delusional, and other non-mood psychotic disorders | 1.563 | 0.447 | 0.098 | 4.539 | 0.000 | (1.289, 1.896) |
| M | Male | 1.000 | -0.000 | 0.062 | -0.001 | 0.999 | (0.886, 1.128) |
| AI | Age at Index | 1.054 | 0.053 | 0.011 | 4.584 | 0.000 | (1.031, 1.078) |
| 2054-5 | Black or African American | 0.909 | -0.095 | 0.073 | -1.302 | 0.193 | (0.788, 1.049) |
| 2186-5 | Not Hispanic or Latino | 1.091 | 0.087 | 0.061 | 1.439 | 0.150 | (0.969, 1.228) |
| F41.9 | Anxiety disorder, unspecified | 0.897 | -0.109 | 0.061 | -1.799 | 0.072 | (0.796, 1.010) |
| F91 | Conduct disorders | 1.411 | 0.344 | 0.059 | 5.793 | 0.000 | (1.256, 1.585) |
| F41.1 | Generalized anxiety disorder | 0.749 | -0.289 | 0.073 | -3.928 | 0.000 | (0.649, 0.865) |
| F41.0 | Panic disorder [episodic paroxysmal anxiety] | 0.785 | -0.242 | 0.127 | -1.902 | 0.057 | (0.612, 1.007) |
| F42 | Obsessive-compulsive disorder | 1.188 | 0.172 | 0.118 | 1.455 | 0.146 | (0.942, 1.498) |
| F70-F79 | Intellectual Disabilities | 1.523 | 0.421 | 0.127 | 3.311 | 0.001 | (1.187, 1.953) |
| F50 | Eating disorders | 0.952 | -0.049 | 0.123 | -0.399 | 0.690 | (0.748, 1.211) |
| F51 | Sleep disorders not due to a substance or known physiological condition | 0.921 | -0.082 | 0.108 | -0.761 | 0.447 | (0.746, 1.138) |
| F10-F19 | Mental and behavioral disorders due to psychoactive substance use | 1.037 | 0.036 | 0.090 | 0.406 | 0.685 | (0.870, 1.237) |
| F60.3 | Borderline personality disorder | 1.525 | 0.422 | 0.124 | 3.408 | 0.001 | (1.197, 1.945) |
| F30-F39 | Mood [affective] disorders | 2.520 | 0.924 | 0.078 | 11.789 | 0.000 | (2.161, 2.939) |

**SA7.5. Cohorts: Non-Stimulants (vs. Antidepressants)**

### Query Criteria for Cohort 1 (query name: PTSD_onNS_Not on AD)

This query was run on the network Research with 102 HCO(s) queried and 102 HCO(s) responded. A total of 62 provider(s) responded with patients. The final cohort included 5,075 patients who matched the query criteria listed in the table below.

| Cohort 1 | | | | | |
| --- | --- | --- | --- | --- | --- |
|  | must have |  | demographics | Age | Age (between 6 and 18 years (most recent occurrence)) |
|  |  | and | diagnosis | UMLS:ICD10CM:F90 | Attention-deficit hyperactivity disorders |
|  |  | and | diagnosis | UMLS:ICD10CM:F43.1 | Post-traumatic stress disorder (PTSD) |
|  |  | and any of | medication | NLM:RXNORM:38400 | atomoxetine |
|  |  |  | medication | NLM:RXNORM:11196 | viloxazine |
|  |  |  | medication | NLM:RXNORM:2599 | clonidine |
|  |  |  | medication | NLM:RXNORM:40114 | guanfacine |
|  | cannot have |  | medication | NLM:VA:CN600 | ANTIDEPRESSANTS |

### Query Criteria for Cohort 2 (query name: PTSD_onAD_Not on NS)

This query was run on the network Research with 102 HCO(s) queried and 102 HCO(s) responded. A total of 65 provider(s) responded with patients. The final cohort included 6,269 patients who matched the query criteria listed in the table below.

| Cohort 2 | | | | | |
| --- | --- | --- | --- | --- | --- |
|  | must have |  | demographics | Age | Age (between 6 and 18 years (most recent occurrence)) |
|  |  | and | diagnosis | UMLS:ICD10CM:F90 | Attention-deficit hyperactivity disorders |
|  |  | and | diagnosis | UMLS:ICD10CM:F43.1 | Post-traumatic stress disorder (PTSD) |
|  |  | and | medication | NLM:VA:CN600 | ANTIDEPRESSANTS |
|  | cannot have |  | medication | NLM:RXNORM:38400 | atomoxetine |
|  |  | or | medication | NLM:RXNORM:11196 | viloxazine |
|  |  | or | medication | NLM:RXNORM:2599 | clonidine |
|  |  | or | medication | NLM:RXNORM:40114 | guanfacine |

| **Cohort Information** | | |
| --- | --- | --- |
|  | **Cohort Name** | **Index Count** |
|  | PTSD_onNS_Not on AD | 5091 |
|  | PTSD_onAD_Not on NS | 6330 |

**SA7.51: Hospital Inpatient and Observation Care Services**

| **Cox Model Results** | | | | | | | |
| --- | --- | --- | --- | --- | --- | --- | --- |
|  | **Covariate** | **Hazard Ratio** | **Coefficient** | **Standard Error** | **z** | **P > \|z\|** | **95% Confidence Interval** |
|  | Cohort 1 or Cohort 2 Membership | 0.763 | -0.270 | 0.054 | -5.021 | 0.000 | (0.687, 0.848) |
| F84 | Pervasive developmental disorders | 1.162 | 0.150 | 0.062 | 2.398 | 0.016 | (1.028, 1.313) |
| F20-F29 | Schizophrenia, schizotypal, delusional, and other non-mood psychotic disorders | 1.549 | 0.438 | 0.077 | 5.714 | 0.000 | (1.333, 1.800) |
| M | Male | 0.886 | -0.121 | 0.044 | -2.736 | 0.006 | (0.813, 0.966) |
| AI | Age at Index | 1.018 | 0.018 | 0.008 | 2.206 | 0.027 | (1.002, 1.034) |
| 2054-5 | Black or African American | 1.140 | 0.131 | 0.049 | 2.643 | 0.008 | (1.034, 1.256) |
| 2186-5 | Not Hispanic or Latino | 1.367 | 0.312 | 0.044 | 7.145 | 0.000 | (1.254, 1.489) |
| F41.9 | Anxiety disorder, unspecified | 0.898 | -0.108 | 0.042 | -2.585 | 0.010 | (0.828, 0.974) |
| F91 | Conduct disorders | 1.159 | 0.147 | 0.044 | 3.380 | 0.001 | (1.064, 1.262) |
| F41.1 | Generalized anxiety disorder | 1.074 | 0.072 | 0.047 | 1.533 | 0.125 | (0.980, 1.178) |
| F41.0 | Panic disorder [episodic paroxysmal anxiety] | 1.395 | 0.333 | 0.068 | 4.888 | 0.000 | (1.220, 1.594) |
| F42 | Obsessive-compulsive disorder | 1.137 | 0.129 | 0.083 | 1.550 | 0.121 | (0.967, 1.338) |
| F70-F79 | Intellectual Disabilities | 1.144 | 0.135 | 0.114 | 1.182 | 0.237 | (0.915, 1.431) |
| F50 | Eating disorders | 1.198 | 0.181 | 0.073 | 2.469 | 0.014 | (1.038, 1.383) |
| F51 | Sleep disorders not due to a substance or known physiological condition | 0.926 | -0.077 | 0.077 | -0.998 | 0.318 | (0.797, 1.077) |
| F10-F19 | Mental and behavioral disorders due to psychoactive substance use | 1.768 | 0.570 | 0.054 | 10.489 | 0.000 | (1.589, 1.966) |
| F60.3 | Borderline personality disorder | 1.730 | 0.548 | 0.089 | 6.193 | 0.000 | (1.455, 2.058) |
| F30-F39 | Mood [affective] disorders | 2.326 | 0.844 | 0.055 | 15.438 | 0 | (2.089, 2.589) |

**SA7.52: Emergency Department Services**

| **Cox Model Results** | | | | | | | |
| --- | --- | --- | --- | --- | --- | --- | --- |
|  | **Covariate** | **Hazard Ratio** | **Coefficient** | **Standard Error** | **z** | **P > \|z\|** | **95% Confidence Interval** |
|  | Cohort 1 or Cohort 2 Membership | 0.950 | -0.051 | 0.045 | -1.130 | 0.259 | (0.870, 1.038) |
| F84 | Pervasive developmental disorders | 0.975 | -0.025 | 0.059 | -0.428 | 0.669 | (0.869, 1.094) |
| F20-F29 | Schizophrenia, schizotypal, delusional, and other non-mood psychotic disorders | 1.094 | 0.090 | 0.086 | 1.040 | 0.298 | (0.924, 1.296) |
| M | Male | 0.960 | -0.041 | 0.038 | -1.075 | 0.282 | (0.891, 1.034) |
| AI | Age at Index | 1.048 | 0.047 | 0.007 | 6.714 | 0.000 | (1.034, 1.063) |
| 2054-5 | Black or African American | 1.185 | 0.170 | 0.042 | 4.044 | 0.000 | (1.091, 1.287) |
| 2186-5 | Not Hispanic or Latino | 1.013 | 0.013 | 0.037 | 0.347 | 0.728 | (0.942, 1.090) |
| F41.9 | Anxiety disorder, unspecified | 1.058 | 0.057 | 0.037 | 1.523 | 0.128 | (0.984, 1.139) |
| F91 | Conduct disorders | 1.206 | 0.187 | 0.037 | 5.011 | 0.000 | (1.121, 1.298) |
| F41.1 | Generalized anxiety disorder | 0.859 | -0.152 | 0.045 | -3.343 | 0.001 | (0.786, 0.939) |
| F41.0 | Panic disorder [episodic paroxysmal anxiety] | 1.192 | 0.176 | 0.075 | 2.353 | 0.019 | (1.030, 1.380) |
| F42 | Obsessive-compulsive disorder | 0.980 | -0.020 | 0.087 | -0.235 | 0.814 | (0.826, 1.162) |
| F70-F79 | Intellectual Disabilities | 1.010 | 0.010 | 0.103 | 0.093 | 0.926 | (0.826, 1.234) |
| F50 | Eating disorders | 0.879 | -0.129 | 0.081 | -1.596 | 0.110 | (0.750, 1.030) |
| F51 | Sleep disorders not due to a substance or known physiological condition | 1.034 | 0.033 | 0.065 | 0.518 | 0.605 | (0.911, 1.173) |
| F10-F19 | Mental and behavioral disorders due to psychoactive substance use | 1.227 | 0.205 | 0.059 | 3.476 | 0.001 | (1.093, 1.377) |
| F60.3 | Borderline personality disorder | 1.300 | 0.262 | 0.098 | 2.682 | 0.007 | (1.073, 1.574) |
| F30-F39 | Mood [affective] disorders | 1.405 | 0.340 | 0.042 | 8.166 | 0.000 | (1.295, 1.525) |

**SA7.53: Antipsychotics**

| **Cox Model Results** | | | | | | | |
| --- | --- | --- | --- | --- | --- | --- | --- |
|  | **Covariate** | **Hazard Ratio** | **Coefficient** | **Standard Error** | **z** | **P > \|z\|** | **95% Confidence Interval** |
|  | Cohort 1 or Cohort 2 Membership | 1.138 | 0.129 | 0.045 | 2.852 | 0.004 | (1.041, 1.243) |
| F84 | Pervasive developmental disorders | 1.416 | 0.348 | 0.051 | 6.790 | 0.000 | (1.281, 1.566) |
| F20-F29 | Schizophrenia, schizotypal, delusional, and other non-mood psychotic disorders | 2.225 | 0.800 | 0.066 | 12.160 | 0.000 | (1.956, 2.531) |
| M | Male | 1.026 | 0.026 | 0.038 | 0.672 | 0.501 | (0.952, 1.106) |
| AI | Age at Index | 0.999 | -0.001 | 0.007 | -0.101 | 0.920 | (0.986, 1.013) |
| 2054-5 | Black or African American | 1.048 | 0.047 | 0.044 | 1.069 | 0.285 | (0.962, 1.142) |
| 2186-5 | Not Hispanic or Latino | 0.845 | -0.168 | 0.036 | -4.601 | 0.000 | (0.787, 0.908) |
| F41.9 | Anxiety disorder, unspecified | 0.829 | -0.188 | 0.038 | -4.977 | 0.000 | (0.769, 0.892) |
| F91 | Conduct disorders | 1.392 | 0.331 | 0.037 | 8.877 | 0.000 | (1.294, 1.497) |
| F41.1 | Generalized anxiety disorder | 0.871 | -0.138 | 0.045 | -3.043 | 0.002 | (0.797, 0.952) |
| F41.0 | Panic disorder [episodic paroxysmal anxiety] | 1.187 | 0.171 | 0.072 | 2.379 | 0.017 | (1.031, 1.367) |
| F42 | Obsessive-compulsive disorder | 1.187 | 0.172 | 0.078 | 2.193 | 0.028 | (1.018, 1.384) |
| F70-F79 | Intellectual Disabilities | 1.416 | 0.348 | 0.089 | 3.895 | 0.000 | (1.189, 1.686) |
| F50 | Eating disorders | 1.077 | 0.074 | 0.074 | 1.009 | 0.313 | (0.932, 1.244) |
| F51 | Sleep disorders not due to a substance or known physiological condition | 0.905 | -0.100 | 0.068 | -1.464 | 0.143 | (0.792, 1.034) |
| F10-F19 | Mental and behavioral disorders due to psychoactive substance use | 1.474 | 0.388 | 0.054 | 7.181 | 0.000 | (1.326, 1.639) |
| F60.3 | Borderline personality disorder | 1.599 | 0.470 | 0.085 | 5.533 | 0.000 | (1.354, 1.889) |
| F30-F39 | Mood [affective] disorders | 2.606 | 0.958 | 0.045 | 21.109 | 0 | (2.384, 2.848) |

**SA7.54: Mood Stabilizers**

| **Cox Model Results** | | | | | | | |
| --- | --- | --- | --- | --- | --- | --- | --- |
|  | **Covariate** | **Hazard Ratio** | **Coefficient** | **Standard Error** | **z** | **P > \|z\|** | **95% Confidence Interval** |
|  | Cohort 1 or Cohort 2 Membership | 1.446 | 0.369 | 0.079 | 4.661 | 0.000 | (1.238, 1.688) |
| F84 | Pervasive developmental disorders | 1.177 | 0.163 | 0.092 | 1.771 | 0.077 | (0.983, 1.410) |
| F20-F29 | Schizophrenia, schizotypal, delusional, and other non-mood psychotic disorders | 2.111 | 0.747 | 0.106 | 7.052 | 0.000 | (1.715, 2.598) |
| M | Male | 0.872 | -0.137 | 0.068 | -2.020 | 0.043 | (0.764, 0.996) |
| AI | Age at Index | 1.080 | 0.077 | 0.012 | 6.189 | 0.000 | (1.054, 1.106) |
| 2054-5 | Black or African American | 0.814 | -0.206 | 0.080 | -2.566 | 0.010 | (0.696, 0.953) |
| 2186-5 | Not Hispanic or Latino | 1.020 | 0.019 | 0.064 | 0.302 | 0.763 | (0.899, 1.156) |
| F41.9 | Anxiety disorder, unspecified | 0.883 | -0.125 | 0.065 | -1.935 | 0.053 | (0.778, 1.002) |
| F91 | Conduct disorders | 1.204 | 0.186 | 0.066 | 2.796 | 0.005 | (1.057, 1.372) |
| F41.1 | Generalized anxiety disorder | 0.822 | -0.196 | 0.077 | -2.532 | 0.011 | (0.706, 0.957) |
| F41.0 | Panic disorder [episodic paroxysmal anxiety] | 1.086 | 0.083 | 0.121 | 0.683 | 0.495 | (0.857, 1.377) |
| F42 | Obsessive-compulsive disorder | 1.414 | 0.347 | 0.121 | 2.862 | 0.004 | (1.115, 1.793) |
| F70-F79 | Intellectual Disabilities | 1.701 | 0.531 | 0.141 | 3.775 | 0.000 | (1.291, 2.241) |
| F50 | Eating disorders | 1.216 | 0.196 | 0.118 | 1.662 | 0.096 | (0.966, 1.531) |
| F51 | Sleep disorders not due to a substance or known physiological condition | 1.093 | 0.089 | 0.111 | 0.802 | 0.423 | (0.879, 1.359) |
| F10-F19 | Mental and behavioral disorders due to psychoactive substance use | 1.172 | 0.159 | 0.094 | 1.684 | 0.092 | (0.974, 1.410) |
| F60.3 | Borderline personality disorder | 1.293 | 0.257 | 0.148 | 1.733 | 0.083 | (0.967, 1.729) |
| F30-F39 | Mood [affective] disorders | 2.696 | 0.992 | 0.082 | 12.026 | 0.000 | (2.293, 3.168) |

**SA7.6. Cohorts: CNS Stimulants (vs. Non-Stimulants)**

### Query Criteria for Cohort 1 (query name: ADHD+PTSD+Exclusive ST)

This query was run on the network Research with 100 HCO(s) queried and 100 HCO(s) responded. A total of 61 provider(s) responded with patients. The final cohort included 5,400 patients who matched the query criteria listed in the table below.

| Cohort 1 | | | | | |
| --- | --- | --- | --- | --- | --- |
|  | must have |  | demographics | Age | Age (between 6 and 18 years (most recent occurrence)) |
|  |  | and | diagnosis | UMLS:ICD10CM:F90 | Attention-deficit hyperactivity disorders |
|  |  | and | diagnosis | UMLS:ICD10CM:F43.1 | Post-traumatic stress disorder (PTSD) |
|  |  | and any of | medication | NLM:VA:CN801 | AMPHETAMINES |
|  |  |  | medication | NLM:VA:CN802 | AMPHETAMINE LIKE STIMULANTS |
|  | cannot have |  | medication | NLM:RXNORM:40114 | guanfacine |
|  |  | or | medication | NLM:RXNORM:38400 | atomoxetine |
|  |  | or | medication | NLM:RXNORM:2599 | clonidine |
|  |  | or | medication | NLM:RXNORM:11196 | viloxazine |

### Query Criteria for Cohort 2 (query name: ADHD+PTSD+Exclusive NS)

This query was run on the network Research with 100 HCO(s) queried and 100 HCO(s) responded. A total of 61 provider(s) responded with patients. The final cohort included 4,727 patients who matched the query criteria listed in the table below.

| Cohort 2 | | | | | |
| --- | --- | --- | --- | --- | --- |
|  | must have |  | demographics | Age | Age (between 6 and 18 years (most recent occurrence)) |
|  |  | and | diagnosis | UMLS:ICD10CM:F90 | Attention-deficit hyperactivity disorders |
|  |  | and | diagnosis | UMLS:ICD10CM:F43.1 | Post-traumatic stress disorder (PTSD) |
|  |  | and any of | medication | NLM:RXNORM:38400 | atomoxetine |
|  |  |  | medication | NLM:RXNORM:40114 | guanfacine |
|  |  |  | medication | NLM:RXNORM:2599 | clonidine |
|  |  |  | medication | NLM:RXNORM:11196 | viloxazine |
|  | cannot have |  | medication | NLM:VA:CN801 | AMPHETAMINES |
|  |  | or | medication | NLM:VA:CN802 | AMPHETAMINE LIKE STIMULANTS |

| **Cohort Information** | | |
| --- | --- | --- |
|  | **Cohort Name** | **Index Count** |
|  | ADHD+PTSD+Exclusive ST | 6166 |
|  | ADHD+PTSD+Exclusive NS | 5325 |

**SA7.61: Hospital Inpatient and Observation Care Services**

| **Cox Model Results** | | | | | | | |
| --- | --- | --- | --- | --- | --- | --- | --- |
|  | **Covariate** | **Hazard Ratio** | **Coefficient** | **Standard Error** | **z** | **P > \|z\|** | **95% Confidence Interval** |
|  | Cohort 1 or Cohort 2 Membership | 0.685 | -0.378 | 0.041 | -9.293 | 0.000 | (0.633, 0.742) |
| F84 | Pervasive developmental disorders | 1.188 | 0.172 | 0.060 | 2.879 | 0.004 | (1.057, 1.336) |
| F20-F29 | Schizophrenia, schizotypal, delusional, and other non-mood psychotic disorders | 1.448 | 0.370 | 0.073 | 5.038 | 0.000 | (1.254, 1.672) |
| M | Male | 0.869 | -0.140 | 0.043 | -3.287 | 0.001 | (0.799, 0.945) |
| AI | Age at Index | 1.033 | 0.032 | 0.008 | 4.120 | 0.000 | (1.017, 1.049) |
| 2054-5 | Black or African American | 1.047 | 0.046 | 0.052 | 0.887 | 0.375 | (0.946, 1.160) |
| 2186-5 | Not Hispanic or Latino | 1.457 | 0.376 | 0.044 | 8.498 | 0.000 | (1.336, 1.589) |
| F41.9 | Anxiety disorder, unspecified | 0.988 | -0.012 | 0.042 | -0.290 | 0.772 | (0.910, 1.073) |
| F91 | Conduct disorders | 1.239 | 0.214 | 0.042 | 5.111 | 0.000 | (1.141, 1.345) |
| F41.1 | Generalized anxiety disorder | 1.056 | 0.055 | 0.047 | 1.173 | 0.241 | (0.964, 1.158) |
| F41.0 | Panic disorder [episodic paroxysmal anxiety] | 1.314 | 0.273 | 0.070 | 3.925 | 0.000 | (1.147, 1.506) |
| F42 | Obsessive-compulsive disorder | 1.366 | 0.312 | 0.075 | 4.160 | 0.000 | (1.179, 1.582) |
| F70-F79 | Intellectual Disabilities | 1.228 | 0.206 | 0.103 | 1.994 | 0.046 | (1.004, 1.503) |
| F50 | Eating disorders | 1.191 | 0.175 | 0.074 | 2.358 | 0.018 | (1.030, 1.377) |
| F51 | Sleep disorders not due to a substance or known physiological condition | 0.853 | -0.159 | 0.077 | -2.064 | 0.039 | (0.733, 0.992) |
| F10-F19 | Mental and behavioral disorders due to psychoactive substance use | 1.677 | 0.517 | 0.055 | 9.319 | 0.000 | (1.504, 1.869) |
| F60.3 | Borderline personality disorder | 1.634 | 0.491 | 0.086 | 5.717 | 0.000 | (1.381, 1.934) |
| F30-F39 | Mood [affective] disorders | 2.506 | 0.919 | 0.055 | 16.790 | 0 | (2.251, 2.790) |

**SA7.62: Emergency Department Services**

| **Cox Model Results** | | | | | | | |
| --- | --- | --- | --- | --- | --- | --- | --- |
|  | **Covariate** | **Hazard Ratio** | **Coefficient** | **Standard Error** | **z** | **P > \|z\|** | **95% Confidence Interval** |
|  | Cohort 1 or Cohort 2 Membership | 0.726 | -0.320 | 0.036 | -8.931 | 0.000 | (0.677, 0.779) |
| F84 | Pervasive developmental disorders | 1.006 | 0.006 | 0.057 | 0.100 | 0.920 | (0.899, 1.125) |
| F20-F29 | Schizophrenia, schizotypal, delusional, and other non-mood psychotic disorders | 1.407 | 0.342 | 0.073 | 4.676 | 0.000 | (1.219, 1.624) |
| M | Male | 0.911 | -0.093 | 0.037 | -2.524 | 0.012 | (0.847, 0.979) |
| AI | Age at Index | 1.073 | 0.070 | 0.007 | 10.156 | 0.000 | (1.058, 1.087) |
| 2054-5 | Black or African American | 1.084 | 0.081 | 0.044 | 1.818 | 0.069 | (0.994, 1.183) |
| 2186-5 | Not Hispanic or Latino | 1.016 | 0.016 | 0.037 | 0.421 | 0.674 | (0.944, 1.093) |
| F41.9 | Anxiety disorder, unspecified | 1.119 | 0.112 | 0.037 | 3.018 | 0.003 | (1.040, 1.204) |
| F91 | Conduct disorders | 1.176 | 0.162 | 0.037 | 4.347 | 0.000 | (1.093, 1.265) |
| F41.1 | Generalized anxiety disorder | 0.806 | -0.215 | 0.045 | -4.776 | 0.000 | (0.738, 0.881) |
| F41.0 | Panic disorder [episodic paroxysmal anxiety] | 1.272 | 0.240 | 0.071 | 3.371 | 0.001 | (1.106, 1.462) |
| F42 | Obsessive-compulsive disorder | 0.948 | -0.053 | 0.083 | -0.642 | 0.521 | (0.806, 1.115) |
| F70-F79 | Intellectual Disabilities | 0.982 | -0.018 | 0.103 | -0.174 | 0.862 | (0.803, 1.202) |
| F50 | Eating disorders | 0.965 | -0.036 | 0.080 | -0.449 | 0.653 | (0.824, 1.129) |
| F51 | Sleep disorders not due to a substance or known physiological condition | 0.999 | -0.001 | 0.066 | -0.010 | 0.992 | (0.878, 1.137) |
| F10-F19 | Mental and behavioral disorders due to psychoactive substance use | 1.143 | 0.134 | 0.060 | 2.242 | 0.025 | (1.017, 1.285) |
| F60.3 | Borderline personality disorder | 1.064 | 0.062 | 0.098 | 0.637 | 0.524 | (0.878, 1.290) |
| F30-F39 | Mood [affective] disorders | 1.377 | 0.320 | 0.042 | 7.615 | 0.000 | (1.268, 1.495) |

**SA7.63: Antipsychotics**

| **Cox Model Results** | | | | | | | |
| --- | --- | --- | --- | --- | --- | --- | --- |
|  | **Covariate** | **Hazard Ratio** | **Coefficient** | **Standard Error** | **z** | **P > \|z\|** | **95% Confidence Interval** |
|  | Cohort 1 or Cohort 2 Membership | 0.515 | -0.664 | 0.036 | -18.335 | 0 | (0.480, 0.553) |
| F84 | Pervasive developmental disorders | 1.460 | 0.378 | 0.049 | 7.742 | 0.000 | (1.327, 1.607) |
| F20-F29 | Schizophrenia, schizotypal, delusional, and other non-mood psychotic disorders | 1.941 | 0.663 | 0.062 | 10.744 | 0.000 | (1.720, 2.191) |
| M | Male | 0.920 | -0.084 | 0.036 | -2.301 | 0.021 | (0.856, 0.988) |
| AI | Age at Index | 1.033 | 0.032 | 0.007 | 4.911 | 0.000 | (1.020, 1.046) |
| 2054-5 | Black or African American | 1.016 | 0.016 | 0.046 | 0.358 | 0.720 | (0.929, 1.112) |
| 2186-5 | Not Hispanic or Latino | 0.888 | -0.119 | 0.036 | -3.325 | 0.001 | (0.827, 0.952) |
| F41.9 | Anxiety disorder, unspecified | 0.839 | -0.176 | 0.037 | -4.795 | 0.000 | (0.781, 0.901) |
| F91 | Conduct disorders | 1.360 | 0.307 | 0.036 | 8.544 | 0.000 | (1.267, 1.459) |
| F41.1 | Generalized anxiety disorder | 0.869 | -0.140 | 0.043 | -3.289 | 0.001 | (0.799, 0.945) |
| F41.0 | Panic disorder [episodic paroxysmal anxiety] | 0.900 | -0.106 | 0.072 | -1.469 | 0.142 | (0.782, 1.036) |
| F42 | Obsessive-compulsive disorder | 1.218 | 0.197 | 0.071 | 2.785 | 0.005 | (1.060, 1.399) |
| F70-F79 | Intellectual Disabilities | 1.335 | 0.289 | 0.084 | 3.454 | 0.001 | (1.133, 1.572) |
| F50 | Eating disorders | 0.915 | -0.089 | 0.075 | -1.186 | 0.236 | (0.789, 1.060) |
| F51 | Sleep disorders not due to a substance or known physiological condition | 1.056 | 0.055 | 0.063 | 0.867 | 0.386 | (0.934, 1.195) |
| F10-F19 | Mental and behavioral disorders due to psychoactive substance use | 1.261 | 0.232 | 0.054 | 4.333 | 0.000 | (1.135, 1.400) |
| F60.3 | Borderline personality disorder | 1.444 | 0.367 | 0.081 | 4.534 | 0.000 | (1.232, 1.692) |
| F30-F39 | Mood [affective] disorders | 2.692 | 0.990 | 0.046 | 21.706 | 0 | (2.462, 2.944) |

**SA7.64: Mood Stabilizers**

| **Cox Model Results** | | | | | | | |
| --- | --- | --- | --- | --- | --- | --- | --- |
|  | **Covariate** | **Hazard Ratio** | **Coefficient** | **Standard Error** | **z** | **P > \|z\|** | **95% Confidence Interval** |
|  | Cohort 1 or Cohort 2 Membership | 0.520 | -0.653 | 0.060 | -10.918 | 0.000 | (0.463, 0.585) |
| F84 | Pervasive developmental disorders | 1.342 | 0.294 | 0.081 | 3.639 | 0.000 | (1.145, 1.572) |
| F20-F29 | Schizophrenia, schizotypal, delusional, and other non-mood psychotic disorders | 1.512 | 0.414 | 0.100 | 4.152 | 0.000 | (1.244, 1.838) |
| M | Male | 0.867 | -0.143 | 0.061 | -2.358 | 0.018 | (0.770, 0.976) |
| AI | Age at Index | 1.100 | 0.095 | 0.011 | 8.308 | 0.000 | (1.075, 1.125) |
| 2054-5 | Black or African American | 0.951 | -0.050 | 0.076 | -0.655 | 0.512 | (0.819, 1.105) |
| 2186-5 | Not Hispanic or Latino | 0.985 | -0.015 | 0.059 | -0.249 | 0.803 | (0.877, 1.107) |
| F41.9 | Anxiety disorder, unspecified | 0.861 | -0.150 | 0.060 | -2.514 | 0.012 | (0.766, 0.967) |
| F91 | Conduct disorders | 1.322 | 0.279 | 0.059 | 4.716 | 0.000 | (1.177, 1.485) |
| F41.1 | Generalized anxiety disorder | 0.810 | -0.211 | 0.069 | -3.036 | 0.002 | (0.707, 0.928) |
| F41.0 | Panic disorder [episodic paroxysmal anxiety] | 1.087 | 0.083 | 0.107 | 0.779 | 0.436 | (0.882, 1.339) |
| F42 | Obsessive-compulsive disorder | 1.274 | 0.242 | 0.108 | 2.234 | 0.025 | (1.030, 1.575) |
| F70-F79 | Intellectual Disabilities | 1.509 | 0.411 | 0.128 | 3.207 | 0.001 | (1.173, 1.940) |
| F50 | Eating disorders | 0.962 | -0.039 | 0.117 | -0.330 | 0.741 | (0.765, 1.210) |
| F51 | Sleep disorders not due to a substance or known physiological condition | 0.982 | -0.018 | 0.105 | -0.169 | 0.866 | (0.799, 1.207) |
| F10-F19 | Mental and behavioral disorders due to psychoactive substance use | 1.063 | 0.061 | 0.087 | 0.704 | 0.481 | (0.897, 1.260) |
| F60.3 | Borderline personality disorder | 1.758 | 0.564 | 0.117 | 4.820 | 0.000 | (1.398, 2.211) |
| F30-F39 | Mood [affective] disorders | 2.511 | 0.921 | 0.078 | 11.850 | 0.000 | (2.156, 2.924) |

**Supplement Analysis 8. Sensitivity Analysis – Association of Sustained ADHD Medication Exposure on Acute Clinical Outcomes in Individuals with ADHD PTSD: Cox Proportional Hazards Models Adjusted for Demographics and Comorbidities**

**Cohorts: CNS stimulants (vs Non-stimulants)**

Query Criteria for Cohort 1 (query name: PTSD_2 Script ST (No nonStimulants))

This query was run on the network Research with 111 HCO(s) queried and 111 HCO(s) responded. A total of 70 provider(s) responded with patients. The final cohort included 5,442 patients who matched the query criteria listed in the table below.

| Cohort 1 | | | | | |
| --- | --- | --- | --- | --- | --- |
|  | must have |  | demographics | Age | Age (between 3 and 18 years (most recent occurrence)) |
|  |  | and | diagnosis | UMLS:ICD10CM:F90 | Attention-deficit hyperactivity disorders |
|  |  | and | diagnosis | UMLS:ICD10CM:F43.1 | Post-traumatic stress disorder (PTSD) |
|  | cannot have |  | medication | NLM:RXNORM:2599 | clonidine |
|  |  | or | medication | NLM:RXNORM:38400 | atomoxetine |
|  |  | or | medication | NLM:RXNORM:11196 | viloxazine |
|  |  | or | medication | NLM:RXNORM:40114 | guanfacine |
| Group 1 | | | | | |
|  | **Group 1A** | | | | |
|  | must have | any of | medication | NLM:VA:CN801 | AMPHETAMINES |
|  |  |  | medication | NLM:VA:CN802 | AMPHETAMINE LIKE STIMULANTS |
|  | date constraint | | The terms in this group occurred at any time | | |
|  | event relationship | | Any instance of Group 1B occurred within 6 months on or before any instance of Group 1A | | |
|  | **Group 1B** | | | | |
|  | must have | any of | medication | NLM:VA:CN801 | AMPHETAMINES |
|  |  |  | medication | NLM:VA:CN802 | AMPHETAMINE LIKE STIMULANTS |
| Group 2 | | | | | |
|  | **Group 2A** | | | | |
|  | must have | any of | medication | NLM:VA:CN801 | AMPHETAMINES |
|  |  |  | medication | NLM:VA:CN802 | AMPHETAMINE LIKE STIMULANTS |
|  | number of instances | | Greater than or equal to 2 instances | | |
|  | date constraint | | The terms in this group occurred at any time | | |

### Query Criteria for Cohort 2 (query name: PTSD_2 Script NS (No Stimulants))

This query was run on the network Research with 111 HCO(s) queried and 111 HCO(s) responded. A total of 67 provider(s) responded with patients. The final cohort included 4,741 patients who matched the query criteria listed in the table below.

| Cohort 2 | | | | | |
| --- | --- | --- | --- | --- | --- |
|  | must have |  | demographics | Age | Age (between 3 and 18 years (most recent occurrence)) |
|  |  | and | diagnosis | UMLS:ICD10CM:F90 | Attention-deficit hyperactivity disorders |
|  |  | and | diagnosis | UMLS:ICD10CM:F43.1 | Post-traumatic stress disorder (PTSD) |
|  | cannot have |  | medication | NLM:VA:CN801 | AMPHETAMINES |
|  |  | or | medication | NLM:VA:CN802 | AMPHETAMINE LIKE STIMULANTS |
| Group 1 | | | | | |
|  | **Group 1A** | | | | |
|  | must have | any of | medication | NLM:RXNORM:40114 | guanfacine |
|  |  |  | medication | NLM:RXNORM:11196 | viloxazine |
|  |  |  | medication | NLM:RXNORM:38400 | atomoxetine |
|  |  |  | medication | NLM:RXNORM:2599 | clonidine |
|  | date constraint | | The terms in this group occurred at any time | | |
|  | event relationship | | Any instance of Group 1B occurred within 6 months on or before any instance of Group 1A | | |
|  | **Group 1B** | | | | |
|  | must have | any of | medication | NLM:RXNORM:2599 | clonidine |
|  |  |  | medication | NLM:RXNORM:40114 | guanfacine |
|  |  |  | medication | NLM:RXNORM:38400 | atomoxetine |
|  |  |  | medication | NLM:RXNORM:11196 | viloxazine |
| Group 2 | | | | | |
|  | **Group 2A** | | | | |
|  | must have | any of | medication | NLM:RXNORM:2599 | clonidine |
|  |  |  | medication | NLM:RXNORM:40114 | guanfacine |
|  |  |  | medication | NLM:RXNORM:38400 | atomoxetine |
|  |  |  | medication | NLM:RXNORM:11196 | viloxazine |
|  | number of instances | | Greater than or equal to 2 instances | | |
|  | date constraint | | The terms in this group occurred at any time | | |

| **Cohort Information** | | |
| --- | --- | --- |
|  | **Cohort Name** | **Index Count** |
|  | PTSD_2 Script ST (No nonStimulants) | 5436 |
|  | PTSD_2 Script NS (No Stimulants) | 4741 |

**SA8.1: Hospital Inpatient and Observation Care Services**

| **Cox Model Results** | | | | | | | |
| --- | --- | --- | --- | --- | --- | --- | --- |
|  | **Covariate** | **Hazard Ratio** | **Coefficient** | **Standard Error** | **z** | **P > \|z\|** | **95% Confidence Interval** |
|  | Cohort 1 or Cohort 2 Membership | 0.599 | -0.513 | 0.045 | -11.300 | 0.000 | (0.548, 0.655) |
| M | Male | 0.935 | -0.068 | 0.046 | -1.458 | 0.145 | (0.854, 1.024) |
| AI | Age at Index | 1.025 | 0.025 | 0.008 | 3.038 | 0.002 | (1.009, 1.042) |
| 2054-5 | Black or African American | 1.067 | 0.065 | 0.056 | 1.158 | 0.247 | (0.956, 1.192) |
| 2186-5 | Not Hispanic or Latino | 1.461 | 0.379 | 0.050 | 7.583 | 0.000 | (1.325, 1.612) |
| F41.9 | Anxiety disorder, unspecified | 1.031 | 0.030 | 0.046 | 0.659 | 0.510 | (0.942, 1.128) |
| F91 | Conduct disorders | 1.352 | 0.302 | 0.046 | 6.561 | 0.000 | (1.236, 1.479) |
| F41.1 | Generalized anxiety disorder | 0.958 | -0.043 | 0.052 | -0.830 | 0.407 | (0.866, 1.060) |
| F41.0 | Panic disorder [episodic paroxysmal anxiety] | 1.439 | 0.364 | 0.074 | 4.920 | 0.000 | (1.245, 1.664) |
| F42 | Obsessive-compulsive disorder | 1.424 | 0.354 | 0.080 | 4.434 | 0.000 | (1.218, 1.665) |
| F70-F79 | Intellectual Disabilities | 1.490 | 0.399 | 0.105 | 3.785 | 0.000 | (1.212, 1.831) |
| F50 | Eating disorders | 1.111 | 0.105 | 0.082 | 1.278 | 0.201 | (0.946, 1.305) |
| F51 | Sleep disorders not due to a substance or known physiological condition | 0.883 | -0.125 | 0.085 | -1.475 | 0.140 | (0.748, 1.042) |
| F10-F19 | Mental and behavioral disorders due to psychoactive substance use | 1.545 | 0.435 | 0.060 | 7.297 | 0.000 | (1.375, 1.737) |
| F30-F39 | Mood [affective] disorders | 2.993 | 1.096 | 0.062 | 17.770 | 0 | (2.652, 3.377) |
| F60.3 | Borderline personality disorder | 1.674 | 0.515 | 0.091 | 5.644 | 0.000 | (1.400, 2.002) |

**SA8.2: Emergency Department Services**

| **Cox Model Results** | | | | | | | |
| --- | --- | --- | --- | --- | --- | --- | --- |
|  | **Covariate** | **Hazard Ratio** | **Coefficient** | **Standard Error** | **z** | **P > \|z\|** | **95% Confidence Interval** |
|  | Cohort 1 or Cohort 2 Membership | 0.634 | -0.456 | 0.051 | -8.864 | 0.000 | (0.573, 0.701) |
| M | Male | 0.949 | -0.053 | 0.053 | -0.995 | 0.320 | (0.855, 1.052) |
| AI | Age at Index | 1.038 | 0.037 | 0.009 | 3.978 | 0.000 | (1.019, 1.057) |
| 2054-5 | Black or African American | 1.126 | 0.119 | 0.064 | 1.864 | 0.062 | (0.994, 1.276) |
| 2186-5 | Not Hispanic or Latino | 0.972 | -0.029 | 0.054 | -0.533 | 0.594 | (0.875, 1.079) |
| F41.9 | Anxiety disorder, unspecified | 1.214 | 0.194 | 0.053 | 3.646 | 0.000 | (1.094, 1.348) |
| F91 | Conduct disorders | 1.283 | 0.250 | 0.053 | 4.673 | 0.000 | (1.156, 1.425) |
| F41.1 | Generalized anxiety disorder | 0.879 | -0.129 | 0.062 | -2.092 | 0.036 | (0.779, 0.992) |
| F41.0 | Panic disorder [episodic paroxysmal anxiety] | 1.291 | 0.256 | 0.093 | 2.762 | 0.006 | (1.077, 1.548) |
| F42 | Obsessive-compulsive disorder | 1.017 | 0.017 | 0.109 | 0.152 | 0.879 | (0.821, 1.259) |
| F70-F79 | Intellectual Disabilities | 1.247 | 0.221 | 0.135 | 1.637 | 0.102 | (0.957, 1.625) |
| F50 | Eating disorders | 1.007 | 0.007 | 0.104 | 0.068 | 0.946 | (0.821, 1.235) |
| F51 | Sleep disorders not due to a substance or known physiological condition | 0.989 | -0.011 | 0.093 | -0.117 | 0.907 | (0.824, 1.187) |
| F10-F19 | Mental and behavioral disorders due to psychoactive substance use | 1.354 | 0.303 | 0.075 | 4.025 | 0.000 | (1.168, 1.569) |
| F30-F39 | Mood [affective] disorders | 1.717 | 0.541 | 0.063 | 8.627 | 0.000 | (1.519, 1.942) |
| F60.3 | Borderline personality disorder | 0.998 | -0.002 | 0.135 | -0.018 | 0.986 | (0.765, 1.301) |

**SA8.3: Antipsychotics**

| **Cox Model Results** | | | | | | | |
| --- | --- | --- | --- | --- | --- | --- | --- |
|  | **Covariate** | **Hazard Ratio** | **Coefficient** | **Standard Error** | **z** | **P > \|z\|** | **95% Confidence Interval** |
|  | Cohort 1 or Cohort 2 Membership | 0.480 | -0.735 | 0.042 | -17.697 | 0 | (0.442, 0.520) |
| M | Male | 1.072 | 0.069 | 0.041 | 1.694 | 0.090 | (0.989, 1.162) |
| AI | Age at Index | 1.046 | 0.045 | 0.007 | 6.250 | 0.000 | (1.032, 1.061) |
| 2054-5 | Black or African American | 1.062 | 0.060 | 0.051 | 1.183 | 0.237 | (0.961, 1.173) |
| 2186-5 | Not Hispanic or Latino | 0.888 | -0.119 | 0.041 | -2.882 | 0.004 | (0.819, 0.963) |
| F41.9 | Anxiety disorder, unspecified | 0.835 | -0.180 | 0.042 | -4.307 | 0.000 | (0.770, 0.907) |
| F91 | Conduct disorders | 1.536 | 0.429 | 0.041 | 10.468 | 0.000 | (1.418, 1.665) |
| F41.1 | Generalized anxiety disorder | 0.857 | -0.154 | 0.048 | -3.190 | 0.001 | (0.780, 0.942) |
| F41.0 | Panic disorder [episodic paroxysmal anxiety] | 0.881 | -0.127 | 0.082 | -1.545 | 0.122 | (0.750, 1.035) |
| F42 | Obsessive-compulsive disorder | 1.249 | 0.222 | 0.081 | 2.753 | 0.006 | (1.066, 1.463) |
| F70-F79 | Intellectual Disabilities | 1.547 | 0.436 | 0.093 | 4.673 | 0.000 | (1.288, 1.858) |
| F50 | Eating disorders | 0.932 | -0.071 | 0.086 | -0.822 | 0.411 | (0.787, 1.103) |
| F51 | Sleep disorders not due to a substance or known physiological condition | 1.133 | 0.125 | 0.070 | 1.771 | 0.077 | (0.987, 1.300) |
| F10-F19 | Mental and behavioral disorders due to psychoactive substance use | 1.151 | 0.141 | 0.060 | 2.351 | 0.019 | (1.024, 1.294) |
| F30-F39 | Mood [affective] disorders | 2.696 | 0.992 | 0.051 | 19.412 | 0 | (2.439, 2.980) |
| F60.3 | Borderline personality disorder | 1.417 | 0.348 | 0.092 | 3.781 | 0.000 | (1.183, 1.697) |

**SA8.4: Mood Stabilizers**

| **Cox Model Results** | | | | | | | |
| --- | --- | --- | --- | --- | --- | --- | --- |
|  | **Covariate** | **Hazard Ratio** | **Coefficient** | **Standard Error** | **z** | **P > \|z\|** | **95% Confidence Interval** |
|  | Cohort 1 or Cohort 2 Membership | 0.517 | -0.660 | 0.073 | -9.076 | 0.000 | (0.448, 0.596) |
| M | Male | 1.010 | 0.010 | 0.073 | 0.131 | 0.896 | (0.875, 1.165) |
| AI | Age at Index | 1.136 | 0.128 | 0.013 | 9.482 | 0.000 | (1.107, 1.167) |
| 2054-5 | Black or African American | 1.027 | 0.026 | 0.090 | 0.293 | 0.769 | (0.861, 1.224) |
| 2186-5 | Not Hispanic or Latino | 1.065 | 0.063 | 0.075 | 0.846 | 0.398 | (0.920, 1.233) |
| F41.9 | Anxiety disorder, unspecified | 0.857 | -0.155 | 0.073 | -2.130 | 0.033 | (0.743, 0.988) |
| F91 | Conduct disorders | 1.519 | 0.418 | 0.073 | 5.767 | 0.000 | (1.318, 1.751) |
| F41.1 | Generalized anxiety disorder | 0.788 | -0.238 | 0.084 | -2.854 | 0.004 | (0.669, 0.928) |
| F41.0 | Panic disorder [episodic paroxysmal anxiety] | 1.131 | 0.123 | 0.126 | 0.980 | 0.327 | (0.884, 1.448) |
| F42 | Obsessive-compulsive disorder | 1.299 | 0.262 | 0.130 | 2.010 | 0.044 | (1.007, 1.676) |
| F70-F79 | Intellectual Disabilities | 1.429 | 0.357 | 0.162 | 2.199 | 0.028 | (1.040, 1.964) |
| F50 | Eating disorders | 1.084 | 0.081 | 0.134 | 0.604 | 0.546 | (0.833, 1.411) |
| F51 | Sleep disorders not due to a substance or known physiological condition | 0.821 | -0.197 | 0.140 | -1.405 | 0.160 | (0.624, 1.081) |
| F10-F19 | Mental and behavioral disorders due to psychoactive substance use | 0.974 | -0.026 | 0.101 | -0.261 | 0.794 | (0.799, 1.187) |
| F30-F39 | Mood [affective] disorders | 2.285 | 0.826 | 0.093 | 8.855 | 0.000 | (1.903, 2.744) |
| F60.3 | Borderline personality disorder | 1.481 | 0.393 | 0.149 | 2.631 | 0.009 | (1.105, 1.985) |

**Supplement Analysis 9. Association Between ADHD Treatment and Subsequent PTSD Diagnosis**

**SA9.1. Cohorts: ADHD Medications (vs No ADHD Medications)**

### Query Criteria for Cohort 1 (query name: On ADHD med)

This query was run on the network Research with 102 HCO(s) queried and 102 HCO(s) responded. A total of 82 provider(s) responded with patients. The final cohort included 450,079 patients who matched the query criteria listed in the table below.

| Cohort 1 | | | | | |
| --- | --- | --- | --- | --- | --- |
|  | must have |  | demographics | Age | Age (between 6 and 18 years (most recent occurrence)) |
|  |  | and | diagnosis | UMLS:ICD10CM:F90 | Attention-deficit hyperactivity disorders |
|  |  | and any of | medication | NLM:VA:CN801 | AMPHETAMINES |
|  |  |  | medication | NLM:VA:CN802 | AMPHETAMINE LIKE STIMULANTS |
|  |  |  | medication | NLM:RXNORM:2599 | Clonidine |
|  |  |  | medication | NLM:RXNORM:11196 | Viloxazine |
|  |  |  | medication | NLM:RXNORM:40114 | guanfacine |
|  |  |  | medication | NLM:RXNORM:38400 | atomoxetine |

### Query Criteria for Cohort 2 (query name: Not on ADHD med)

This query was run on the network Research with 102 HCO(s) queried and 102 HCO(s) responded. A total of 92 provider(s) responded with patients. The final cohort included 320,830 patients who matched the query criteria listed in the table below.

| Cohort 2 | | | | | |
| --- | --- | --- | --- | --- | --- |
|  | must have |  | demographics | Age | Age (between 6 and 18 years (most recent occurrence)) |
|  |  | and | diagnosis | UMLS:ICD10CM:F90 | Attention-deficit hyperactivity disorders |
|  | cannot have |  | medication | NLM:VA:CN801 | AMPHETAMINES |
|  |  | or | medication | NLM:VA:CN802 | AMPHETAMINE LIKE STIMULANTS |
|  |  | or | medication | NLM:RXNORM:38400 | atomoxetine |
|  |  | or | medication | NLM:RXNORM:11196 | Viloxazine |
|  |  | or | medication | NLM:RXNORM:2599 | Clonidine |
|  |  | or | medication | NLM:RXNORM:40114 | guanfacine |

| **Cohort Information** | | |
| --- | --- | --- |
|  | **Cohort Name** | **Index Count** |
|  | On ADHD med | 422450 |
|  | Not on ADHD med | 306123 |

| Outcome | | | | |
| --- | --- | --- | --- | --- |
|  | **Outcome definition** | | | |
|  | | Diagnosis | UMLS:ICD10CM:F43.1 | Post-traumatic stress disorder (PTSD) |

| **Cox Model Results** | | | | | | | |
| --- | --- | --- | --- | --- | --- | --- | --- |
|  | **Covariate** | **Hazard Ratio** | **Coefficient** | **Standard Error** | **z** | **P > \|z\|** | **95% Confidence Interval** |
|  | Cohort 1 or Cohort 2 Membership | 1.732 | 0.549 | 0.016 | 34.593 | 0 | (1.679, 1.787) |
| M | Male | 0.537 | -0.623 | 0.014 | -45.795 | 0 | (0.522, 0.551) |
| AI | Age at Index | 1.151 | 0.141 | 0.002 | 65.284 | 0 | (1.146, 1.156) |
| 2054-5 | Black or African American | 1.295 | 0.258 | 0.017 | 15.363 | 0 | (1.253, 1.338) |
| 2186-5 | Not Hispanic or Latino | 1.063 | 0.061 | 0.015 | 4.056 | 0.000 | (1.032, 1.095) |

**SA9.2. Cohorts: CNS stimulants (vs Non-stimulants)**

### Query Criteria for Cohort 1 (query name: ST, not on NS)

This query was run on the network Research with 102 HCO(s) queried and 102 HCO(s) responded. A total of 76 provider(s) responded with patients. The final cohort included 233,310 patients who matched the query criteria listed in the table below.

| Cohort 1 | | | | | |
| --- | --- | --- | --- | --- | --- |
|  | must have |  | demographics | Age | Age (between 6 and 18 years (most recent occurrence)) |
|  |  | and | diagnosis | UMLS:ICD10CM:F90 | Attention-deficit hyperactivity disorders |
|  |  | and any of | medication | NLM:VA:CN801 | AMPHETAMINES |
|  |  |  | medication | NLM:VA:CN802 | AMPHETAMINE LIKE STIMULANTS |
|  | cannot have |  | medication | NLM:RXNORM:2599 | clonidine |
|  |  | or | medication | NLM:RXNORM:11196 | viloxazine |
|  |  | or | medication | NLM:RXNORM:38400 | atomoxetine |
|  |  | or | medication | NLM:RXNORM:40114 | guanfacine |

### Query Criteria for Cohort 2 (query name: NS, not on ST)

This query was run on the network Research with 102 HCO(s) queried and 102 HCO(s) responded. A total of 76 provider(s) responded with patients. The final cohort included 55,660 patients who matched the query criteria listed in the table below.

| Cohort 2 | | | | | |
| --- | --- | --- | --- | --- | --- |
|  | must have |  | demographics | Age | Age (between 6 and 18 years (most recent occurrence)) |
|  |  | and | diagnosis | UMLS:ICD10CM:F90 | Attention-deficit hyperactivity disorders |
|  |  | and any of | medication | NLM:RXNORM:40114 | guanfacine |
|  |  |  | medication | NLM:RXNORM:2599 | clonidine |
|  |  |  | medication | NLM:RXNORM:38400 | atomoxetine |
|  |  |  | medication | NLM:RXNORM:11196 | viloxazine |
|  | cannot have |  | medication | NLM:VA:CN801 | AMPHETAMINES |
|  |  | or | medication | NLM:VA:CN802 | AMPHETAMINE LIKE STIMULANTS |

| **Cohort Information** | | |
| --- | --- | --- |
|  | **Cohort Name** | **Index Count** |
|  | ST, not on NS | 217597 |
|  | NS, not on ST | 52019 |

| **Cox Model Results** | | | | | | | |
| --- | --- | --- | --- | --- | --- | --- | --- |
|  | **Covariate** | **Hazard Ratio** | **Coefficient** | **Standard Error** | **z** | **P > \|z\|** | **95% Confidence Interval** |
|  | Cohort 1 or Cohort 2 Membership | 0.241 | -1.422 | 0.025 | -57.907 | 0 | (0.230, 0.253) |
| M | Male | 0.461 | -0.773 | 0.025 | -31.271 | 0 | (0.440, 0.484) |
| AI | Age at Index | 1.182 | 0.167 | 0.004 | 42.910 | 0 | (1.173, 1.191) |
| 2054-5 | Black or African American | 1.357 | 0.305 | 0.031 | 9.787 | 0.000 | (1.276, 1.442) |
| 2186-5 | Not Hispanic or Latino | 1.010 | 0.010 | 0.027 | 0.371 | 0.710 | (0.958, 1.065) |

Supplement Analysis 10. Treatment Pathways Analysis Report

**SA10.1. Treatment Distribution Across Lines of Treatment in Individuals with ADHD and PTSD**

### Query Criteria for the Cohort (query name: ADHD+PTSD)

This query was run on the network Research with 100 HCO(s) queried and 100 HCO(s) responding. A total of 68 provider(s) responded with patients. The final cohort included 30,341 patients who matched the query criteria listed in the table below.

| Cohort | | | | | |
| --- | --- | --- | --- | --- | --- |
|  | must have |  | demographics | Age | Age (between 6 and 18 years (most recent occurrence)) |
|  |  | and | diagnosis | UMLS:ICD10CM:F90 | Attention-deficit hyperactivity disorders |
|  |  | and | diagnosis | UMLS:ICD10CM:F43.1 | Post-traumatic stress disorder (PTSD) |

### Treatment Definitions

Table below outlines the definitions for each treatment. For treatment definitions consisting of more than one term, at least one term must match.

| CNS Stimulants | | | | |
| --- | --- | --- | --- | --- |
|  | **Treatment definition** | | | |
|  | | Medication | NLM:VA:CN801 | AMPHETAMINES |
|  | | Medication | NLM:VA:CN802 | AMPHETAMINE LIKE STIMULANTS |
| Non-stimulants | | | | |
|  | **Treatment definition** | | | |
|  | | Medication | NLM:RXNORM:2599 | clonidine |
|  | | Medication | NLM:RXNORM:38400 | atomoxetine |
|  | | Medication | NLM:RXNORM:40114 | guanfacine |
|  | | Medication | NLM:RXNORM:11196 | viloxazine |
| Mood Stabilizers | | | | |
|  | **Treatment definition** | | | |
|  | | Medication | NLM:VA:CN750 | LITHIUM SALTS |
|  | | Medication | NLM:RXNORM:2002 | carbamazepine |
|  | | Medication | NLM:RXNORM:40254 | valproate |
|  | | Medication | NLM:RXNORM:28439 | lamotrigine |
|  | | Medication | NLM:RXNORM:32624 | oxcarbazepine |
| Antipsychotics | | | | |
|  | **Treatment definition** | | | |
|  | | Medication | NLM:VA:CN700 | ANTIPSYCHOTICS |
| Psychotherapy | | | | |
|  | **Treatment definition** | | | |
|  | | Procedure | UMLS:CPT:1021137 | Psychotherapy Services and Procedures |
| Antidepressants | | | | |
|  | **Treatment definition** | | | |
|  | | Medication | NLM:VA:CN600 | ANTIDEPRESSANTS |

### Criteria for Lines of Treatment

A line includes any treatment taken within 1 days.

The line ends

1. When a new treatment appears in the patient record after the first 1 days
2. When a patient dies
3. When a patient’s medical record ends
4. When the analysis time window ends

**SA10.2. CNS Stimulants Distribution Across Lines of Treatment by Cohort**

### Treatment Definitions

| Methylphenidate | | | | |
| --- | --- | --- | --- | --- |
|  | **Treatment definition** | | | |
|  | | Medication | NLM:RXNORM:6901 | methylphenidate |
| Dexmethylphenidate | | | | |
|  | **Treatment definition** | | | |
|  | | Medication | NLM:RXNORM:352372 | dexmethylphenidate |
| Mixed Amphetamine Salts | | | | |
|  | **Treatment definition** | | | |
|  | | Medication | NLM:RXNORM:3288 | dextroamphetamine |
|  | | Medication | NLM:RXNORM:725 | amphetamine |
| Lisdexamfetamine | | | | |
|  | **Treatment definition** | | | |
|  | | Medication | NLM:RXNORM:700810 | lisdexamfetamine |

**SA10.21. CNS Stimulants Distribution Across Lines of Treatment by ADHD without PTSD Cohort**

### Query Criteria for the Cohort (query name: ADHD)

This query was run on the network Research with 100 HCO(s) queried and 100 HCO(s) responding. A total of 92 provider(s) responded with patients. The final cohort included 683,788 patients who matched the query criteria listed in the table below.

| Ungrouped terms | | | | | |
| --- | --- | --- | --- | --- | --- |
|  | must have |  | demographics | Age | Age (between 6 and 18 years (most recent occurrence)) |
|  |  | and | diagnosis | UMLS:ICD10CM:F90 | Attention-deficit hyperactivity disorders |
|  | cannot have |  | diagnosis | UMLS:ICD10CM:F43.1 | Post-traumatic stress disorder (PTSD) |

**SA10.22. CNS Stimulants Distribution Across Lines of Treatment by ADHD with PTSD Cohort**

### Query Criteria for the Cohort (query name: ADHD+PTSD)

This query was run on the network Research with 100 HCO(s) queried and 100 HCO(s) responding. A total of 68 provider(s) responded with patients. The final cohort included 30,341 patients who matched the query criteria listed in the table below.

| Cohort | | | | | |
| --- | --- | --- | --- | --- | --- |
|  | must have |  | demographics | Age | Age (between 6 and 18 years (most recent occurrence)) |
|  |  | and | diagnosis | UMLS:ICD10CM:F90 | Attention-deficit hyperactivity disorders |
|  |  | and | diagnosis | UMLS:ICD10CM:F43.1 | Post-traumatic stress disorder (PTSD) |
|  | | | | | |

**SA10.3. Non-stimulants Distribution Across Lines of Treatment by Cohort**

### Treatment Definitions

| Clonidine | | | | |
| --- | --- | --- | --- | --- |
|  | **Treatment definition** | | | |
|  | | Medication | NLM:RXNORM:2599 | clonidine |
| Guanfacine | | | | |
|  | **Treatment definition** | | | |
|  | | Medication | NLM:RXNORM:40114 | guanfacine |
| Atomoxetine | | | | |
|  | **Treatment definition** | | | |
|  | | Medication | NLM:RXNORM:38400 | atomoxetine |
| Viloxazine | | | | |
|  | **Treatment definition** | | | |
|  | | Medication | NLM:RXNORM:11196 | viloxazine |

**SA10.31. Non-stimulants Distribution Across Lines of Treatment by ADHD without PTSD Cohort**

### Query Criteria for the Cohort (query name: ADHD)

This query was run on the network Research with 100 HCO(s) queried and 100 HCO(s) responding. A total of 92 provider(s) responded with patients. The final cohort included 683,788 patients who matched the query criteria listed in the table below.

| Cohort | | | | | |
| --- | --- | --- | --- | --- | --- |
|  | must have |  | demographics | Age | Age (between 6 and 18 years (most recent occurrence)) |
|  |  | and | diagnosis | UMLS:ICD10CM:F90 | Attention-deficit hyperactivity disorders |
|  | cannot have |  | diagnosis | UMLS:ICD10CM:F43.1 | Post-traumatic stress disorder (PTSD) |

**SA10.32. Non-stimulants Distribution Across Lines of Treatment by ADHD with PTSD Cohort**

### Query Criteria for the Cohort (query name: ADHD+PTSD)

This query was run on the network Research with 100 HCO(s) queried and 100 HCO(s) responding. A total of 68 provider(s) responded with patients. The final cohort included 30,341 patients who matched the query criteria listed in the table below.

| Cohort | | | | | |
| --- | --- | --- | --- | --- | --- |
|  | must have |  | demographics | Age | Age (between 6 and 18 years (most recent occurrence)) |
|  |  | and | diagnosis | UMLS:ICD10CM:F90 | Attention-deficit hyperactivity disorders |
|  |  | and | diagnosis | UMLS:ICD10CM:F43.1 | Post-traumatic stress disorder (PTSD) |
